# A Collective Study on Modeling and Simulation of Resistive Random Access Memory

**DOI:** 10.1186/s11671-017-2419-8

**Published:** 2018-01-10

**Authors:** Debashis Panda, Paritosh Piyush Sahu, Tseung Yuen Tseng

**Affiliations:** 1 Department of Electronics and Communication Engineering, National Institute of Science and Technology, Berhampur, Odisha 761008 India; 2 Nanoscale Science & Technology Lab, Department of EECS, National Chiao Tung University, Hsinchu, 30010 Taiwan; 3 Department of Electronics Engineering & Institute of Electronics, National Chiao Tung University, Hsinchu, 30010 Taiwan

## Abstract

In this work, we provide a comprehensive discussion on the various models proposed for the design and description of resistive random access memory (RRAM), being a nascent technology is heavily reliant on accurate models to develop efficient working designs and standardize its implementation across devices. This review provides detailed information regarding the various physical methodologies considered for developing models for RRAM devices. It covers all the important models reported till now and elucidates their features and limitations. Various additional effects and anomalies arising from memristive system have been addressed, and the solutions provided by the models to these problems have been shown as well. All the fundamental concepts of RRAM model development such as device operation, switching dynamics, and current-voltage relationships are covered in detail in this work. Popular models proposed by Chua, HP Labs, Yakopcic, TEAM, Stanford/ASU, Ielmini, Berco-Tseng, and many others have been compared and analyzed extensively on various parameters. The working and implementations of the window functions like Joglekar, Biolek, Prodromakis, etc. has been presented and compared as well. New well-defined modeling concepts have been discussed which increase the applicability and accuracy of the models. The use of these concepts brings forth several improvements in the existing models, which have been enumerated in this work. Following the template presented, highly accurate models would be developed which will vastly help future model developers and the modeling community.

## Background

This new age of computing requires a technology being equally capable to match its growth. The new technology should be able to meet the demands of improved performance and scalable to cater to the future devices. Memristors, postulated in 1971 [1] by Leon O. Chua seems to fulfill these requirements and laid the foundation for new classes of devices. Memristors, short for “memory-resistors,” are basic two-terminal devices which remember their internal resistance state depending on the history of the input stimulus provided. Chua devised that the memristors are characterized by a relationship between flux and charge, which are the time integrals of current and voltage, respectively.

Later in 1976, Chua and Kang [2] generalized the memristors to include in a new class of dynamical systems called memristive systems. In the end of twentieth century, the interest in these devices had waned despite its many benefits. This was partly because of the advances in silicon integrated circuit technology. But with the aging on silicon technologies and their incapability to support scaling down, the search for alternative switching devices gained attraction in the early twenty-first century. It was equally aided by the advances in the growth and characterization of nanoscale materials. This invariably leads to significant progress in understanding microscopic memristive switching.

Memristor technology got a major breakthrough in the year 2008 when Strukov et al. [3] established a link between the theory and experiment for their TiO_*x*_-based devices. Also, they obtained a pinched hysteresis in the current-voltage relationship, which is one of the identifiable features of memristive systems [4, 5]. This opened up the memristor technology to a wide array of devices following the footprints of the metal/oxide film/metal structure. Some of the similar types of popular devices were Oxygen RRAM (OxRRAM) [6–10] and Conductive Bridge RAM (CBRAM) [11–13] among many others. These devices are generally classified on the basis of their switching mechanism.

### Resistive Random Access Memory (RRAM)

Research interest into these emerging devices heightened because the non-volatile memristive behavior demonstrated could be harnessed into non-volatile memory. They are being seen as potential alternatives of the flash memory technology. With present age computing being more and more data driven, there has been demands for a memory technology which is more in-tune with the present and future requirements. Compared to the several emerging devices, RRAM devices are more scalable [14–18], have high density [19–24], consume low power [25–29], are faster [30–33], have higher endurance and retention [34–37] and highly CMOS compatible [38–42]. RRAM devices are one of the most popular non-volatile memory technologies with extensive study being undertaken to understand their mechanism and develop models to realize the device operation and design accurate and simple device structure. The devices are simple two-terminal metal-insulator-metal (MIM) structure and switch between two resistance states low-resistance state (LRS) and high-resistance state (HRS). A LRS suggests the device is in the SET or ON state. A contrasting HRS means the device is in the RESET or OFF state. Through this switching of resistance states in the device, the data bit is stored [43–45]. RRAM devices can be classified into bipolar and unipolar devices, depending on the polarity of switching. In unipolar switching, the devices switch in the same polarity bias, whereas in bipolar switching, bias of both the polarities is required.

Several approaches have been proposed to explain the switching mechanism of RRAM devices, but the most popular and widely accepted, for binary oxide-based RRAM devices, is the formation and ruptured of localized conductive filaments (CF) by the drift of oxygen ions/ vacancies [9, 16, 46–49]. The SET/RESET occurs as a result of the combination/re-generation of the oxygen ions/vacancies [50–52]. It has been demonstrated that the performance of the RRAM devices is strongly affected by the choice of the active oxide layer [53–55]. A variety of oxide systems such as HfO_*x*_, TiO_*x*_, NiO_*x*_, TaO_*x*_, ZnO_*x*_, etc. [56–66] have been used to demonstrate resistive switching behavior. There have been some controversies whether RRAM devices are actually memristive devices. To make the position of RRAM devices clear, Chua provided clarifications that they are indeed memristive devices [67].

### Importance of RRAM Modeling

A very important aspect of developing electronic devices based on new semiconductor technologies is the role of modeling. An accurate and comprehensive model is of paramount importance in understanding the device operation, designing it for optimum performance, and verifying that it matches the required specifications. A number of models have been proposed with varying degrees of accuracy, different features, and mixed results. So, any developer aiming to design a robust and flexible model for RRAM devices should have information about the methods tried before and the constraints faced.

In this work, we have discussed in detail all the features and characteristics of the various RRAM models. General memristor models are also considered to explain RRAM devices [67]. Starting from the Chua model [1] which provides the basics of memristors, we discuss the fundamental definition of memristors. The breakthrough for memristors and RRAM devices provided by the HP model [3] is discussed in detail. Linear ion drift effects, which form the basics of the mechanism of these devices, along with the non-linear effects [46, 68, 69], are considered. The Pickett-Abdalla model [70–72] which laid the foundation for SPICE compatible physics-based models is covered in-depth. Its various features which have been adopted and refined by the Yakopcic model [73, 74] are also covered.

Models which introduced new features such as threshold effects [75–77], taking filament gap as the state variable [78–81], have been reviewed. Some of the models which account for unipolar devices and temperature effects [82–84] are reviewed in detail. Also considered are physical models [85, 86] based on the device growth dynamics. Along with these, models considering only bipolar devices [87–89], change of CF size [90, 91], and many other factors [92, 93] are taken into account. A concise analysis of all the discussed models has been presented in Table 1.

**Table 1:** Comparative analysis of the models

Model	Device type	State variable	Control mechanism	Threshold exists	Supports boundary effects	Simulation compatible
Chua model [1, 2]	Generic	Flux or charge	Current	NA	NA	NA
Linear ion drift [3]	Bipolar	0 ≤ *w* ≤ *D* Doped region physical width	Current	No	External window functions	Possible with SPICE
Non-linear ion drift [46, 68]	Bipolar	0 ≤ *w* ≤ 1 Doped region normalized width	Voltage	No	External window functions	No
Exponential [69]	Bipolar	Switching speed	Voltage	No	Yes	No
Simmons tunneling barrier [70–72]	Bipolar	*a*_off_ ≤ *w* ≤ *a*_on_ Undoped region width	Current	No	No	SPICE
Yakopcic [73, 74]	Bipolar	0 ≤ *w* ≤ 1 Not explained physically	Voltage	Yes	External window functions	SPICE/Verilog/MAPP
TEAM [75, 76]	Bipolar	*x*_on_ ≤ *x* ≤ *x*_off_ Undoped region width	Current	Current	Implicit window functions	SPICE/Verilog/MAPP
VTEAM [77]	Bipolar	*x*_on_ ≤ *x* ≤ *x*_off_ Undoped region width	Voltage	Voltage	Implicit window functions	SPICE/Verilog/MAPP
ASU/Stanford [78–81]	Bipolar	Filament gap (*g*)	Voltage	Temperature	No	SPICE/Verilog/MAPP
Filament dissolution [82–86]	Unipolar	Concentration of ions	Voltage	Temperature	No	COMSOL
Physical electro thermal [87]	Bipolar	Concentration of ions	Voltage	Temperature	Practically yes	COMSOL
Bocquet unipolar [90]	Unipolar	Concentration of ions	Voltage	Temperature	Yes	COMSOL/SPICE
Bocquet bipolar [91, 92]	Bipolar	CF radius	Voltage	Temperature	Yes	SPICE
Gonzalez-Cordero [93]	Bipolar	CF radius (top and bottom)	Voltage	Temperature	Yes	SPICE

Various models based on window function implementations such as Joglekar [94], Biolek [95], Benderli-Wey [96], Shin [97], Prodromakis [98, 99], etc. have also been accounted for the limitations and constraints in the various models, and the methods used by subsequent models to overcome them have been presented in a comprehensive manner. Significant work done by Wang and Roychowdhury [100] to improve RRAM modeling has also been reviewed in depth as it is a considerable push in the right direction for the whole RRAM modeling community. Along with those examples, covering simulation and verification studies of the devices in different platforms are discussed. This is the most comprehensive review relating to RRAM and memristor models at present stage. The description of the models has been divided into those that describe bipolar devices and unipolar devices. Window function implementation models are described in a separate section.

Earlier, there have been multiple reviews on RRAM device mechanisms [46, 101–105], fabrication technology [106–109], material stacks [110–113], and a concise discussion on some of the models present at that time [114]. Very recently Villena et al. [115] combined the theory of all RRAM modeling and proposed an optimize model. In this study, we focused more on the various modeling techniques along with the solutions provided to various drawbacks. A comprehensive discussion on boundary condition models which can be classified as pseudo-compact models have also been discussed. Some critical modeling techniques have been investigated in this work which can significantly help model developers. Also, a discussion on various simulation techniques and platforms for RRAM models such as SPICE [116, 117] has been included which is highly essential. Our work aims to fill a significant gap in the RRAM modeling community.

## RRAM Models for Bipolar Devices

### Chua Model

Leon O. Chua in 1971 put forward the idea of memristor [1] that it was indeed the fourth basic element alongside the resistor, capacitor, and inductor. The basic characteristics of a memristor are believed to be flux controlled (*φ*) or charge controlled (*q*) and are defined by a relation of the type g (*φ,q*) = 0.

Chua defined the voltage of a memristor as [1]:

1 $$ v(t)=M\left(q(t)i(t)\right) $$

where

2 $$ M(q)= d\varphi (q)/ dq $$

The current flowing through a flux-controlled memristor was formulated as^1^:

3 $$ i(t)=W\left(\varphi (t)v(t)\right) $$

where

4 $$ W\left(\varphi \right)= dq\left(\varphi \right)/ d\varphi $$

**Fig. 1:**
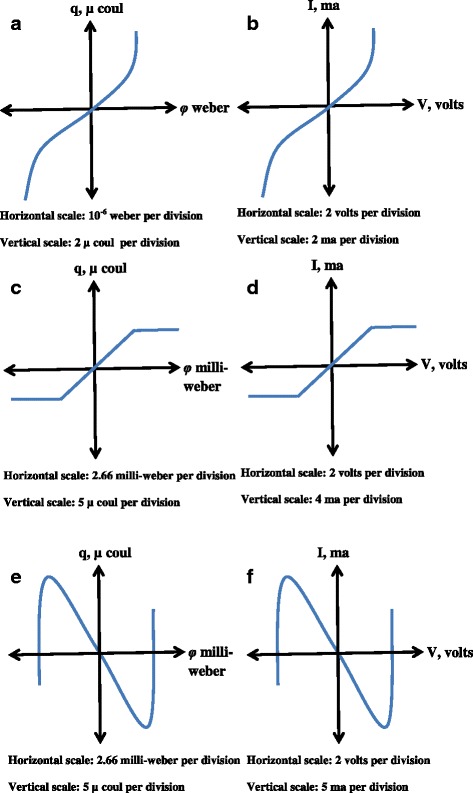
**a**–**f** Flux-charge (*ϕ*-*q*) curves obtained from three different memristors [1]

Here, the parameters *M*(*q*) and *W*(*φ*) are defined as incremental memristance and incremental memductance, respectively, owing to them having units similar to resistance and conductance. The *φ-q* curves for the three memristor devices are shown in Fig. 1. These curves are generated by a basic memristor-resistor (M-R) circuit which gives rise to three types of memristors. The *φ-q* variance for those devices is shown in Fig. 1a–e, respectively. Figure 1b–f depicts the corresponding *I-V* relations of the same three memristors.

The equations presented above can be simplified into the following [1]:

5 $$ v=R(w)\times i $$

6 $$ \frac{dw}{dt}=i $$

where *w* is the state variable of the device and *R* a generalized resistance that depends upon the internal state of the device.

The value of incremental memristance (memductance) at a time instant *t*_0_ depends on the time integration of the complete memristor current (voltage) from *t* = − *t* to *t* = *t*_0_. So, this translates to the fact that while a memristor acts as a normal resistor at any instant of time *t*_0_, but its resistance (conductance) values depend on the complete past history of the device current (voltage), hence the justification of the name memory resistor.

Interestingly, at the time of specified memristor voltage *v*(*t*) or current *i*(*t*), the memristor behaves as a linear time-varying resistor. But in the case when the *φ-q* curve is a straight line, i.e., *M*(*q*) *= R* or *W*(*φ*) *= G*, the memristor acts like a linear time-invariant resistor. So, a memristor device cannot be used in linear network theory but can be used to define circuits where the present state of the parameters is dependent on the past states.

Later, in 1976, Chua and Kang [2] generalized the memristor concept to include memristive systems which include many non-linear dynamic systems. It was described by the equations [2]:

7 $$ v=R\left(w,i\right)\times i $$

8 $$ \frac{dw}{dt}=f\left(w,i\right) $$

where *w* is defined as a set of state variables, *R* and *f* are explicit functions of time. A basic difference between memristors and memristive systems is that in the later the flux is no longer uniquely defined by the charge. Memristive systems can be distinguished from a general dynamic system in that there is no current flowing in the device when the voltage drop across it is zero.

The memristor equations were used reasonably to define the variable state of a threshold switch by Chua [1], which are the first instance of using memristors in device modeling. Formulation of the memristor by Chua rightfully laid the foundation for a new class of devices and varied applications which use a basic circuit element to store data. This basic concept of memristors led to the design of new architectures for future non-volatile memory applications of which RRAM is a promising candidate. There has been significant amount of theories explaining the working of RRAM devices and models defining them, which are fundamentally based on the memristor model.

A very interesting application of the flux-charge model is its use [118] to define a unipolar RRAM and implement it in SPICE. Owing to the simplicity of the flux-charge equations, they can be easily integrated into circuit simulators with few modifications. SPICE model was tested against experimental data of HfO_2_-based unipolar RRAM device. The non-linear relation proposed to fit the experimentally obtained normalized *q*-*φ* values is given as [118]:

9 $$ q\left(\varphi \right)={q}_r\times \min \left(1,{\left(\frac{\varphi }{\varphi_r}\right)}^n\right) $$

Here, *φ*_*r*_ is the flux at the RESET point. When this value *q*(*φ*) = *q*_*r*_ is obtained, the CF disappears and the current associated with the CF is set back to 0. This translates to the device being in the HRS. To investigate the ability of the model to reproduce unipolar switching characteristics of the device, a standard bias sweep operation is performed. The voltage applied on the device at reset state is increased progressively from zero bias until it reaches the LRS and then the bias is swept back to zero volts. The LRS current is modeled using a modified form of the current relation of the Chua model [1], given as [118]:

10 $$ i(t)=\left\{\begin{array}{c}K\sqrt{\varphi }v(t)\kern0.75em \mathrm{if}\ \varphi <{\varphi}_r\\ {}0\kern4.25em \mathrm{if}\ \varphi ={\varphi}_r\end{array}\right. $$

HRS current is assumed to be controlled by a thermionic emission, so the current in that state is modeled as:

11 $$ i(v)={I}_A\left({e}^{\frac{v}{v_A}}-1\right) $$

Threshold effects are also considered in the model. It has been assumed that the threshold voltage effect arises due to contact effects. It can be taken into account by including a voltage threshold for the flux computation in both the SET and RESET processes. The modified current is given by [118]:

12 $$ i(t)=\left\{\begin{array}{c}{I}_A\left({e}^{\frac{v}{v_A}}-1\right)\kern2.75em \mathrm{if}\ \varphi <{\varphi}_s\\ {}K\sqrt{\varphi }v(t)\kern3.75em \mathrm{if}\ \varphi <{\varphi}_r\end{array}\right. $$

Here, *ϕ*_*r*_ and *ϕ*_*s*_ are the RESET and SET flux, respectively. These equations can be implemented into a SPICE-compatible circuit comprising of a network of capacitors. The SPICE implementation results were found to be closely following the experimental results with the model able to reproduce almost identical memristor characteristics. It validates the use of the Chua flux-charge model [1] to be used for modeling unipolar devices as well.

### Linear Ion Drift Model

With a considerable gap in the consequent decades after the formulation of the memristor by Chua, researchers at HP Labs [3] in 2008 made an exciting find regarding memristor devices. Although Chua had formulated the presence of an element such as a memristor, there had not been a realizable circuit or model developed after that although several efforts were reported to fabricate RRAM devices in the very beginning of twenty-first century. The team at HP Labs led by Strukov et al. [3] realized a functional nanoscale memristive system where memristance occurs naturally, where solid-state electronic and ionic transport are coupled together under an external voltage bias. Those systems show a hysteretic relation between the current and voltage characteristics similar to other nanoscale electronic devices, thus leading to a fundamental understanding of memristive systems and the design of similar systems.

A simple two-terminal device was reported, where an oxide (TiO_2_) of thickness *D* was sandwiched in between two Pt electrodes. Hysteresis *I*-*V* switching curves have been compared with the simulated curve. Although the exact mechanism of these devices was not completely understood at that time, it was one of the first instances where resistive switching memories were classified into memristive systems.

**Fig. 2:**
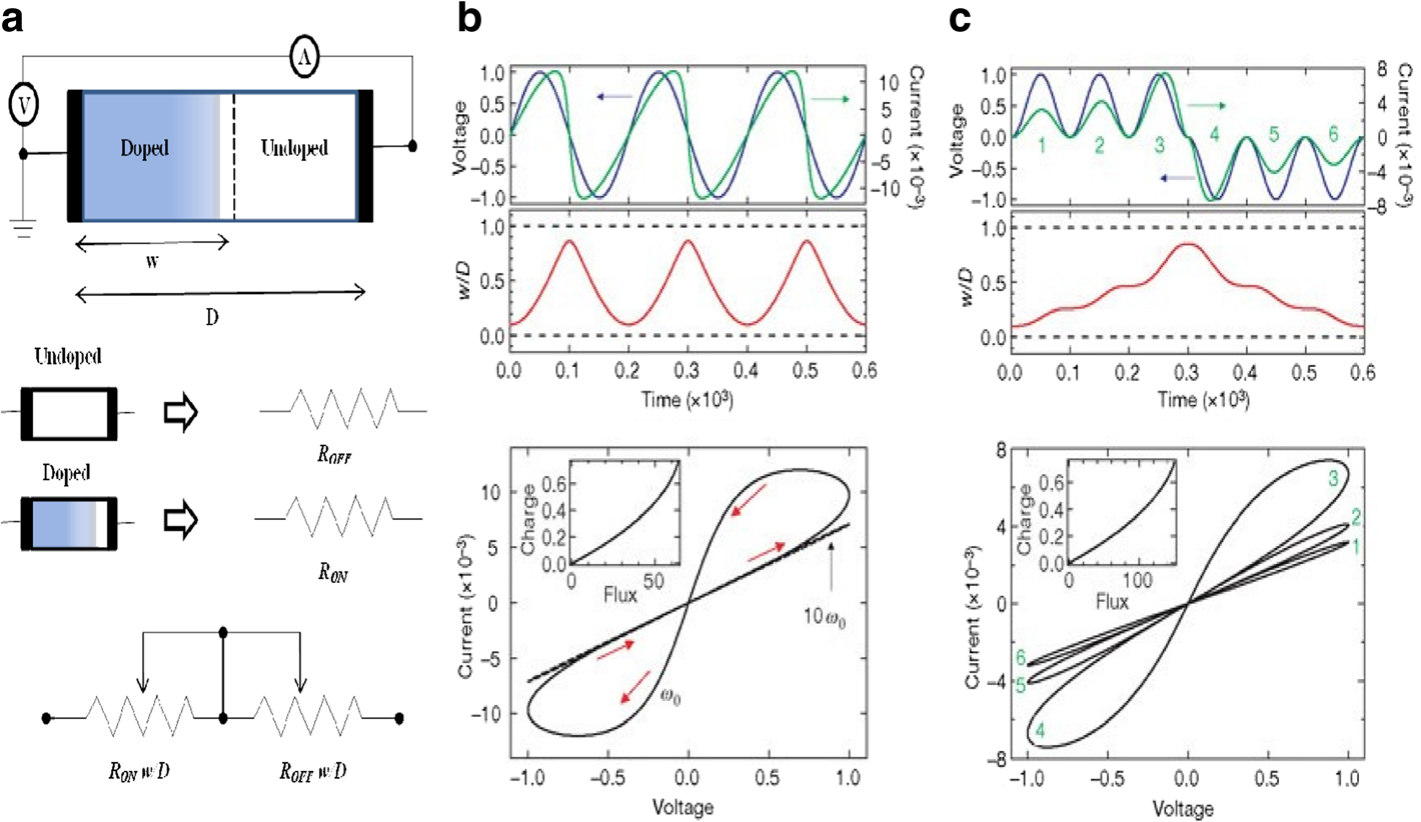
The coupled variable-resistor model for a memristor is presented. **a** A simplified equivalent circuit comprising of a (V) voltmeter and (A) ammeter. **b**, **c** The applied voltage (blue) and resulting current (green) as a function of time *t* for a typical memristor are also presented. In **b** the applied voltage is *v*_0_ sin(*v*_0_*t*) and the resistance ratio is *R*_OFF_/*R*_ON_ = 160, and in **c** the applied voltage is ±*v*_0_ sin^2^(ω_0_*t*) and *R*_OFF_/*R*_ON_ = 380, where ω_0_ is the frequency and *v*_0_ is the magnitude of the applied voltage. The numbers 1–6 are labeled for successive waves in the applied voltage and the corresponding loops in *i–v* curves. In each plot, the axes are dimensionless, with voltage, current, time, flux, and charge expressed in units of *v*_0_ = 1 V, *i*_0_ ≡ v_0_/*R*_ON_ = 10 mA, *t*_0_ ≡ 2*π*/ω_0_ ≡ *D*^2^/μ_v_*v*_0_ = 10 m/s, *v*_0_*t*_0_ and *i*_0_*t*_0_, respectively. The term *i*_0_ denotes the maximum possible current through the device, and *t*_0_ is the shortest time required for linear drift of dopants across the full device length in a uniform field *v*_0_/*D*, for example with *D* = 10 nm and μ_V_ = 10^−10^ cm^2^ s^−1^ V^−1^. It is to be noted that for the parameters chosen, the applied bias never forces either of the two resistive regions to collapse; for example, *w*/*D* does not approach zero or one (shown with dashed lines in the middle plots in **b** and **c**). Also, the dashed *i–v* plot in **b** demonstrates the hysteresis collapse observed with a tenfold increase in sweep frequency. The insets of *i–v* plots in **b** and **c** show that for these examples, the charge is a single-valued function of the flux, as it must be in a memristor [3]

A schematic device structure of TiO_2_-based memristor is shown in Fig. 2a [3], where there are two variable resistances in series, called as *R*_ON_ which is the low resistance in the semiconductor region with higher dopant concentration. A lesser dopant concentration makes the other part higher in resistance, called as *R*_OFF_. Relation between the applied voltage *v*(*t*) and current through the system *i*(*t*) owing to ohmic electronic conductance and linear ionic drift in a uniform field with average ion mobility is given by [3]:

13 $$ v(t)=\left(\frac{R_{\mathrm{ON}}w(t)}{D}+{R}_{\mathrm{OFF}}\left(1-\frac{w(t)}{D}\right)\right)i(t) $$

Although the equation above itself is non-linear, the resistance of the device linearly changes with the applied voltage *v*(*t*), thus the attribution of linearity to the model. Device defined by Strukov et al. [3] acts as a perfect memristor for only a particular bounded range of the state variable *w*. The state variable is defined as [3]:

14 $$ \frac{dw(t)}{dt}={\mu}_v\frac{R_{\mathrm{ON}}}{D}i(t) $$

Memristance of the system proposed by Chua [1] in Eq. (1) is defined by using the above two Eqs. (13) and (14) [3]:

15 $$ M(q)={R}_{\mathrm{OFF}}\left(1-\frac{\mu_v{R}_{\mathrm{ON}}}{D^2}q(t)\right) $$

In the above Eq. (15), the *q*-dependent term is the primary contribution to memristance. An interesting analysis provided as to why this particular phenomenon was hidden for so long is due to that magnetic field did not play an explicit role in the mechanism. For a memristor to be realized in simple terms, there should exist a non-linear relationship between the integrals of voltage and current.

The Eqs. (13)–(15) also incorporate the fundamentals of bipolar switching, that is the device switches from one state to another by the application of voltage of two polarities. As a result, devices showing bipolar hysteretic *I*-*V* relationships are capable of being modeled by these equations, and hence leading to the classification of such devices as memristive systems. Such behavior is observed in many material systems such as organic films [119–123], chalcogenides [124–126], metal oxides [127–129], dielectric oxides [130–132], perovskites [133–136], etc. The HP team themselves used a TiO_2_ [3] system and observed similar bipolar switching characteristics, with the dopant or impurity motion through the active region as the reason for such dramatic changes in the resistance. This is shown in Fig. 2b, c with the current showing drastic drop and rapid rise with the change in voltage.

**Fig. 3:**
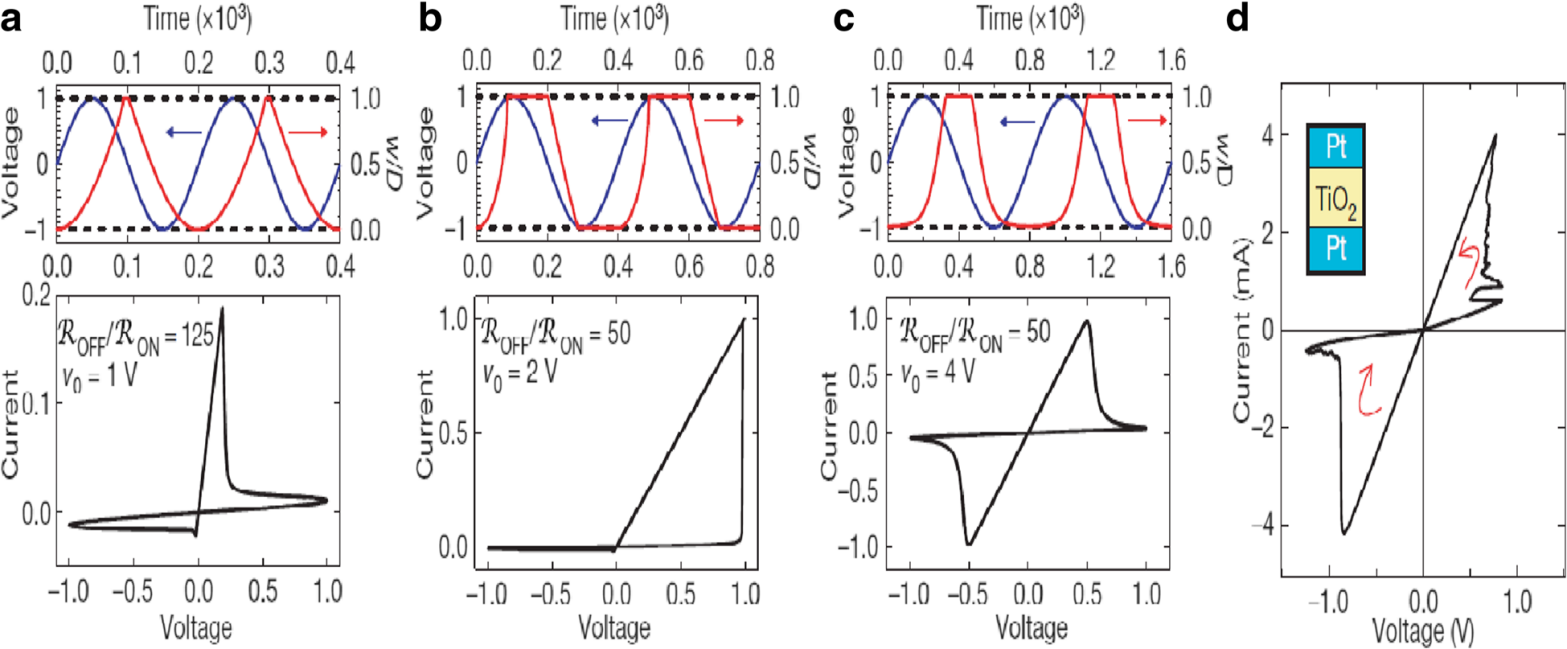
Simulated voltage-driven memristive device. **a** Simulation with dynamic negative differential resistance. **b** Simulation with no dynamic negative differential resistance. **c** Simulation governed by nonlinear ionic drift. In the upper plots of **a**, **b**, and **c**, the voltage stimulus (blue) and the corresponding change in the normalized state variable *w*/*D* (red) is plotted against time. In all cases, hard switching occurs when *w*/*D* closely approaches the boundaries at zero and one (dashed), and the qualitatively different *i*-*v* hysteresis shapes are due to the specific dependence of *w*/*D* on the electric field near the boundaries. **d** For comparison, an experimental *i–v* plot of a Pt–TiO_2 − *x*_–Pt device is presented [3]

Physically, the active region in these two terminal devices operates within the bound, 0 to *D*, the thickness of the oxide layer, so the state variable *w* is also bounded between the thicknesses. Figure 3 indicates the variation of *w/D* with time for the parameter never leaving the bounds of 0 and *D* [3]. The sudden change in resistance or the switching is caused by the devices reaching these bounds. In order to model this condition, suitable boundary conditions are used. Certain anomalies are observed in the device at the boundaries specifically. There is a non-constant change in the rate of the dynamic state variables over the available change. Also, the ion mobility is significantly less at the boundaries than in the middle. This is attributed to the non-linear dopant drift effects at the boundaries. Therefore, to properly account for these effects, the variations of certain window functions are used to define the bounds for the devices. HP team proposed a window function multiplied to the state variable Eq. (9) given as [3]:

16 $$ f(x)=\raisebox{1ex}{$w\left(1-w\right)$}\!\left/ \!\raisebox{-1ex}{${D}^2$}\right. $$

This model could be attributed to laying the foundations for future RRAM models. It can also be used for two terminal semiconductor devices having bipolar hysteretic *I*-*V* relationships. Taking the mechanism of a memristor as the reference, numerous future models for RRAM devices have been developed.

### Non-linear Ion Drift Model

Linear ion drift model developed by HP [3] primarily demonstrated linear drift effects in the bulk region of the memristor device. They observed some non-linear effects at the boundaries but did not define it comprehensively. Non-linear dependence of the dopant drift on applied voltage was observed and formulated by Yang et al. [46] in 2008. They proposed a current-voltage relationship accounted for the non-linear effects accurately. It was later improved and added upon by Eero Lehtonen and Mika Laiho [68].

Conduction in memristive devices is controlled by a spatially heterogeneous metal/oxide electronic barrier was reported by Yang et al. [46]. The switching is caused by the drift of positively charged oxygen vacancies acting as native dopants to form or dissolute conductive channels through this electronic barrier. The concentration of vacancies is higher at the boundaries or metal/oxide interfaces. The ON and OFF switching took place at the top interface only, which indicates that top electrode acts as the active electrode.

**Fig. 4:**
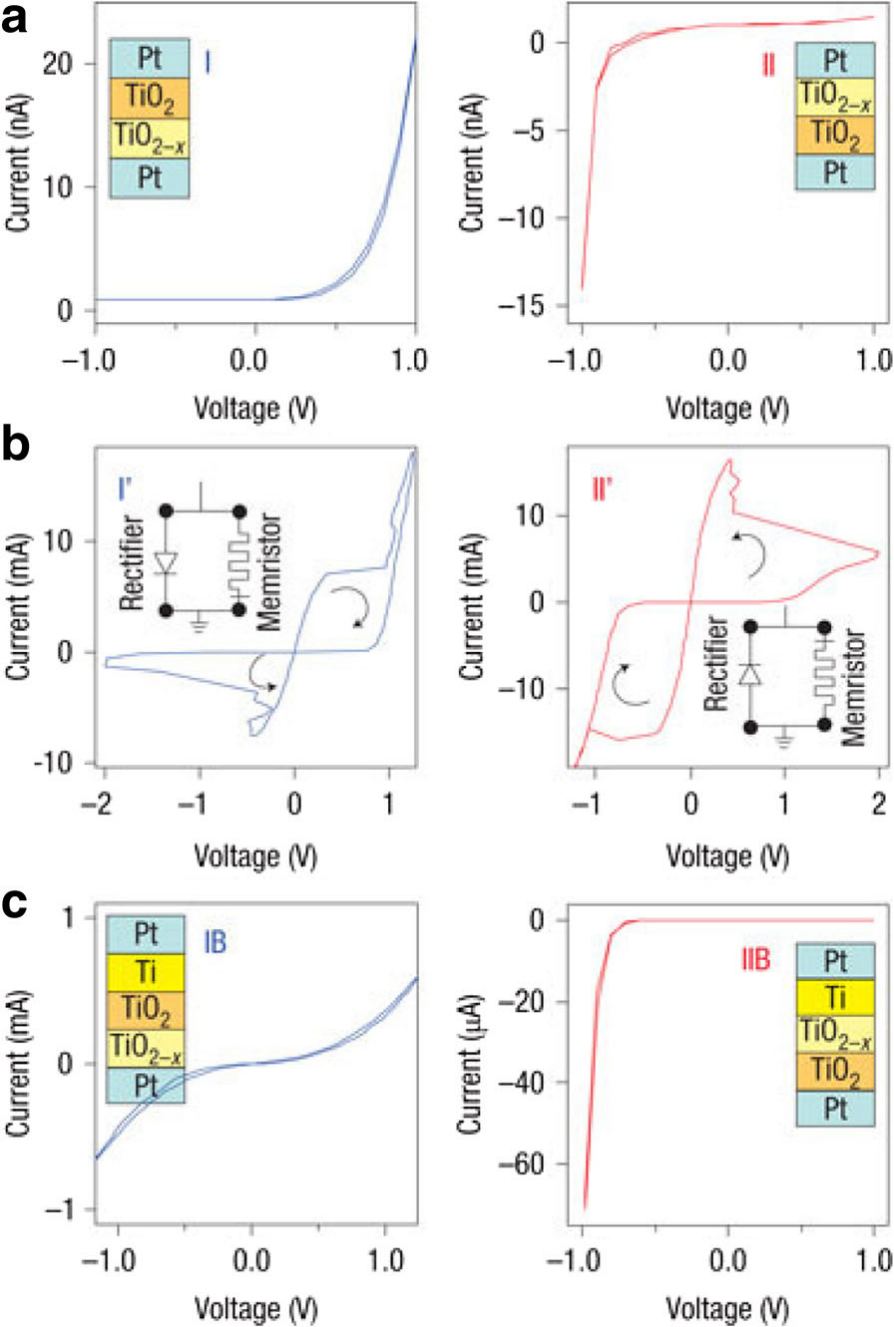
Thin-film TiO_2 − *x*_ devices with controlled oxygen vacancy profiles are used to verify the switching mechanism. **a** Samples I and II contain reversed layer sequences of 15-nm TiO_2_ and 15-nm TiO_2 − *x*_ (more vacancies) layers. These show opposite polarities of *I-V* curves in their virgin states. **b**. The switching polarities of these two samples are also opposite to each other. **c**. When more vacancies are introduced by adding a 5-nm Ti layer to the top interfaces of these two samples, the *I-V* curves change in totally different ways, confirming the dominant role of then non-ohmic interfaces in the thin-film devices [46]

The effect of oxygen vacancies on the switching characteristics of titanium oxide-based memristor is shown in Fig. 4 [46].The samples having different oxygen vacancies with different layer sequences of TiO_2_ show opposite switching defined by their polarities. Also, the addition of extra vacancies to the top interface, shown in Fig. 4c, changes the switching curves thus confirming the dominant role of non-ohmic interfaces in memristive devices. This forms the basis of the non-linearity effects that originate at the interfaces and govern the device switching.

Yang et al. [46] explained the above fact that the memristive devices act as dynamic resistors which change their state according to the time integral of the applied current or voltage; they failed to give a relationship describing a dynamic state variable. The proposed current-voltage relationship can be described as [46]:

17 $$ I={w}^n\beta \sinh \left(\alpha v\right)+\chi \left({e}^{\gamma v}-1\right) $$

Here, β, γ, *n*, and χ are fitting constants. In the above equation, the first term *β*sinh(*αv*) approximates [1] the ON state of the memristor where the electrons tunnel through the thin residual electronic barrier. *w* is defined as the state variable of the device in the range of 0 (OFF) and 1 (ON). Second part of the equation approximates the OFF state of the device with the other parameters acting as fitting constants. Parameter *n* here acts as the free parameter used to modify the switching between the states. During the adjustment of *n,* the non-linear effects come into picture. *I*-*V* curve from the fabricated device is modeled using the Eq. (16). The best fitting is obtained at 14 ≤ *n* ≤ 22. This can be interpreted as evidence that the effective vacancy drift velocity depends in a very highly non-linear way with the applied voltage to the device. So, the majority of the dopant drift effects at the boundaries/interfaces could then be understood as non-linear in nature.

A relationship describing the dynamics of the state variable *w* in this model using SPICE [116, 117] was proposed by Lehtonen and Laiho [68]. The time derivative of *w* was modeled as [68]:

18 $$ \frac{dw}{dt}=a\times f(w)\times g(v) $$

Here, *a* is a constant, *f*: [0, 1] → *R* is a proposed window function and g: R → R is considered a linear function proposed earlier in the linear drift model (where *R* stands for real numbers). The authors demonstrated from the solutions that in order to imitate the working of the memristor proposed by Yang et al. [46], *g*(*v*) must be a non-linear, odd, and monotonically increasing function. A non-linear function which was proposed was [68]:

19 $$ g(v)={v}^q $$

Here, the exponent *q* is used to mimic the rapid switching process. Transition between ON and OFF state in a memristor generally takes place very fast. An input voltage with a very high sweep rate is used to obtain such behavior. This is the first implementation of memristor models in the SPICE platform [116, 117].The major advantage of SPICE implementation is the ability of the model to be used in analog circuits and simulations and can be verified as fit to be circuit implementable or not. Although many improvements were made in subsequent models, this model lays the foundation for the rest of the RRAM models by accurately taking into consideration and explaining the non-linear dopant drift effects [3, 46].

### Exponential Ion Drift Model

In practice, resistance switching characteristics are non-linear in nature. To analyze such exponential characteristics, Strukov et al. [69] proposed exponential ion drift model in 2009. This non-linearity caused a significant variation in retention time and write speed. Due to the exponential dependence of the switching rate for high electric field, the exponential ion drift model is generalized to explain the phenomenon by the non-linear microscopic drift of charged species in the dielectric at high field and temperature.

The major factors considered for this model are switching speed and volatility. Switching speed is the time required for the device to switch from one resistance state to the other, i.e., it can be deemed as the time required to writing the data into the memory and is denoted as τ_write_. Volatility is the time required for the device to lose its resistance state, i.e., the time taken to store the data into the device before erased denoted as τ_store_. The ratio between τ_store_ and τ_write_ derived using the Einstein-Nernst formula is given by [69]:

20 $$ {\tau}_{\mathrm{store}}/{\tau}_{\mathrm{write}}\sim EL\mu /D= qEL/{k}_BT $$

Here, *L* is the length of the device with an active doped region *D* and *k*_*B*_ the Boltzmann constant. Ratio between the two parameters is approximately three orders of magnitude when considered at room temperature and reasonable bias voltages. Such a high volatility to switching speed ratio suggests a strong non-linear ionic transport due to drift-diffusion inside the device. For high-field ionic drift, the overall effect on the average drift velocity of the ions is given by the model as [69]:

$$ \nu \approx {f}_e{a}_p{e}^{-\frac{E_a}{k_BT}}\sinh \left( qE{a}_p/2{k}_BT\right) $$

21 $$ \nu =\left\{\begin{array}{c}-\mu E,\kern0.5em E\ll {E}_0\\ {}\mu {E}_0{e}^{E/{E}_0},\kern0.5em E\sim {E}_0\end{array}\right. $$

Here, *ν* is the drift velocity, *f*_e_ the frequency of escape attempts, *T* the device temperature, *a*_p_ the periodicity, *E*_a_ the activation energy, and *E* the applied electric field.

**Fig. 5:**
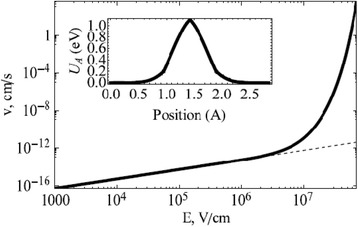
Nonlinear (solid) and linear (dashed) drift velocity of doubly charged oxygen vacancies along the [110] plane direction in rutile structure at room temperature [69]

Variation of the drift velocity with the applied electric field is shown in Fig. 5 [69]. The exponential variation can be clearly seen at high applied fields which lend non-linearity to the model. There are a few shortcomings for this model which affect its accuracy and also the calculation of the average drift velocity mentioned in Eq. (20). This model is primarily suited for application to ionic crystals where the major interaction forces are the Coulomb repulsion and van-der-Waals forces. Its application for covalent crystals will affect the accuracy of calculation due to the complex interactions of electrons and ions in high electric field. Also, electrochemical diffusion reactions and redox reactions are not explained by the model [91–93]. This can cause significant issues in the systems where the physical switching mechanism is governed by electrochemical processes.

### Simmons Tunneling Barrier Model

Though Lehtonen and Laiho [68] first proposed SPICE-based simulations model for non-linear ion drift model as mentioned in the “Non-linear Ion Drift Model” section, but this modeling is not suitable for use in an electrical-based time domain simulation, due to the lack of proper definition of simulation parameters and equations. This situation changed with the Pickett-Adballa et al. [70–72] model where a new class of model based on the device physics was demonstrated, which is capable of being explained and compatible with SPICE. The equations were modified to fit the requirements for SPICE implementation.

**Fig. 6:**
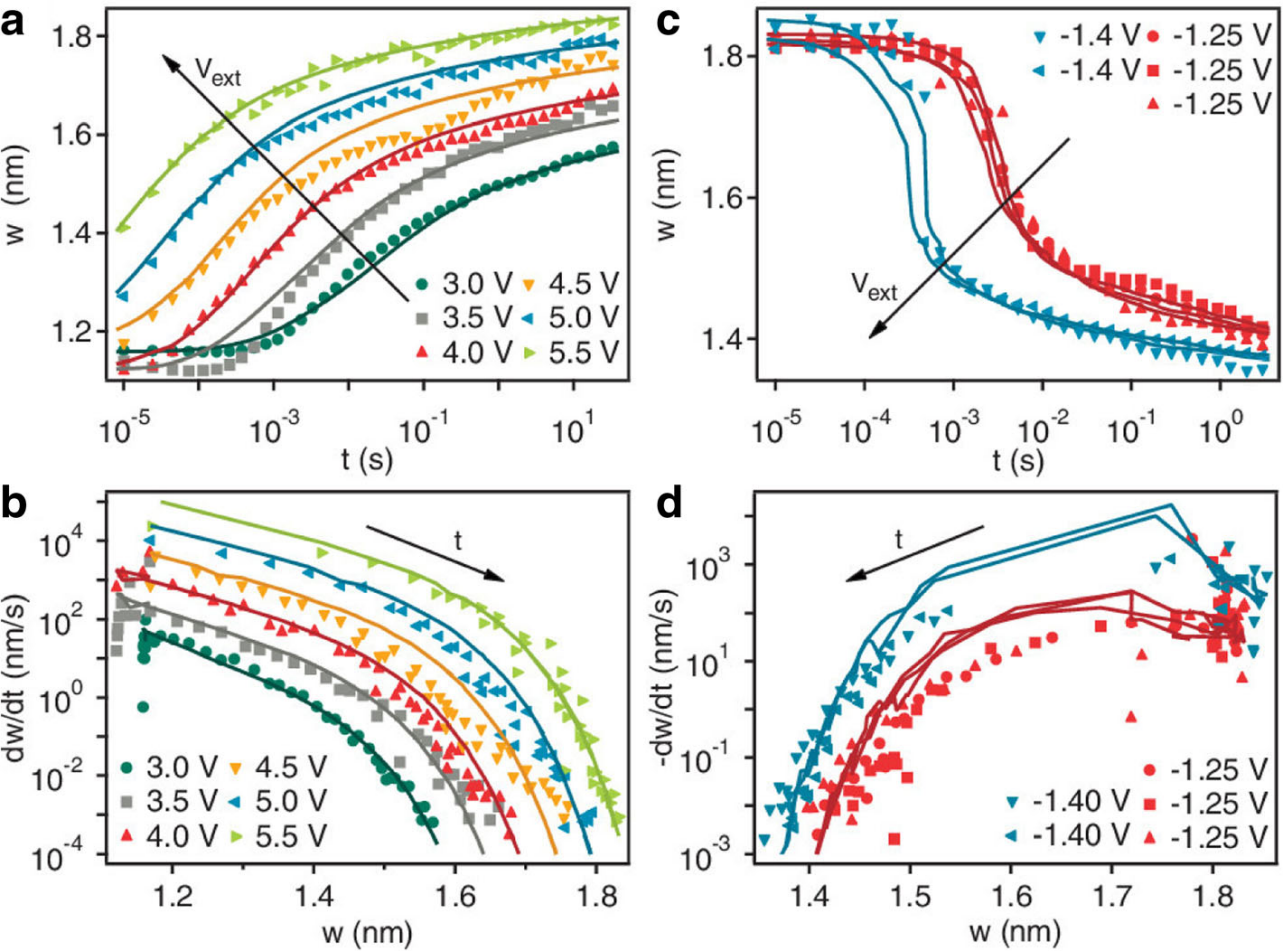
Dynamical behavior of the tunnel barrier width *w*. The evolution of the state variable *w* occurs as a function of time for different applied voltages for a series of **a** off-switching and **c** on-switching state tests on the same device. Legends indicate the applied external voltage. The lines are the numerical solution to the respective switching differential equations described in the text. **b**, **d** The numerical derivative *w*˙ of the data in **a** and **c** plotted as a function of *w* for the different applied voltages. The lines are calculated from the differential equations using the measured values of *w* and *i* at each point in time. The irregularity of the calculated *w˙* vs *w* lines in the on-switching plots is caused by the changes in the current that accompany the change in state (*w˙* is a function of two variables, *w* and *i*, and both are changing). The derivative of the state variable *w˙* can be interpreted as the speed of the oxygen vacancy front. This is because the applied voltage pushes it away from or attracts it toward the top electrode [70]

The analysis was based on the results from a TiO_2_-based memristor device [70] where the tunneling barrier width *w* was considered to be the dynamic state variable. This later set the precedent for one of the most popular parameters being treated as the dynamic variable in memristor systems, the other being the length of conductive filament inside the dielectric media. The deduction based on their analysis was that the dynamic behavior for on and off switching of the devices was highly non-linear and asymmetric as can be seen in Fig. 6 [70]. The explanation provided for the deduction was the exponential dependence of the drift velocity of ionized dopants on the applied current or voltage.

The current in the device was explained based on the Simmons tunneling barrier I-V expressions [137], and based on this analysis, the dynamic state variable was determined to be the Simmons tunnel barrier width (*w*). The current was given as [72]:

22 $$ i=\frac{j_0A}{\Delta  {w}^2}\left\{{\phi}_b{e}^{-B\sqrt{\phi_b}}-\left({\phi}_b+e\left|v\right|\right){\mathrm{e}}^{-B\sqrt{\phi_b+e\left|v\right|}}\right\} $$

where

23 $$ {j}_0=\frac{e}{2\pi h},{w}_1=\frac{1.2\lambda w}{\phi_0},\Delta  w={w}_2-{w}_1 $$

24 $$ {\phi}_I={\phi}_0-\left|{v}_g\right|\left(\frac{w_1+{w}_2}{w}\right)-\left(\frac{1.15\lambda w}{\Delta  w}\right)\ln \left(\frac{w_2\left(w-{w}_1\right)}{w_1\left(w-{w}_2\right)}\right) $$

25 $$ B=\frac{4\pi \Delta  w\times {10}^{-9}\sqrt{2 me}}{h} $$

26 $$ {w}_2={w}_1+w\left(1-\frac{9.2\lambda }{\left(3{\phi}_0+4\lambda -2|{v}_g|\right)}\right) $$

27 $$ \lambda =\frac{e.\mathit{\ln}(2)}{8\pi \varepsilon {\varepsilon}_0w\times {10}^{-9}} $$

The parameters have been adjusted here such that the barrier height *φ*_*b*_ is in volts (not in electron volts), and the time-varying tunnel barrier width *w* is in nanometers. In the equations above, *A* is the channel area of the memristor, *e* is the electron charge, *h* is the Planck’s constant, *ε* is the dielectric constant, *m* is the mass of electron, *φ*_0_ is a standard barrier height taken from reference [70], and *v* is the voltage across the tunnel barrier. *B* is a fitting constant. In lieu of the analytical form of the equations, they can be conveniently described and implemented in SPICE, or it can be implemented with the any SPICE compatible electrical simulator.

The dynamic state variable *w* varies with time as [72]:

28 $$ \frac{dw}{dt}={f}_1\sinh \left(\left(\frac{\mid i\mid }{i_1}\right)\exp \Big(-\exp \left(\frac{w-{a}_1}{w_c}-\frac{\mid i\mid }{b}\right)-\frac{w}{w_c}\right) $$

This is in the case of *off switching state* (*i >* 0). Whereas for *on switching state* (*i <* 0), the state variable varies as [72]:

29 $$ \frac{dw}{dt}=-{f}_2\sinh \left(\left(\frac{\mid i\mid }{i_2}\right)\exp \Big(-\exp \left(\frac{a_2-w}{w_c}-\frac{\mid i\mid }{b}\right)-\frac{w}{w_c}\right) $$

Here, *f*_1,_*i*_1_, *a*_1_, *b*, *w*_c_, *f*_2_, *i*_2_, and *a*_2_ are fitting parameters. The abovementioned equations are used to model the memristor on the circuit level considering the electron tunnel barrier as a voltage-dependent current source, and the conducting channel (TiO_2_) is modeled as a series resistance. The voltage drops across the tunnel barrier and the series resistance make up the complete voltage drop across the circuit.

The dynamic behavior of the device is visibly complex as it is physics-based modeling approach and has been articulated as such by the Eqs. (27) and (28). The rate of switching possibly has contributions from the nonlinear drift at high electric fields and local Joule heating of the junction speeding up the thermally activated drift of oxygen vacancies [16, 46, 82, 83]. This can be clearly seen in the case of Fig. 6a, c [70] where the nature of the curves at high electric fields is quite different to those in low fields. The switching in the device is directly affected by the width of the gap. Application of a positive bias on the top electrode increases the state variable *w* resulting in an exponential increase in the resistance of the device as illustrated in Fig. 6b, d [70]. An opposite phenomenon occurs when negative bias is applied on the top electrode. This signifies the bipolar nature of the switching characteristics and their dependence on the dynamic state variable *w*.

**Fig. 7:**
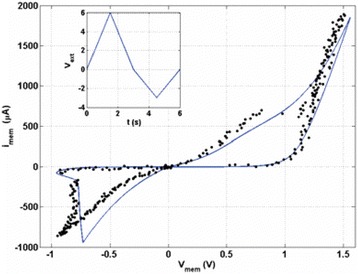
Experimental data (black dots) and corresponding simulated *I*-*V* curve for the memristor (solid line) where *i*_mem_ is the current through the memristor and *v*_mem_ is the voltage across the entire memristor. The inset shows the externally applied voltage sweep is shown and the initial condition for *w* is set at 1.2 nm [72]

The SPICE simulation of the model equations is illustrated in Fig. 7 [72]. The experimental data from the fabricated device is plotted against the simulated *I-V* curves showing a good fit between the two. This implementation paves the way for future SPICE simulations of RRAM devices [74, 77, 81]. A possible shortcoming in this model is the lack of a boundary for the dynamic variable and a threshold voltage within which the model should work. The growth of tunneling barrier width *w* can possibly go to unlimited quantities owing to the lack of a bound for the same, thus creating non-realizable scenarios for the device mechanism. Many models have employed what is called a window function to define the limits for the defined dynamic state variable in the model.

### Yakopcic Model

Although not validated specifically for RRAM devices at the time of development, the Yakopcic model [73, 74] closely resembled a variety of RRAM devices. The model was initially tested for TiO_2_ systems [73], and these systems are indeed one of the most popular ones along with HfO2-based RRAM devices.

This model was based on the Pickett-Adballa model [70–72] using a similar state variable, but it was modified to include neuromorphic systems as well. It was one of the first models to consider the functioning of synapses into their equations. This model was verified for the device used by the HP lab team to explain the working of memristive systems.

The state variable *w*(*t*), a value between zero and one considered here, directly affected the current through the device and also the dynamics of the device, i.e., the resistance. The current in the device is given as [73]:

30 $$ I(t)=\left\{\begin{array}{c}{a}_1w(t)\sinh \left( bv(t)\right),\kern2.25em v(t)\ge 0\\ {}{a}_2w(t)\sinh \left( bv(t)\right),\kern2.25em v(t)<0\end{array}\right. $$

Two functions, namely *g*(*v*(*t*)) and *f*(*x*(*t*)), are responsible for the change in the state variable. *a*_1_, *a*_2_, and *b* are fitting constants. Change of the state of the variable is generally governed by a threshold voltage, i.e., there is a physical change in the device structure above a certain threshold voltage. The function *g*(*v*(*t*)) here models the ON and OFF voltages of the device which also takes into account the polarity of the input voltage. This results in a better fit to the experimental data in case of bipolar switching where the values of set (*v*_*p*_) and reset (*v*_*n*_) voltage, i.e., the thresholds are different. It is defined as [73]:

31 $$ g\left(v(t)\right)=\left\{\begin{array}{c}{A}_p\left({e}^{v(t)}-{e}^{v_p}\right),\kern0.5em v(t)>{v}_p\\ {}-{A}_n\left({e}^{-v(t)}-{e}^{v_n}\right),\kern0.5em v(t)<-{v}_n\\ {}\kern2.75em 0,\kern3em -{v}_n\le v(t)\le {v}_p\end{array}\right. $$

*A*_*p*_ and *A*_*n*_ indicate the rate of the change of state once the voltage threshold is crossed. It can be understood as the dissolution or the rupture of the filament in terms of RRAM devices. There is in-built support for threshold values in the model, which enhances its applicability.

The state change variable modeled by the function *f*(*w*(*t*)) is used to define the boundaries for the variable. It explains the motion of the charge carrying particles based on the threshold values, also adding the possibility to define the motion of the particles based on the polarity of the input voltage. This basically acts as a window function which restricts the state change variable within certain boundary given as [73]:

32 $$ f(w)=\left\{\begin{array}{c}{e}^{-{\alpha}_p\left(w-{w}_p\right)}{f}_p\left(w,{w}_p\right),\kern0.5em w\ge {w}_p\\ {}1,\kern10em w<{w}_p\end{array}\right. $$

33 $$ f(w)=\left\{\begin{array}{c}{e}^{\alpha_n\left(w+{w}_n-1\right)}{f}_n\left(w,{w}_n\right),\kern0.5em w\le 1-{w}_n\\ {}1,\kern10.5em w>1-{w}_n\end{array}\right. $$

Here, *f*_*p*_(*w*,*w*_*p*_) is a window function which limits the value of *f*(*w*) to 0 when *x*(*t*) = 1 and *v*(*t*) > 0. *f*_*n*_(*w*,*w*_*n*_) is a similar window function which does not allow the value of *w*(*t*) to become less than zero when the current flow is reversed.

The window functions are defined as [73]:

34 $$ {f}_p\left(w,{w}_p\right)=\frac{w_p-w}{1-{w}_p}+1 $$

35 $$ {f}_n\left(w,{w}_n\right)=\frac{w}{1-{w}_n} $$

The movement of dynamic state variable, in simple words, the rate of switching, is governed by a differential equation. The growth and decay of the tunneling barrier width are the defining mechanism for this particular model, and it is given by [73]:

36 $$ \frac{dw}{\mathrm{d}t}=g\left(v(t)\right)f\left(w(t)\right) $$

Owing to the analytical nature of the coupled equations, they can be solved using a mathematical solver such as MATLAB [138, 139]. The differential equation can also be solved in MATLAB using the in-built solvers *idt*() and *ddt*() functions, which employ the time step integration method. This particular model was simulated using the characterization data of the TiO_2_ memristor from HP Labs [3], and the fitting obtained was pretty good when the fitting parameters are properly calibrated.

A separate SPICE implementation of the same model was reported by Yakopcic et al. [74] which were fitted and characterized for a multitude of devices for both sinusoidal and repeated sweep inputs. The SPICE implementation revealed a good accuracy and applicability of the model at the circuit level. The model was correlated with a variety of experimental data, and low error rates of about 6% were obtained. It was one of the first SPICE implementation where the model was tested under sinusoidal as well as repetitive sweeping inputs. This helps in determining the AC behavior of the device. Along with that, very important device variability analysis is performed which defines the error tolerance in the device. Variability is an important issue, when the RRAM device is used in large systems, such as arrays. The variability analysis performed is essential in knowing until which point the system can tolerate the variability. After reaching the critical point, there is possibility of errors in device read/write.

The model was also tested for read/write operations using 256 devices, which helps determine its usability in crossbar arrays. Similarly, it can be used for neuromorphic read/write operations to test the model applicability in that system. Device variability in the model is defined with change in the device parameters. So, changing the device parameters leads to a change in the simulated device *I*-*V* which is very useful in fitting the model with the experimental data. The values of the device parameters used can help define the accepted values of the particular parameters in the real case scenario. No convergence errors were found in the 256 array system, but with new RRAM array systems reaching higher density, applicability of the model there remains a question. Higher density array systems generally pose a convergence problem in SPICE simulations, but with proper parameter definition, it can be avoided. This model can be considered a new paradigm when it comes to circuit level SPICE simulations, variability analysis, and read/write operation simulations for RRAM devices.

### TEAM/VTEAM Model

Threshold Adaptive Memristor (TEAM) model [75, 76] builds based on the Simmons Tunneling Barrier model [70–72] (discussed in the “Simmons Tunneling Barrier Model” section) and delivers a much simpler physics-based modeling approach for memristive systems. *I*-*V* relationship in this case is not fixed and can be chosen to fit any device which provides some amount of flexibility in the model. TEAM model arose from the need of simpler analytical equations which describe the mechanism of memristive systems accurately and which take less computation time.

**Fig. 8:**
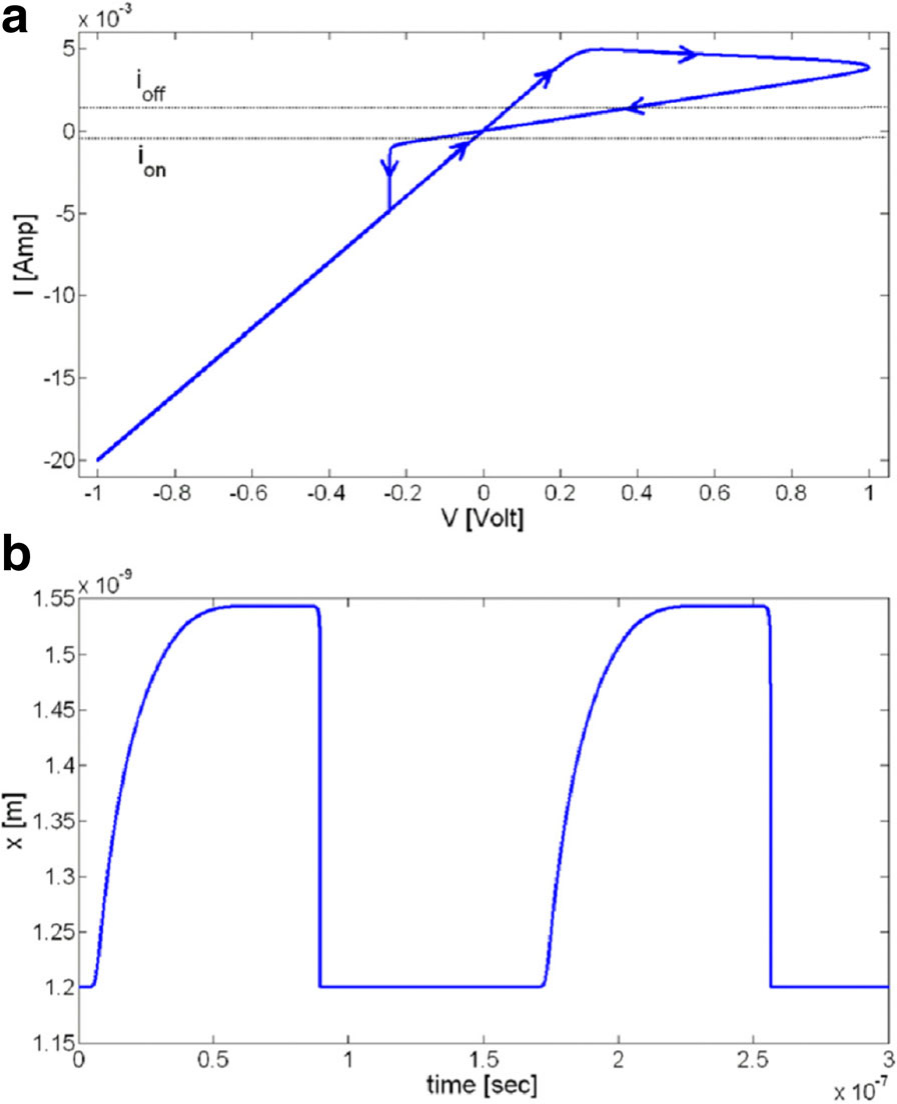
A sinusoidal input of 1 V applied to the TEAM model using the same fitting parameters as used in Fig. 10 [76]. The values of *R*_ON_and *R*_OFF_ are set as 50 Ω and 1 kΩ, and an ideal rectangular window function is applied in Eqs. (38) and (37). **a** *I*-*V* curve and **b** state variable. It is to be noted that the device is asymmetric, i.e., switching OFF is slower than switching ON [76]

This model is based on the approximation of the high non-linear dependence of the memristive device current; the device can be modeled as a device with threshold currents. The results are evident in Fig. 8. As with the tunneling barrier model, the internal state derivate is dependent on the current and the state variable itself, which is the effective tunnel width. It can be modeled effectively by [76]:

37 $$ \frac{dw(t)}{dt}=\left\{\begin{array}{c}{k}_{\mathrm{off}}\times {\left(\frac{i(t)}{i_{\mathrm{off}}}-1\right)}^{\alpha_{\mathrm{off}}}\times {f}_{\mathrm{off}}(w),\kern0.5em 0<{i}_{\mathrm{off}}<i\\ {}0,\kern12.75em {i}_{\mathrm{on}}<i<{i}_{\mathrm{off}}\\ {}{k}_{\mathrm{on}}\times {\left(\frac{i(t)}{i_{\mathrm{on}}}-1\right)}^{\alpha_{\mathrm{on}}}\times {f}_{\mathrm{on}}(w),\kern2.25em i<{i}_{\mathrm{on}}<0\end{array}\right. $$

Variation of the state variable with time is asymmetrical in nature, as shown in Fig. 8b. This means that the ON and OFF switching times are not equal. In the Eq. (36), *i*_on_ and *i*_off_ act as the current thresholds. Functions *f*_on_ and *f*_off_ are window functions which bound the internal state variable *x*(*t*) within [*w*_on_, *w*_off_]. Window functions are described as [76]:

38 $$ {f}_{\mathrm{off}}(w)=\exp \left[-\exp \left(\frac{w-{a}_{\mathrm{off}}}{w_c}\right)\right], $$

39 $$ {f}_{\mathrm{on}}(w)=\exp \left[-\exp \left(-\frac{w-{a}_{\mathrm{on}}}{w_c}\right)\right], $$

The window functions describe the dependence of the derivative in the state variable *x*. They work well within the described boundaries, but the problem arises when the device goes beyond the boundaries. There are no limiting parameters here, and the window function only describes the state variable inside a particular limit. If the device goes beyond the boundaries, it can cause convergence issues with the simulator and it does not make sense for good modeling practice in case of analog devices.

*I*-*V* relationship in this model is derived from the tunneling barrier model, as discussed in the “Simmons Tunneling Barrier Model” section. Due to the non-linear nature of the tunneling current, the change in resistance varies exponentially with the state variable. So, it is assumed that any change in the tunnel barrier width changes the memristance in an exponential manner which deduces to [76]:

40 $$ v(t)={R}_{\mathrm{ON}}{e}^{\left(\lambda /{w}_{\mathrm{off}}-{w}_{\mathrm{on}}\right)\left(w-{w}_{\mathrm{on}}\right)}\times i(t) $$

Here, λ is a fitting parameter and *R*_ON_ the equivalent effective resistance at the bounds.

*I*-*V* relationship for this model can be seen in Fig. 8a [76]. Although there is a presence of a pinched hysteresis, the form and structure of the curve are not well-defined. The model is driven with a sinusoidal input of 1 V. The verification done for this model is different from the tunneling model [70–72] in terms of the platform used to simulate it. The latter model uses a SPICE macro model [72] to describe the equations, but SPICE takes up a significant amount of computation time. Modeling in Verilog-A [140–143] is much more efficient, and the TEAM model [75] utilizes this functionality to model the equations presented by them.

A slightly modified version of the TEAM model with the introduction of voltage threshold levels was reported by the same group, called Voltage Threshold Adaptive Memristor model (VTEAM) [77]. Discussed TEAM model was based on threshold currents, whereas VTEAM is based on threshold voltages. The major advantages cited for using threshold voltages is that comparison with current causes performance and reliability issues if the condition is not satisfied, i.e., a low-current threshold will automatically have a low-voltage threshold as well. This might affect the overall performance of the device. Also with a threshold voltage, there is no risk with going overboard with high power and voltage destroying the device as the values are automatically controlled.

The VTEAM follows a similar concept to the TEAM model, being based on an expression of the derivative of an internal state variable. The current is dependent on the state variable itself. The only difference is inclusion of a threshold voltage. The internal state variable (*w*) is defined as [77]:

41 $$ \frac{dw}{dt}=\left\{\begin{array}{c}{k}_{\mathrm{off}}\times {\left(\frac{v(t)}{v_{\mathrm{off}}}-1\right)}^{\alpha_{\mathrm{off}}}\times {f}_{\mathrm{off}}(w),\kern0.5em 0<{v}_{\mathrm{off}}<v\\ {}0,\kern14.75em {v}_{\mathrm{on}}<v<{v}_{\mathrm{off}}\\ {}{k}_{\mathrm{on}}\times {\left(\frac{v(t)}{v_{\mathrm{on}}}-1\right)}^{\alpha_{\mathrm{on}}}\times {f}_{\mathrm{on}}(w),\kern2.5em v<{v}_{\mathrm{on}}<0\end{array}\right. $$

Similar to the TEAM model, the functions *f*_on_ and *f*_off_ act as window functions which bound the internal state variable *w* within [*w*_on_*, w*_off_]. As has been assumed in the model, current varies exponentially with the internal state variable on most occasions which is defined by [77]:

42 $$ i(t)=\frac{e^{-\frac{\lambda }{w_{\mathrm{off}}-{w}_{\mathrm{on}}}\times \left(w-{w}_{\mathrm{on}}\right)}}{R_{ON}}\times v(t) $$

**Fig. 9:**
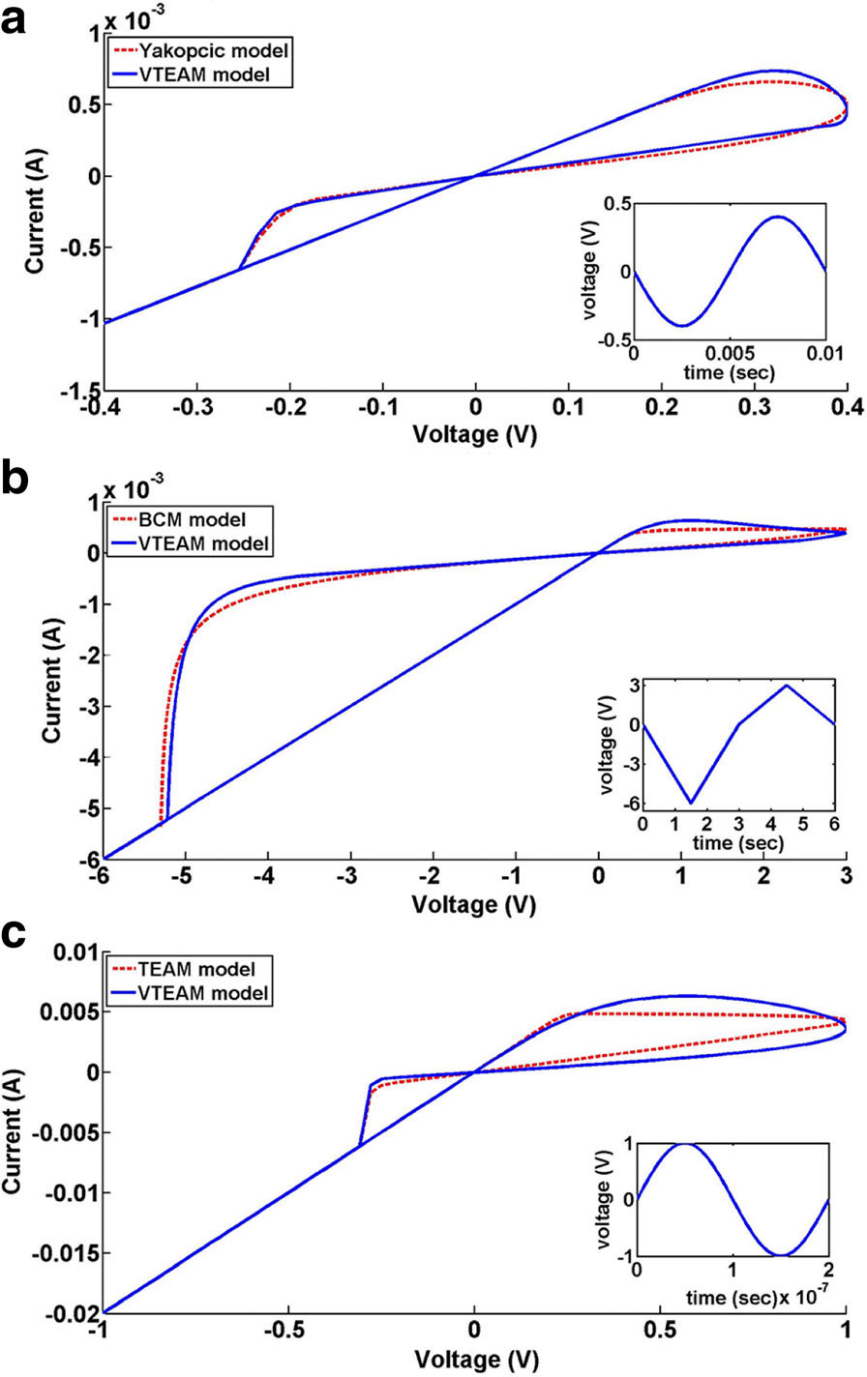
The VTEAM model is compared with previously proposed memristor models [77]. **a** Yakopcic model [73]. **b** BCM model [99]. **c** TEAM model [76]

The comparative analysis of the VTEAM model with the Yakopcic model [73, 74], BCM model [99] (discussed further in this article), and the TEAM model are presented in Fig. 9 [77]. It represents the flexibility that the model possesses, as it can be tuned to fit all the three models. It shows good agreement with all the three models illustrated, respectively, in Fig. 9a–c [77]. Fundamentally, the TEAM/VTEAM models are quite generalized physics-based models. This means that with the help of fitting parameters, they can be comparable with the multitude of other models, and fit to a variety of experimental characterization data from memristive systems.

### Stanford/ASU Model

A physics-based model which has become very popular is the one developed by Guan et al. and Chen et al. of Stanford University and ASU, known as Stanford/ASU model [78–80]. This model is exclusively developed for RRAM devices, rather than a generalized one for memristive systems which was fitted for those particular devices. It included the effect of critical phenomenon of switching such as Joule heating and temperature change, which had been neglected before. The developed model was applied in the *I*-*V* switching characteristics of HfO_2_RRAM [144]. Along with it, Verilog-A [79] and SPICE [81] implementations of the model are also presented.

This model is based on the growth of conductive filament. The CF growth leaves a gap with the top electrode which is called as the filament gap. This growth of the filament gap is considered as internal state variable in this case. So, the rate of filament growth and the filament gap govern the dynamics of the model. The filament growth is explained due to the movement of oxygen ions and vacancy regeneration and recombination [145]. Considering the gap value *g* (nominally in the range of 0–3 nm) to be the state variable, the rate of change of *g* is defined as [78]:

43 $$ \frac{dg}{dt}={\nu}_0\exp \left(\frac{-{E}_{a,m}}{k_bT}\right)\sinh \left(\frac{q{a}_h\gamma v}{L{k}_bT}\right) $$

The parameter *E*_*a*_ is the activation energy for vacancy generation and oxygen vacancy migration in the SET and RESET processes, respectively. *v* is the applied voltage across the device, *ν*_0_ the velocity containing the attempt-to-escape frequency, *L* the switching material thickness and *a*_*h*_, the hopping site distance.

A significant feature of this model is the inclusion of variations in the model caused due to the stochastic property of the ion process and the spatial variation in the gap size among multiple filaments. To account for these variations in the model, a noise signal is added to the gap distance as [78]:

44 $$ g\mid t+\Delta  t=F\left[g|t,\frac{dg}{dt}\right]+{\delta}_g\times \overset{\sim }{X}(n)\Delta  t,\kern2.25em n=\left\lfloor \frac{t}{T_{GN}}\right\rfloor $$

The variation in the gap size *δ*_*g*_ is defined as a function of the ions’ kinetic energy and invariably on the temperature in the filament and is given as [78]:

45 $$ {\delta}_g(T)=\frac{\delta_g^0}{\left\{1+\exp \left[\frac{\left({T}_{\mathrm{crit}}-T\right)}{T_{\mathrm{amb}}}\right]\right\}} $$

Here, *T*_crit_ is defined as a threshold temperature beyond which there is a significant change in the gap size. This can be understood as the point where the device undergoes a physical transformation such as transitioning into a SET or RESET state. In this case, threshold is considered in terms of temperature, rather than voltage or current, whatever employed in the previous models [75–77]. So, the equation basically depicts the resistance fluctuation that occurs when the CF temperature is increased beyond the room temperature.

Now that temperature can be considered a critical driving force in the model, a modified form of the steady-state Fourier heat flow equation is implemented in this model. Rather than considering heat flow throughout the filament, the vicinity of the tip of the filament is considered. There is a dynamic inner domain temperature *T* which significantly changes with change in the cell characteristics, and an outer domain remains at an ambient room temperature *T*_amb_, related as [78]:

46 $$ {c}_p\frac{\partial T}{\partial t}=v(t)i(t)-k\left(T-{T}_{\mathrm{amb}}\right) $$

*c*_*p*_ is the effective heat capacitance of the inner domain, and *k* the effective thermal conductivity are both fitted based on the type of oxide and electrodes used in the RRAM system. RESET transition from LRS to HRS generally has higher temperature associated with it across the device, while the SET transition has a considerably lower temperature. The current inside the device is modeled using a generalized conduction mechanism where the tunneling distance and field strength have an exponential relationship. This is true in case of tunneling current conduction mechanisms such as Poole-Frenkel, Fowler-Nordhiem, trap-assisted, or direct tunneling [9, 16, 46, 49, 51, 55]; these are the mechanisms most commonly associated with RRAM systems [51, 55, 61, 66]. The current conduction is defined as [78].

47 $$ i\left(g,v\right)={i}_0\exp \left(\frac{-g}{g_0}\right)\sinh \left(\frac{v}{v_0}\right) $$

**Fig. 10:**
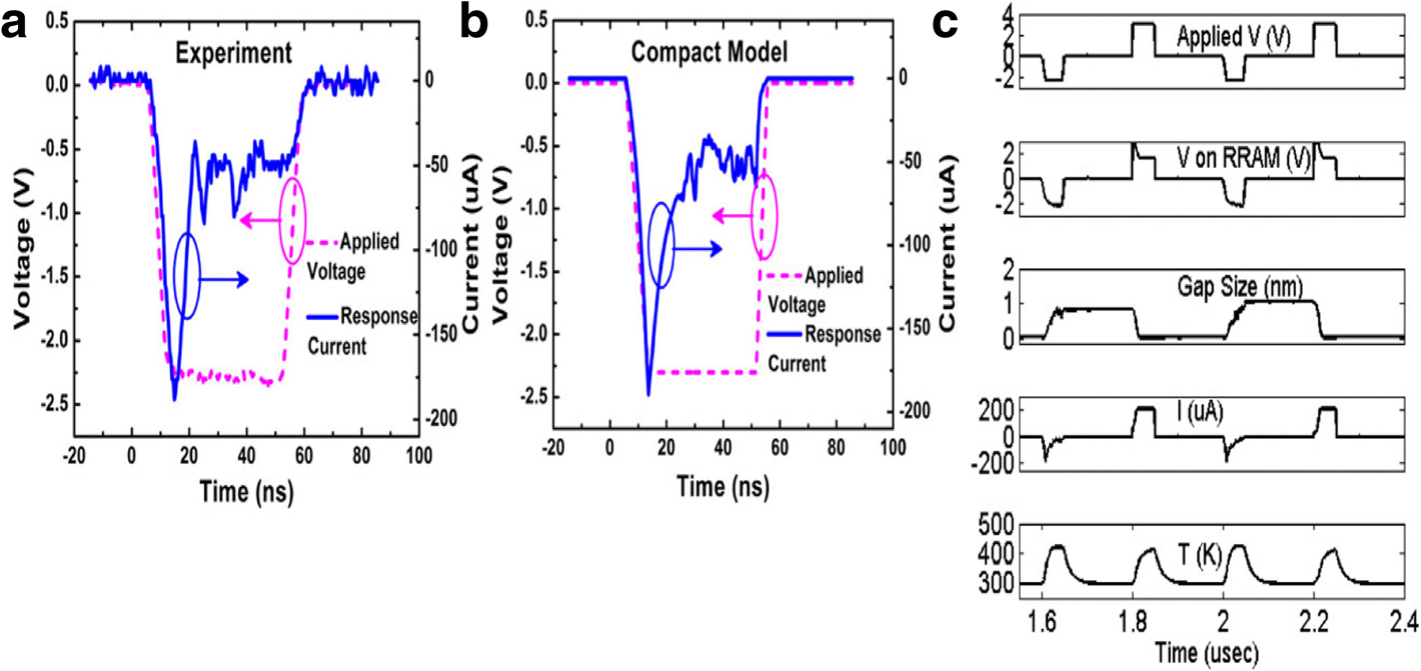
**a** Experimental and **b** simulated transient responses of a HfO_*x*_ RRAM device to the *−* 2.3 V 50 ns input pulses. The experimental result is reported elsewhere [144] and included here in **a** for convenience. **c** In a larger time range, the simulated transient response for the same device including the gap size and temperature is shown. Current compliance set at 200 *μ*A in simulation [78]

The advantage with a generalized current equation is that for a particular device if some other mechanism is fitting better, it can be incorporated easily by adding the required parameters and adjusting their values accordingly. *I*-*V* response of the model compared with experimental data is shown in Fig. 10. The experimental response is shown in Fig. 10a while the simulated curve is shown in Fig. 10b. Simulated transient response shows the capabilities of the model in taking variations into account. Developed model was verified using Ngspice [146] as a macrocircuit. Ngspice is an open source SPICE simulator which is quite efficient and convenient for doing DC and AC analysis. This model can be implemented in MLC memory circuits and also to verify the efficiency of programming strategies and error correction codes [78].

A major feature of this model is implemented in the neuromorphic systems and RRAM synaptic device design [147]. This model has been tested against a HfO_*x*_/TiO_*x*_ multi-stack RRAM system [148] which is implemented in a neuromorphic system. This gives the model great flexibility and wide applications as there are only a few models that are actually applicable for neuromorphic systems. Also, the model defined for these systems has been deemed tolerant to training error caused by device variation [149]. The gradual resistance modulation which is critical to the learning process in a synaptic device can be quantified in the model [150] which marks a significant development in using RRAM synaptic stacks in neuromorphic computing systems.

### Physical Electro-Thermal Model

This model is an extensive physical model which describes the bipolar operation in RRAM devices using equations closely resembling the physical mechanisms. This model was reported by Kim et al. [87], and it was verified with a tantalum pentoxide (Ta_2_O_5_)-based bi-layered RRAM structure [15, 151, 152]. It makes use of the finite element solving method employed in the previous model to solve the differential equations. The major value addition by this model over the model proposed by Larentis et al. [86] was the proper description provided for the SET state in the bipolar RRAM device. The previous model was inadequate in accommodating the complete transition and explaining it properly but this model makes up for that. Also, it improved upon a physical electro-thermal model reported by Menzel et al. [153] which attempts at calculating the CF temperature precisely.

It also uses the electro-thermal physics phenomenon approach for modeling which we have seen in the previous model [86]. The major advantage with models based on this concept is their ease of use owing to the simple fundamental equations and the flexibility to employ a proper finite element method (FEM) solver to simulate the system very accurately. But a major disadvantage is that the model becomes very difficult to implement in circuit solvers based on SPICE and providing an equivalent implementation in Verilog. This is because of the lack of support in SPICE and Verilog for properly defining partial differential equations which make up for the vastness of the model. Normal ordinary differential equations and the ones which are in analytical form can be solved in circuit solvers but partial differential equations (PDE) cannot be solved.

Electro-thermal models are equally important as compared to the other physics-based models discussed before because temperature is an important factor governing the set and reset processes. Ion and vacancy migration plays a dominant role for switching mechanism [16, 46], although the governing factors are behind this process and the exact type of ions is still up for debate. So, the fact that temperature is a governing factor in this process makes these models attention worthy. Also, experiments [85, 154] in this regard suggest that there is significant change in the temperature in the CF during the switching process. Some of the previous models discussed above have neglected this effect by considering conducting filament-oxide interface to be at room temperature or by taking constant conducting filament temperature [39, 86, 88, 89, 144].

The major difference between this model and the previously discussed electro-thermal model is in the expressions used to describe the drift-diffusion process. CF is described as a doped region where the oxygen vacancies act as dopants, and the CF runs from the top electrode to the bottom electrode. This is an assumption that many models take that the CF runs from one end of the electrode to the other when the state variable is considered as the length of CF. A few models discussed previously [78, 80] have used the filament gap to the top electrode as state variable. So, the assumptions generally vary from system to system and are dependent on what mechanism is employed to describe the device.

Another assumption taken to describe the drift-diffusion of vacancy migration is that the same equation used can describe both the oxygen ions and vacancies. This is generally the case to simplify the model and reduce the complexity of the equations. The rigid point ion model by Mott and Gurney [155] is employed here to describe the process given as [87].

48 $$ \frac{\partial {n}_D}{\partial t}=\nabla \times \left({D}_s\nabla {n}_D-\mu v{n}_D\right)+G $$

where *D*_*s*_ describes the diffusion process, *v* gives the drift velocity of the vacancies, and *G* is the generation rate of vacancy or the CF growth rate which actually describes the SET process. The *G* term is a specialized parameter added to better describe the complete switching process [156, 157]. The parameters are defined as [87]:

49 $$ {D}_s=\frac{1}{2}\times {a}^2\times {f}_e\times \exp \left(-{E}_a/{k}_{\boldsymbol{B}}T\right) $$

50 $$ v={a}_h\times f\times \exp \left(-{E}_a/{k}_BT\right)\times \sinh \left(q{a}_hE/{k}_BT\right) $$

51 $$ G=A\times \exp \left(-\left({E}_a-q{l}_mE\right)/{k}_BT\right) $$

Here, *l*_m_ is the mesh size. So, using the Eqs. (48)–(50), the oxygen vacancy transport given in Eq. (47) can be defined which contains all the factors of drift-diffusion as well as the vacancy regeneration. These equations govern the CF growth and rupture which defines the physical transformation of the device during the SET and RESET transition of the device. So, it basically acts as a dynamic internal state variable which controls the switching rate of the device.

**Fig. 11:**
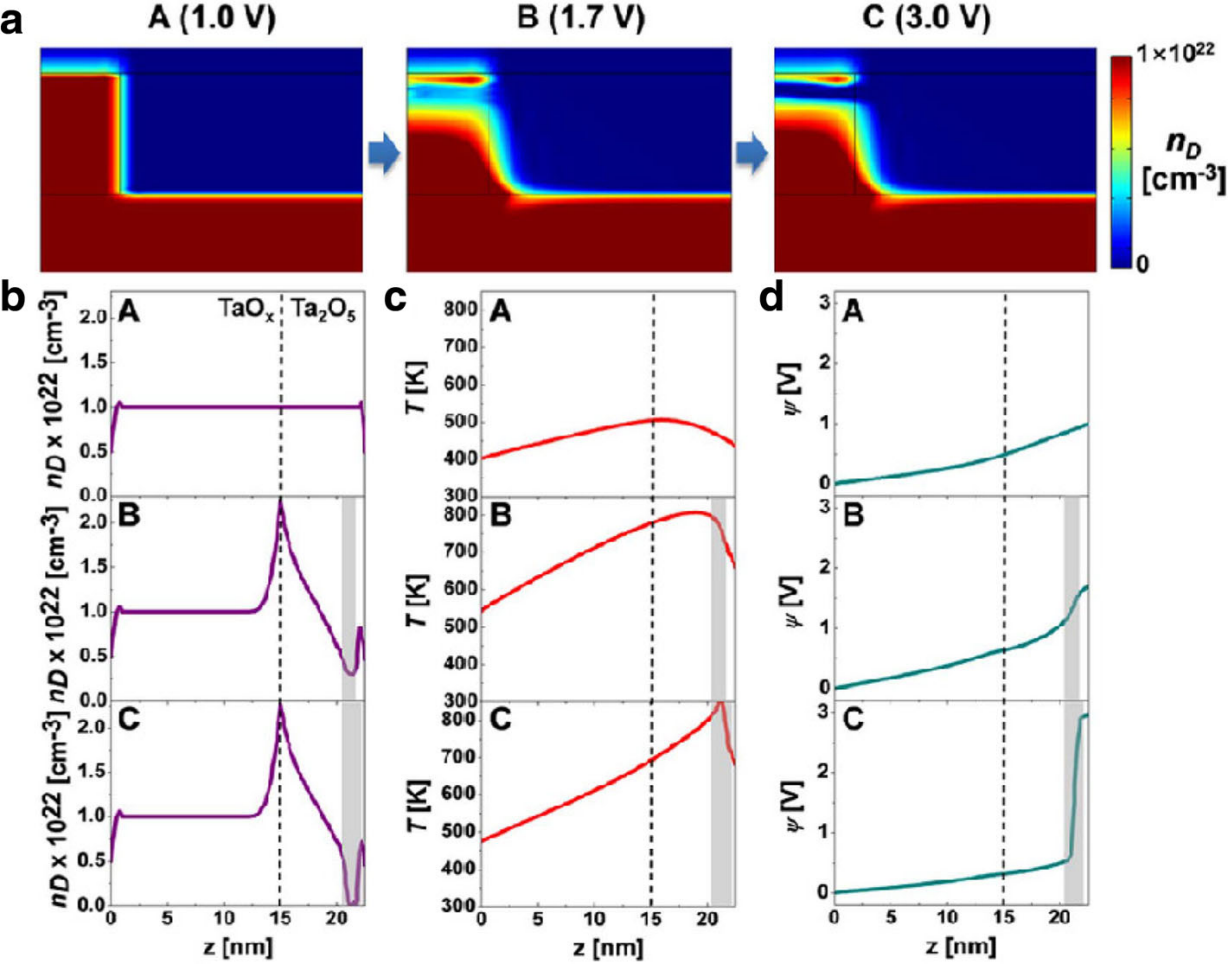
Simulation results for the reset transition of the device. **a** *V*_o_ density (*n*_D_) map. Calculated profiles of **b** *n*_D_, **c** *T*, and **d** *y* for states A (1.0 V), B (1.7 V), and C (3.0 V). The position of *z* = 15 nm indicates the Ta_2_O_5_/TaO_*x*_ interface in the structure schematic. The shaded area shows the depleted gap, defined for *n*_D_ < 5 × 10^21^ cm^-3^ [87]

The simulation results for the *reset* transition is shown in Fig. 11 [87]. Concentration of the oxygen ions is shown at different voltages in Fig. 11a [87] which invariably governs the switching in the device. The point C (3.0 V) is the point where the reset transition occurs, so the concentration of ions is also the highest at the interfaces for that voltage point as evident in Fig. 11b [87]. On similar lines, the temperature and flux are on the higher side which can be seen in Fig. 11c, d, respectively [87].

Equations (95) and (98)mentioned further are also used in the model to describe the current conduction and the temperature change due to Joule heating in the device. The equations are simultaneously solved in COMSOL to generate the required simulated profiles. The obtained simulated profiles are compared and verified against a TaO_x_ bi-layered RRAM system [87]. In addition to the DC *I*-*V* characteristics the model was also used to generate time-dependent reset characteristics by investigating its response to square pulses.

### Huang’s Physical Model

A very comprehensive physical model of RRAM devices is developed by Huang et al. [88, 89]. Its major feature is its consideration of the multitude of factors affecting the CF dynamics in the RRAM device. This model is comprehensive in the sense that it considers both the width of CF as well as filament gap to the electrode as factors affecting the state variable dynamics. The model was validated in a TiO_2_ based device and also applied in a 2 × 2 RRAM array cell [88].

Covering bipolar devices primarily, it also accounts for the temperature distribution in the device with multiple heating sources. SET/RESET process is considered to be caused due to generation/recombination process of the oxygen ions (O^2−^) and oxygen vacancies (*V*_o_). Top electrode (TE) is the active electrode and acts as an oxygen reservoir for the release or absorption of oxygen ions [88]. The CF evolution during the SET process is modeled based on the width of the CF. Growth of the CF is thought to start from the tip of the active electrode. With an increase of voltage the CF enlarges along the radius resulting in a final width of the CF as *w*. So, the value of w is critical to determine the LRS resistance in the SET process. Huang et al. [88] assumed that the CF grows in a symmetrical cylindrical shape which is simplifying at best. While the cylinder has been the most popular to describe the shape of the CF, it might not be the most accurate.

Rupture of the CF during the reset process is considered to start from the TE first. CF disconnects from the starting point and then dissolves internally with increase in the voltage. Distance between the tip of the CF and the active electrode layer is defined as the filament gap distance (*x*). The value of *x* determines the resistance of HRS during the RESET process. *x* and *dx/dt* are thus critical in defining the RESET process. A very important feature of the model is that there are two parameters defining the state of the system, in place of one parameter. The parameter *w* acts as the state variable for the SET process and *x* for the RESET process. So *dx/dt* and *dw/dt* define the dynamics of the device during the SET/RESET transition. Analytical model for a RRAM cell presented by Huang et al. [88] is developed by modeling the parameters x, w and their evolving speeds.

This model also presents one of the most detailed descriptions for the processes involved behind the RESET process. The rate of the CF shortening is affected by three processes, (a) O^2−^ release by the electrode, (b) O^2−^ hopping in the oxide layer, and (c) recombination between O^2−^ and *V*_o_. Slowest process among the three dominates the CF reduction process which is defined by the parameter *x*. Speed of the processes is affected by the specific device characteristics and the oxide used.

CF reduction rate during first reset process, i.e., O^2−^ release by the electrode can be given as [89]:

52 $$ \frac{dx}{dt}=a\times f\times \exp \left(-\frac{E_i-\gamma ZeV}{k_BT}\right) $$

In case of the O^2−^ hopping in the oxide layer, the CF with *a* being the distance between two V_o_, reduction rate is described by [89]:

53 $$ \frac{dx}{dt}=a\times f\times \exp \left(-\frac{E_h}{k_BT}\right)\sinh \left(\frac{a_h ZeE}{k_BT}\right) $$

The RESET process when dominated by the recombination between O^2−^ and V_o_ is written as [89]:

54 $$ \frac{dx}{dt}=a\times f\times \exp \left(-\frac{\Delta  {E}_r}{k_BT}\right) $$

The value of *x* is fixed to *x*_0_ after the RESET process. This invariably will act as the boundary condition for the model. But the problem here is the value and the role of *x*_0_ is not clearly defined here. This will possibly create ambiguities while defining the states of the device or switching between two states. In the first step of the SET process which is dominated by recombination of oxygen vacancies and where a thin CF is initially grown is described by [89]:

55 $$ \frac{dx}{dt}=-a\times {f}_e\times \exp \left(-\frac{E_a-{\alpha}_a ZeE}{k_BT}\right) $$

Here, Z and α_a_ are fitting parameters. In the second step, the CF grows along the radial direction of the CF is defined as [89]:

56 $$ \frac{dw}{dt}=\left(\Delta  w+\frac{\Delta  {w}^2}{2w}\right)\times {f}_e\times \exp \left(-\frac{E_a-\gamma Zev}{k_BT}\right) $$

Current flowing through the device has been taken in the model due to the hopping conduction and metallic conduction. The current in CF region can be calculated using the basic structures of Ohm’s law and Arrhenius law [158]. But the current in the gap region as a result of hopping conduction is given a little different. It is modeled as a correlation of the hopping current with the voltage and gap distance is given by [147]:

57 $$ i={i}_0\exp \left(-x/{x}_T\right)\sinh \left(v/{v}_T\right) $$

Temperature effects in the model are considered from the Filament Dissolution model [82, 83] discussed further in the “Filament Dissolution Model” section. Validation of the model is performed in HfO_x_/TiO_x_ system [88, 89]. Transient results obtained from simulating the model are compared against the data from the device, which shows a good match as demonstrated by Huang et al. [88]. The model is also validated against devices fabricated by other groups [144, 159] and the parameters are adjusted accordingly. A pretty accurate match between the simulation and the experimental results suggests a good level of flexibility with the model. The model also demonstrates that the switching speed of the device is highly dependent on the input voltage sweep rate.

Although the model is very comprehensive and takes into account a variety of detailed processes affecting the RRAM operation; it has some critical shortcomings. A major one is the non-compatibility with SPICE or Verilog-A. Implementations in any of the circuit simulators based on these platforms has not been demonstrated which raises a question on its readiness for simulations. Also, boundary conditions and non-linear effects have not been applied in the model which leaves it open to unphysical solutions. There has been no attempt to fit a window function with the model to account for this effect. These shortcomings make the model difficult for application for simulations, but its physics give a lot of insights into the functioning of RRAM devices.

### Bocquet Bipolar Model

A very interesting and unique model from Bocquet et al. [90, 92] which utilizes a physics based modeling approach to describe bipolar oxide based resistive switching memories. This was a model developed exclusively for the RRAM devices. Although a point of speculation still exists, it has been more or less accepted that the bipolar resistive switching mechanism is governed by the valence change mechanism which occurs in specific transition metal oxides and the field-assisted motion of oxygen ions O^2−^ [160].

This is also one of the few models that can describe electroforming process. This process basically initiates the CF growth for the first time when the device is in a pristine state. It requires significantly higher voltage as compared to the *set* or *reset* voltage because the CF formation requires an electric breakdown of the oxide and this requires higher voltage and energy. However, forming free RRAM devices have been reported [85] by adjusting the oxygen stoichiometry of the active layer. Removal of the forming process will reduce the voltage requirement of the device and make it more energy efficient.

Bocquet bipolar model uses some concepts from the Bocquet unipolar model [90] and modifies it significantly according to the bipolar switching characteristics. Major features of the model are its intrinsic simplicity in the model equations, full compatibility with SPICE based electric simulators and inclusion of voltage and time dependencies of the device. Internal state variable here is the radius of the CF which governs the switching rate. Radius of the CF varies with growth/rupture mechanism of the CF which is explained in the model with the help of local electrochemical redox processes [82, 83, 105, 161] which are dependent on the applied bias polarity. A single master equation in which both the SET and RESET processes are accounted for simultaneously is controlled by the CF radius which thus gives the switching rate of the device.

Electroforming stage is modeled using electroforming rate which describes the process of conversion of the pristine oxide into a switchable sub-oxide layer. CF radius (*r*_CF_) varies from a minimum value of 0 to a maximum value of *r*_CFmax_. The electroforming stage is modeled as [92]:

58 $$ {\tau}_{\mathrm{form}}={\tau}_{\mathrm{form}0}\times {e}^{\frac{E_{a\mathrm{Form}}-q\times {\alpha}_s\times {v}_{\mathrm{Cell}}}{k_b\times T}} $$

59 $$ \frac{d{r}_{\mathrm{CFmax}}}{dx}=\frac{r_{\mathrm{work}}-{r}_{\mathrm{CFmax}}}{\tau_{\mathrm{form}}} $$

Some of the simplifying assumptions in the model are regarding the current conduction in the LRS and HRS. During the LRS, the conduction is assumed to be Ohmic, i.e., it follows Ohm’s law. In the HRS region, the current is dominated by a leakage current in the sub-oxide region which is basically due to trap-assisted conduction, but for simplicity sake, Ohmic conduction is considered here. The SET/RESET operation in the model is described by the electrochemical redox reaction derived from the Butler-Volmer equation [162] given as [92]:

60 $$ {\tau}_{\mathrm{Red}}={\tau}_{\mathrm{Red}\mathrm{ox}}\times {e}^{\frac{E_a-q\times {\alpha}_s\times {V}_{\mathrm{cell}}}{k_b\times T}} $$

61 $$ {\tau}_{Ox}={\tau}_{Redox}\times {e}^{\frac{E_a+q\times \left(1-{\alpha}_s\right)\times {V}_{\mathrm{Cell}}}{k_b\times T}} $$

Here, τ_Red_ and τ_Ox_ are the reduction and oxidation reaction rates, respectively. τ_Redox_ is the effective reaction rate considering both the reduction and oxidation reactions. Above two equations are coupled together in a master equation which define the switching rate given as [92]:

62 $$ \frac{d{r}_{CF}}{dt}=\frac{r_{CF max}-{r}_{CF}}{\tau_{red}}-\frac{r_{CF}}{\tau_{Ox}} $$

This is quite a comprehensive model in the sense that it includes the temperature effects as well. Temperature plays a significant role in the redox reaction rates [163, 164] and thus the local temperature in the filament is a very important parameter in this regard. The basic heat equation is used in this model and modified it accordingly given as [92]:

63 $$ \sigma (x)\times E{(x)}^2=-k\times \frac{\partial^2T(x)}{\partial {x}^2} $$

64 $$ T(x)={T}_{\mathrm{amb}}+\frac{v_{\mathrm{Cell}}^2}{2\times {L}_x^2\times k}\times \left(\frac{L_x^2}{4}-{x}^2\right)\times {\sigma}_{\mathrm{eq}} $$

65 $$ T={T}_{\mathrm{amb}}+\frac{v_{\mathrm{Cell}}^2}{8\times k}\times {\sigma}_{\mathrm{eq}} $$

66 $$ {\sigma}_{\mathrm{eq}}={\sigma}_{\mathrm{CF}}\times \frac{r_{\mathrm{CF}}^2}{r_{\mathrm{work}}^2}-{\sigma}_{Ox}\times \frac{r_{\mathrm{CF}\mathrm{max}}^2-{r}_{\mathrm{CF}}^2}{r_{\mathrm{work}}^2} $$

On the face of it, the equations seem pretty complex to evaluate. But in reality, they are analytical in nature which makes them easily solvable in a numeric solver and can be implemented in an electric simulator. This is a major advantage of this model. Almost all of the models which employ the concept of temperature change in the filament follow the basic principles of the filament dissolution model [82, 83] discussed further in the “Filament Dissolution Model” section. During set operation, the temperature rises due to the increase in the CF radius, while it falls due to a decrease in the CF radius during the reset operation. This creates a positive feedback loop between the two processes leading to a self-accelerated reaction. This forms the basis of the filament dissolution model and all models incorporating the temperature effects in the device converge on this phenomenon [82, 83, 86–89, 92].

**Fig. 12:**
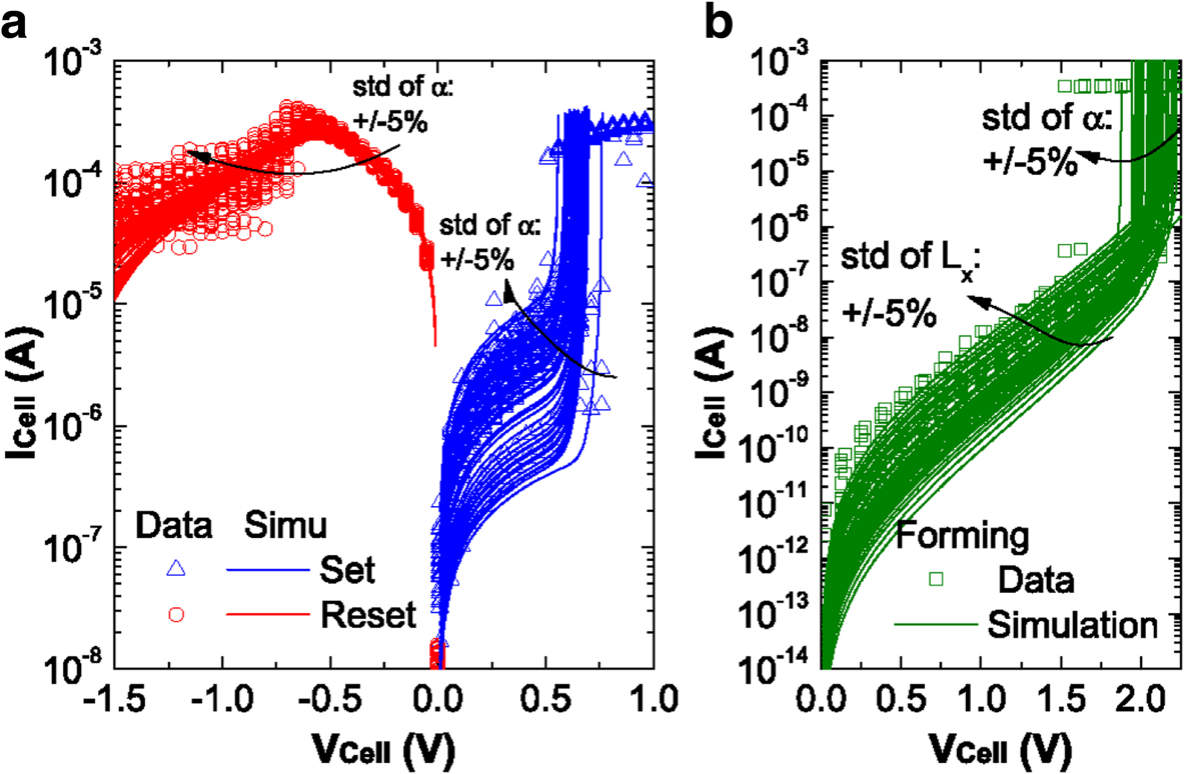
**a**, **b** Experimental *I (V)* characteristics for *Electroforming*, *Set*, and *Reset* processes measured on a large number of memory elements to understand the device-to-device variability. The experimental device-to-device variability is accounted for in Monte Carlo simulations with a ± 5% standard deviation on parameters *α* and *L*_*x*_ [21]

*I*-*V* characteristics of NiO based RRAM along with simulated curve using Bocquet model is presented in Fig. 12 [91]. Figure 12a represents the *set* and *reset* transitions of the device while Fig. 12b highlights the forming process. The current conduction in the Bocquet bipolar model is treated a little differently from what we have seen from previous models [87, 88, 90]. It considers the current as a combination of contributions from three different sources. The first one is the current from the conductive area (*i*_CF_), the second is the conduction through the switchable sub-oxide (*i*_sub-oxide_) and then the conduction through the pristine device (*i*_pristine_). The total current is described as [92]:

67 $$ {i}_{\mathrm{cell}}={i}_{\mathrm{sub}-\mathrm{oxide}}+{i}_{\mathrm{CF}}+{i}_{\mathrm{pristine}} $$

68 $$ {i}_{\mathrm{CF}}=E\times \pi \times {\sigma}_{\mathrm{CF}}\times {r}_{\mathrm{CF}}^2 $$

69 $$ {i}_{\mathrm{sub}-\mathrm{oxide}}=E\times \pi \times {\sigma}_{ox}\times \left({r}_{\mathrm{CF}\mathrm{max}}^2-{r}_{\mathrm{CF}}^2\right) $$

70 $$ {i}_{\mathrm{pristine}}={S}_{\mathrm{cell}}\times {A}_e\times {E}^2\times \exp \frac{-{B}_e}{E} $$

71 $$ {A}_e=\frac{m_e\times {q}^3}{8\pi \times h\times {m}_e^{ox}\times {\phi}_b} $$

The parameter B_e_ is metal-oxide barrier height (*ϕ*_*b*_), dependent which is given as [92]:

$$ \mathrm{if}\ {\phi}_b\ge q{L}_xE:{B}_e=\frac{8\pi \sqrt{2{m}_e^{ox}}}{3\times h\times q}\left[{\phi}_b^{\frac{3}{2}}-{\left({\phi}_b-q{L}_xE\right)}^{\frac{3}{2}}\right] $$

72 $$ \mathrm{otherwise}:{B}_e=\frac{8\pi \sqrt{2{m}_e^{ox}}}{3\times h\times q}\times {\phi}_b^{3/2} $$

Here, S_cell_ is a section of the RRAM cell. *A*_e_ and *B*_e_ are additional parameters defined to make the equations concise. To implement the model in an electrical simulator, discrete solutions are required which are well provided in this model. This makes the model suitable for proper simulation involving electrical circuits, thus widening its use case scenario. In this model, the equations are implemented in an Eldo circuit simulator [165, 166]. Memory effect of the device was replicated in the form that for each cell of the RRAM instance during transient simulation, the previous state of the filament as well as the applied voltage are given as the present state of the device [92]. New state gets solved as a function of these new inputs and the time step considers in the transient simulation. The discrete solutions are given as [92]:

73 $$ {r}_{{\mathrm{CFmax}}_{i+1}}=\left({r}_{{\mathrm{CFmax}}_i}-{r}_{\mathrm{work}}\right)\times {e}^{\frac{-\Delta  t}{\tau_{\mathrm{Form}}}}+{r}_{\mathrm{work}} $$

74 $$ {r}_{{\mathrm{CF}}_{i+1}}=\left({r}_{{\mathrm{CF}}_i}-{r}_{{\mathrm{CF}\mathrm{max}}_i}\times \frac{\tau_{\mathrm{eq}}}{\tau_{\mathrm{Red}}}\right)\times {e}^{\frac{-\Delta  t}{\tau_{\mathrm{eq}}}}+{r}_{{\mathrm{CF}\mathrm{max}}_i}\times \frac{\tau_{\mathrm{eq}}}{\tau_{\mathrm{Red}}} $$

75 $$ \mathrm{where}\ {\tau}_{\mathrm{eq}}=\frac{\tau_{\mathrm{Red}}\times {\tau}_{\mathrm{Ox}}}{\tau_{\mathrm{Red}}+{\tau}_{\mathrm{Ox}}} $$

**Fig. 13:**
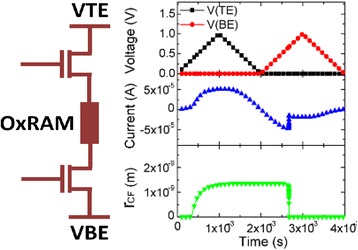
Simulating the electrical characteristics of the considered 2T-1R OxRAM structure [174]

The model has been verified against electrical characterization from an HfO_2_ based system [167]. To better judge the model on circuit level, a 2T/1R bipolar OxRAM [168] cell was simulated using Eldo, as shown in Fig. 13a [92]. Simulation of this type helps check the stability of the model when applied to a system environment. Current variation, voltage, and CF radius (*r*_CF_) are shown with respect to time. Voltage follows a triangular wave form, which is the input sweep. Current in the device transitions from high to low and vice-versa depend on the voltage levels. The sudden drop in the current levels, as shown in Fig. 13b [92], indicates the device transition. CF radius follows a similar path as the current which is expected behavior of the internal state variable.

Another important feature of OxRAM that has been highlighted in the model is the *soft-reset* [168]. It mainly induces the dependence between resistance in HRS and the stop voltage during the preceding reset operation. This phenomenon is basically due to the incomplete destruction of the CF during the reset process. So, the CF radius and temperature decrease during this process, leading to a decrease in the reaction rate. This means a self-limited reaction rate thus getting the name *soft-Reset*. This model can account for the device to device variability very efficiently [169, 170]. The standard deviation obtained for the important parameters such as the length of the oxide (L_x_) is well within the accepted range, thus accounting for the variations when the materials change in different devices [167, 168].

A shortcoming in the model which can be highlighted is the lack of a voltage or current threshold. Also, it works on the simplifying assumption that the CF radius grows from one end of the top electrode to the other end of the bottom electrode. This makes the model immune to significant fluctuations if the growth of the CF is not complete, thus leaving a filament gap. There is no provision to account for the effect of the filament gap if it occurs.

### Berco-Tseng Model

The proposed model and simulation approach [171–175] by Berco-Tseng for RRAM devices is based on describing the CF growth process. The Gibbs free energy criteria [174, 175] is used as an indicator to model the growth dynamics of the CF. Simulation approach for the forming, set and reset process in the model is based on the Metropolis Monte Carlo algorithm [174]. This approach importantly does not rely on time evolution of the CF, thus increasing the efficiency of comparison of the relative retention properties of MIM structures.

The model is quite comprehensive in terms of describing the underlying physical parameters which affect the CF kinetics in the resistive switching layer. It also introduces the concept of “hot-spots” [172–174] which are random localized initial clustering of oxygen vacancies which facilitate the formation of the CF. The major parameter governing the Gibbs free energy is the enthalpy of formation of an oxygen vacancy [174] is used to define the CF growth dynamics in the switching layer and integrate it into the Monte Carlo simulator. As a result, all the CF processes, namely forming, set and reset can be effectively simulated.

A monoclinic HfO_2_ switching layer is primarily used to implement the model. But other material stacks, such as ZrO_2_, Cu-HfO_2_ are also studied and compared with by us. Electrical conduction in the RRAM device is modeled on the Arrhenius relation, given as [174]:

76 $$ {\sigma}_n={\sigma}_0\exp \left(-\frac{E_{ac}}{k_B{T}_n}\right) $$

Typical boundary condition, such as *V*_dd_ at top electrode and ground at bottom electrode is applied to the device. For modeling the CF accurately, it is divided into a grid structure to discretize it, which is in line with the finite element analysis (FEA) method. The various parameters defining each grid site are its spatial coordinates (*x*, *y*), local potential *φ*, temperature *T*, *N*_*o*_, *N*_*ov*_, trap occupancy *c*, electrical conductivity *σ* and thermal conductivity *k*_*t*_*h*. The various processes associated with the evolution of CF within the oxide layer involves generation, recombination and hopping of oxygen (*O*), oxygen vacancies (*OV*) and electrons.

As a result, these processes are defined in terms of probabilities in the MC simulator. The probabilities are defines according to the minimum energy criteria as discussed earlier. This approach smoothens out the iterative steps in the simulator rather than the abrupt + 1 or − 1 levels in the discrete approach. The generation (*P*_g_), hopping (*P*_h_), and recombination (*P*_r_) probabilities for the oxygen species are given as [174]:

77 $$ {P}_{g,n}=\left(1-{C}_n\right)\exp \left(-\frac{E_a-{a}_a ZeE}{k_B{T}_n}\right) $$

78 $$ {P}_{h,n\to m}={C}_n\left(1-{C}_m\right)\exp \left(-\frac{E_h-{a}_h ZeE}{k_B{T}_n}\right) $$

79 $$ {P}_r={C}_n\exp \left(-\frac{\Delta  {E}_r}{k_B{T}_n}\right) $$

Here, *E* is the local electric field, *C*_n_ represents the ratio of *N*_ov_ (density of oxygen vacancies) in the low state to the maximal one at site *n* (*n*^th^ grid site). *E*_*a*_ and *E*_*h*_ are the activation energies for oxygen species generation and hopping respectively. Similarly, *a*_*a*_ and *a*_*h*_ are the field lowering factor for O generation and hopping.

The mechanism of the current conduction is considered to be trap-assisted tunneling [171, 173, 174] through the switching layer. The Mott variable hopping model [174, 175] is taken into account here to model the tunneling effect. Mott DC conduction considered at any two grid points *m, n* are given as [174]:

80 $$ {\sigma}_{mn}={f}_e\frac{e^2{c}_m\left(1-{c}_n\right)}{d_{mn}{k}_B{T}_n}\exp \left(-\frac{2{d}_{mn}}{\alpha}\right)\exp \left(-\frac{e\mid \nabla {\varphi}_{mn}\mid }{k_B{T}_n}\right) $$

Here, *d*_mn_ is the distance between *m* and *n*, α is the typical attenuation length of the electron wave function in the trap and *c* being the trap occupancy.

**Fig. 14:**
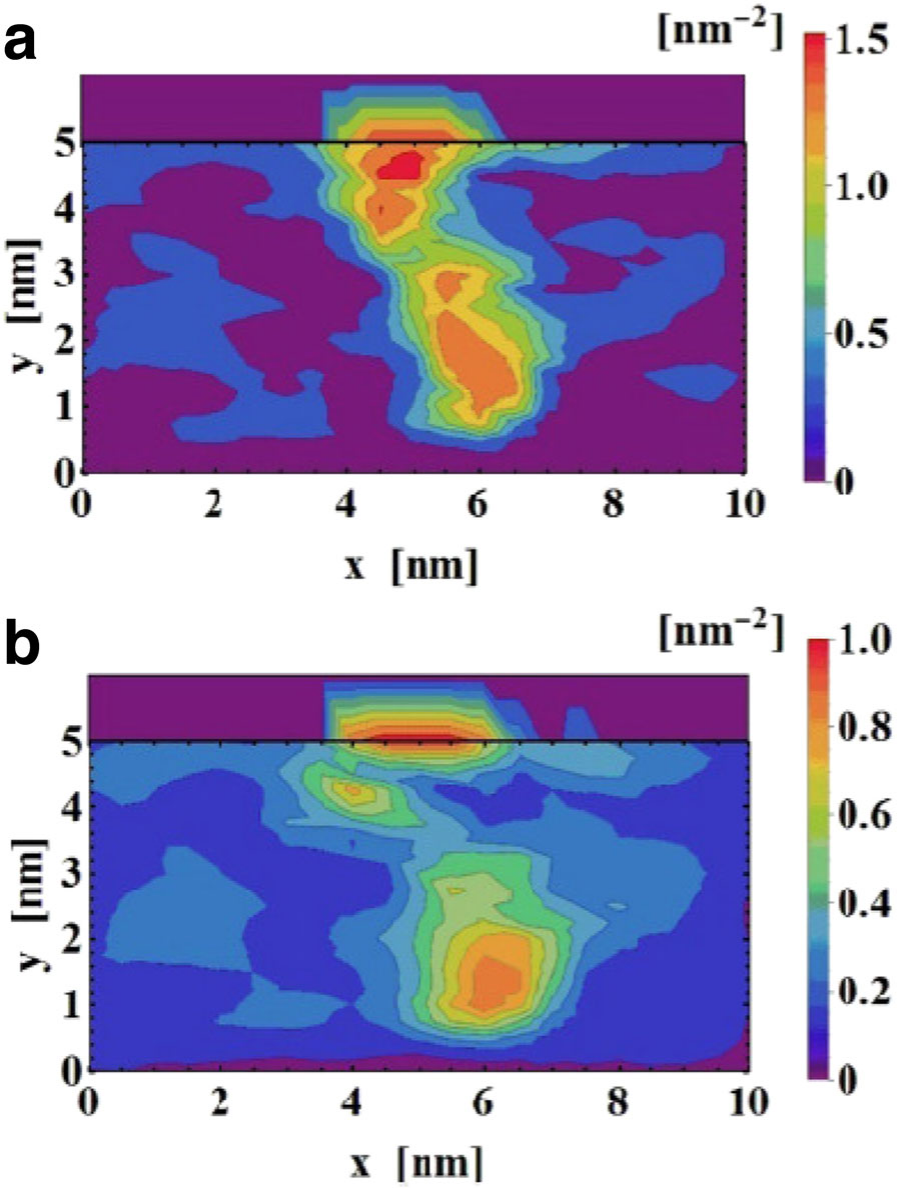
Resistive switching layer and Ti/HfO_2_ interface oxygen spatial density (No) plot during the CF rupture using negative bias: **a** initial and **b** final [174]

The model is simulated and the results are compared with many different material stacks of RRAM devices from other groups as well. The Fig. 14 shows the simulated heat map of the *N*_o_ (density of oxygen) concentration during the CF rupture process. The simulation result corroborates with the experimental results, showing wide applicability potential for the model.

### Gonzalez-Cordero et al. Bipolar Model

It is a compact physical model proposed by Gonzalez-Cordero et al. [93] describing the working of bipolar RRAM systems. The model is unique because it considers the CF as a truncated cone, which is a significant departure from previous models considering the CF shape generally as a cylinder. Also, the model is validated by implementing it in Verilog-A which gives us a closer look into the description and simulation of RRAM devices on the circuit level using Verilog-A. The proposed model builds upon the concepts introduced in the previous Bocquet bipolar model [91, 92] and modifies it accordingly to suit the new CF shape proposed.

One of the important aspects about the model is the consideration of a truncated cone shaped CF [176–179]. Majority of the models we have encountered till now consider the CF as a symmetrical cylinder which is more of a simplifying assumption [91, 92]. This is because it has been shown that the CF can grow [39, 51, 55] from one end of either electrode to the other depending on the active electrode. So, it is quite possible that the CF in this case might not be a perfect cylinder. So, a truncated cone is equipped to account for any variability and fluctuations arising due to the shape of the CF. Shapes other than simplified geometrical shapes are not considered in the models because of algebraic complexities. In previous models [91, 92], we noticed that device to device and cycle to cycle variability’s have a significant effect on the application of particular models to devices. So, by taking a truncated cone as the CF shape provides this model more flexibility than the others.

Another significant feature is the role of temperature in the CF and the reset process. Majority of the models which describe the CF rupture due to the self-accelerated dissolution, consider that the process takes place at the CF narrowing point and temperature increases at that particular point [82–84, 91, 92]. This point is generally in the middle of the cylindrical CF due to its symmetry. So, when we look at it from a physical standing point, the temperature at each of the points in all the RRAMs stacked together in a circuit has to be evaluated. Realizing this from the circuital standing point and simulating thousands of devices in the circuits is a very time-consuming process and slows down the simulation. This problem can be circumvented by considering two temperatures in the CF instead of the general single temperature; this approach also keeps the simplicity of the model intact. Two temperatures represent the main CF body that is not destroyed during the reset operation and the CF narrowing. This has been implemented in this model by considering the two temperatures as the wide region and the narrow region of the truncated cone, respectively.

This model extends the previously discussed Bocquet bipolar model [91, 92] in the “Bocquet Bipolar Model” section. In the previous model, the equations were defined keeping a cylindrical CF in mind, so the equations here have been modified to account for the change in the CF shape. The truncated cone CF is described by two different radii. CF is considered to grow from the top electrode to the bottom; the interface radius with the top electrode (TE) is *r*_CFT_ which is always greater than the radius of the interface with the bottom electrode (BE) *r*_CFB_. This adheres to the structure of the truncated cone. An assumption is made here that during CF rupture, height of the cone is not affected; this makes the model open to fluctuations if there is any filament gap produced due to premature growth of CF. Although a forming process is considered for the device, it is not included in the model making the model not suitable for application to devices where forming is a significant factor. A possible explanation for leaving out the forming process is to avoid adding more complexity to the model because the forming parameters have to be included in the set/reset equations as well.

Similar to the previous model, the set/reset processes are described by an electrochemical redox reaction and diffusion processes [82–84, 105, 161, 162] which control the growth and rupture of the CF, respectively. The reduction and oxidation reaction rates are given as [93]:

81 $$ \frac{1}{\tau_{{\mathrm{Red}}_{\left(T,B\right)}}}={A}_{\mathrm{Red}\mathrm{ox}}\times {e}^{-\frac{E_a-q\times {\alpha}_s\times {v}_{\left(T,B\right)}}{k_B\times {T}_{{\mathrm{CF}}_{\left(T,B\right)}}}} $$

82 $$ \frac{1}{\tau_{{\mathrm{Ox}}_{\left(T,B\right)}}}={A}_{\mathrm{Redox}}\times {e}^{-\frac{E_a-q\times \left(1-{\alpha}_s\right)\times {v}_{\left(T,B\right)}}{k_b\times {T}_{{\mathrm{CF}}_{\left(T,B\right)}}}} $$

Velocity of the CF radius increase/decrease which is basically the switching rate is controlled by two master differential equations which include the top (*r*_CF,T_) and bottom (*r*_CF,B_) radius of the CF. They are given as [93]:

83 $$ \frac{d{r}_{CF_T}}{dt}=\frac{r_{CF_{TM}}-{r}_{CF_T}}{\tau_{Red_T}}-\frac{r_{CF_T}}{\tau_{Ox_T}} $$

84 $$ \frac{d{r}_{CF_B}}{dt}=\frac{r_{CF_T}-{r}_{CF_B}}{\tau_{Red_B}}-\frac{r_{CF_B}}{\tau_{Ox_B}} $$

CF radius is set a boundary which defines the limits for the CF growth/rupture which is given as [93]:

85 $$ {r}_{CF_{Tm}}\le {r}_{CF_T}\le {r}_{CF_{TM}} $$

86 $$ {r}_{CF_B}\le {r}_{CF_{BM}} $$

Here r_CF,TM_/r_CF,BM_ is the maximum top/bottom radius that can be achieved and r_CF,Tm_ the minimum value of the top radius. This equation indicates CF geometry following a truncated cone structure and the top radius is greater than the bottom radius at all times. The model employs a numeric solving method similar to the one used in the previous model to find the discrete solutions for the master differential equations. But the solution for this model is not found, which means it is difficult to validate the reliability of the equations.

A very interesting point here is that a separate local diffusion process is not added in this model to describe the reset process in addition to the oxidation/reduction process. Many of the previously discussed models [91, 92] have a separate equation for the diffusion. But in this model diffusion has been integrated into the Eqs. (80) and (81) for redox reactions by considering different activation energies for the reduction and oxidation rates. This has been deliberately done considering the fact that the equation used in previous models to describe the diffusion velocity is similar in structure to the redox equations. As a result, the activation energies for both are combined together to consider a single activation energy which includes diffusion as well.

The current in the RRAM device is considered to be comprised of two components, the CF current (*i*_CF_) and the oxide current (*i*_ox_). In CF current, contribution of the CF resistance (R_CF_) and the oxide surrounding the CF included in the CF radius are given as [93]:

87 $$ {i}_{\mathrm{CF}}=\frac{v_{\mathrm{app}}}{R_{\mathrm{CF}}\Big\Vert {R}_{\mathrm{ox}}} $$

88 $$ {R}_{\mathrm{CF}}=\frac{L}{\pi \times {\sigma}_{\mathrm{CF}}\times {r}_{{\mathrm{CF}}_T}\times {r}_{{\mathrm{CF}}_B}} $$

89 $$ {R}_{\mathrm{ox}}=\left\{\begin{array}{c}\frac{L\times \beta }{2\times {\sigma}_{\mathrm{ox}}\times \pi \times {r}_{\mathrm{CF}\mathrm{ext}}\times \left({r}_{{\mathrm{CF}}_B}-{r}_{{\mathrm{CF}}_T}\right)},\kern0.75em {r}_{{\mathrm{CF}}_T}\ne {r}_{{\mathrm{CF}}_B}\\ {}\frac{L}{\sigma_{\mathrm{ox}}\times \pi \times \left({r}_{\mathrm{CF}\mathrm{ext}}^2-{r}_{{\mathrm{CF}}_T}^2\right)},\kern4.25em {r}_{{\mathrm{CF}}_T}={r}_{{\mathrm{CF}}_B}\end{array}\right. $$

The other component in the device current, i.e., the oxide current represents the current throughout the oxide accounting for the whole area of the device except the one occupied the CF. The oxide current is described as [93]:

90 $$ {i}_{\mathrm{ox}}=\operatorname{sign}\ \left({v}_{\mathrm{app}}\right)\times {A}_{\mathrm{HRS}}\times {S}_{\mathrm{Cell}}\times {\left\{\frac{\mid {v}_{\mathrm{app}}\mid }{L}\right\}}^{\alpha_{\mathrm{HRS}}} $$

**Fig. 15:**
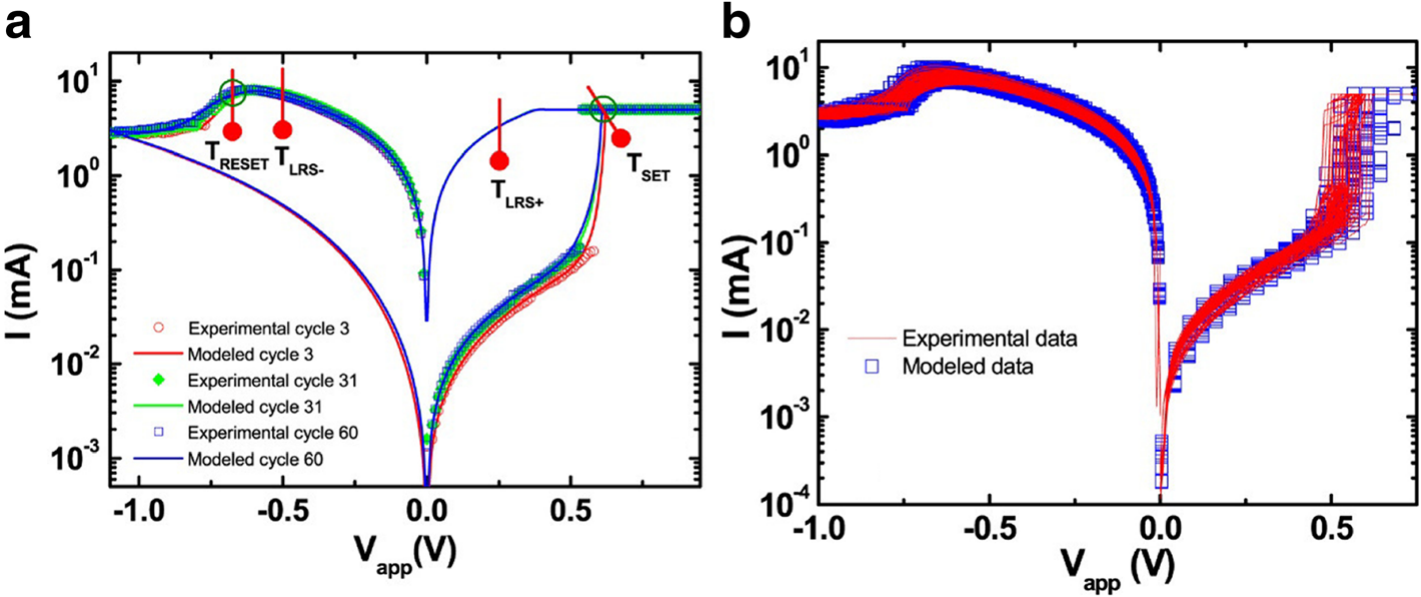
**a** RRAM current versus voltage for different cycles; the CF features are tuned to fit different curves such as *r*_CFTM_ = 45 nm, *r*_CFTm_ = 15 nm, and *r*_CFBM_ = 7 nm for cycle 3, *r*_CFTM_ = 45 nm, *r*_CFTm_ = 25 nm, and *r*_CFBM_ = 7 nm for cycle 31, and *r*_CFTM_ = 40 nm, *r*_CFTm_ = 35 nm, and *r*_CFBM_ = 7.8 nm for cycle 60; **b** *I*-*V* curves for the devices under consideration along with different modeled data (the CFs’ dimensions considered to fit the whole set of curves have been generated randomly). The temperature at the most representative points of the I–V curve, highlighted in **a** cycle 3: *T*_SET (T,B)_ = [579,656] K, *T*_LRS+ (T,B)_ = [349,368] K, *T*_LRS-(T,B)_ = [466,525] K, *T*_RESET (T,B)_ = [565,621] K; cycle 31: *T*_SET (T,B)_ = [573,669] K, *T*_LRS+ (T,B)_ = [349,368] K, *T*_LRS- (T,B)_ = [460,525] K, *T*_RESET (T,B)_ = [566,622] K; cycle 60: *T*_SET (T,B)_ = [564,685] K, *T*_LRS+ (T,B)_ = [347,367] K, *T*_LRS- (T,B)_ = [460,525] K, *T*_RESET (T,B)_ = [545,641] K [93]

Here, *A*_HRS_ and *α*_*HRS*_ are fitting constants. The experimental and simulated results from the model are illustrated in Fig. 15 [93]. The simulated *I*-*V* curves were tuned using device parameters to fit the reported experimental data [180]. The major device parameter which was tuned the CF radius. Simulated results are almost a perfect fit to the experiment results which highlights the flexibility of the model with the proper tuning of parameters.

When considering electrochemical redox reactions, temperature is a very important factor. As can be seen from the equations describing the set/reset process, temperature parameter is a defining factor. Modeling using this concept, temperature in the CF is described through a one-dimensional approach as given as [93]:

91 $$ {\sigma}_{\mathrm{CF}}\times E{(x)}^2=\frac{2{c}_h}{r_{\mathrm{CF}}(x)}\left({T}_{\mathrm{CF}}(x)-{T}_{\mathrm{ox}}\right)-k\frac{\partial^2{T}_{\mathrm{CF}}(x)}{\partial {x}^2} $$

However, this approach is not the best one for truncated cones. It leads to increased complexity and improper calculation of temperature. So, Gonzelez-Cordero et al. [93] proposed a different approach where they have developed simplified analytical equations which are suitable for simulation. In the previous Bocquet model [92] they have assumed a cylindrical uniform geometry where the calculated unique temperature is uniform throughout the CF. But to consider a more detailed physical description in this model; they have considered two temperatures in the CF. One temperature is at the hottest CF point where the reset process ruptures the CF and the other temperature is considering the main CF volume. This is a more reasonable model for a truncated cone structure as the two radii grow independently of each other depending on the oxidation/reduction processes [171, 181].

To mask this concept into a simplified analytic expression, a simplifying assumption is made. Uniform cylinders are considered to represent the truncated cone CF; one corresponding to the larger radius of the cone, i.e., *r*_CF,T_ and the other to the smaller radius, i.e., *r*_CF,B_. So, the maximum temperature calculated for each cylinder considering the main CF volume (*T*_CF,T_) and at the narrowest part (T_CF,B_) is given as [93]:

92 $$ {T}_{{\mathrm{CF}}_{\left(T,B\right)}}={T}_{\mathrm{amb}}+\frac{\sigma_{{\mathrm{CF}}_{\left(T,B\right)}}\times {r}_{{\mathrm{CF}}_{\left(T,B\right)}}\times {E}_{\left(T,B\right)}^2}{2\times {c}_h}\left(1-\cos h\left(\frac{\alpha_{\left(T,B\right)}L}{2}\right)\right)+\frac{d{T}_{0_{\left(T,B\right)}}}{\alpha_{\left(T,B\right)}}\sin h\left(\frac{\alpha_{\left(T,B\right)}L}{2}\right) $$

93 $$ {\alpha}_{\left(T,B\right)}=\sqrt{\frac{2h}{k\times {r}_{{\mathrm{CF}}_{\left(T,B\right)}}}} $$

94 $$ d{T}_{0_{\left(T,B\right)}}=\frac{\sigma_{{\mathrm{CF}}_{\left(T,B\right)}}\times {r}_{{\mathrm{CF}}_{\left(T,B\right)}}\times {\xi}_{\left(T,B\right)}^2\tan h\left(\frac{\alpha_{\left(T,B\right)}L}{2}\right)}{\sqrt{2\times k\times {c}_h\times {r}_{{\mathrm{CF}}_{\left(T,B\right)}}}} $$

95 $$ {\sigma}_{{\mathrm{CF}}_{\left(T,B\right)}}=\frac{\sigma_{{\mathrm{CF}}_0}}{1+{\alpha}_T\left({T}_{{\mathrm{CF}}_{\left(T,B\right)}}-{T}_{\mathrm{amb}}\right)} $$

Here, *α*_*T*_ is the conductivity temperature coefficient. The model is simulated and compared with the results from the previous Bocquet bipolar model [93] on which it is based. The results compare the findings from the model considering a cylindrical CF to the one considering the truncated cone CF. There is some evidence [93] presenting a better fit with the experimental data for this particular model as compared to the previous models where cylindrical CFs are considered and also results pertaining to the cases where multiple CFs are also presented; this shows the model’s flexibility in accommodating devices where multiple CFs is existent.

**Fig. 16:**
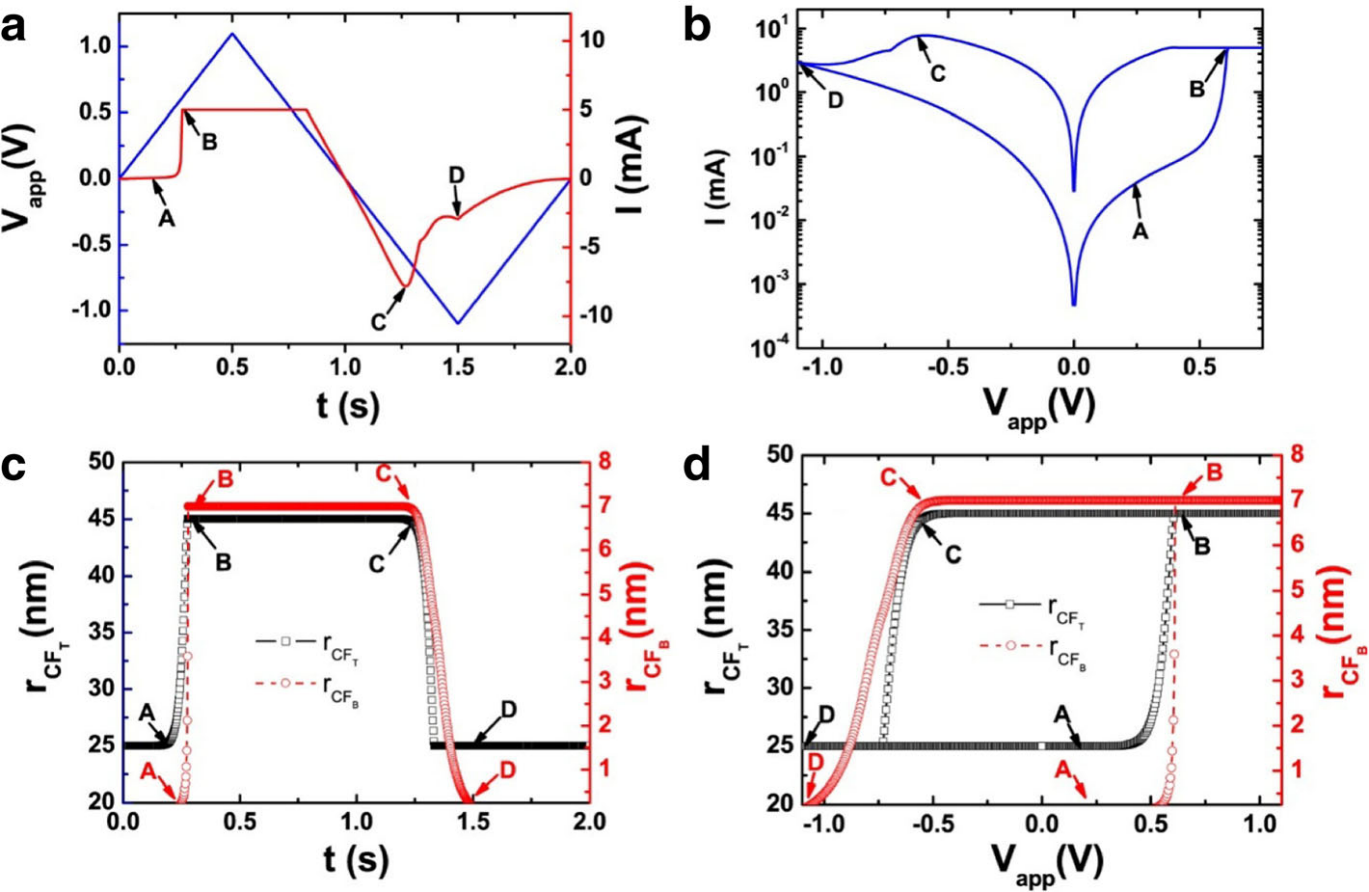
**a** Applied triangular voltage signal (blue) and corresponding device current (red) versus time. **b** The RRAM current against the applied voltage. **c** Top and bottom CF radii versus time for the devices under consideration. **d** Top and bottom CF radii corresponding to figure 7(c) versus applied voltage [93]

Detailed simulation results presenting the variation of current, voltage and CF radius are shown in Fig. 16 [93]. The variation of the applied voltage and the device current with time is shown in Fig. 16a. The complete simulated *I-V* curve can be seen in Fig. 16b which follows the form of a hysteresis loop, suggesting resistive switching behavior. Variations of the CF radius with time and applied voltage are presented in Fig. 16c, d, respectively.

Primary aim of the model was to be simplistic enough to be implemented in electric circuit simulators. Analytical equations are properly laid out to be used in SPICE simulations and it has been represented through a 1T/1R circuit. The model is also represented through a Verilog-A representation [93] which shows its applicability in digital circuits as well.

## RRAM Models Based on Unipolar Devices

### Random Circuit Breaker Network Model

In 2008, Noh’s et al. [182] proposed a random circuit breaker model to explain the switching in unipolar resistive switching devices. This model evolved to clear the considerable debate regarding the switching mechanism in unipolar devices, at the early stage of the study mechanism of resistive switching memory. Some reported that switching is the result of homogeneous/non-homogeneous transition of current distribution, while some other says due to the formation and rupture of conducting filaments. A new percolation model was reported by Noh’s group [182] in this regard which was based on a network of circuit breakers with two switchable metastable states. The device used for the study is a polycrystalline TiO_2_ RRAM device. It shows wide distributions of SET and RESET voltage with uniform resistance change at the particular transition voltage. Conductive atomic force microscopy (C-AFM) tip was used as a top electrode, and external voltage was applied through it for the resistive switching operations.

**Fig. 17:**
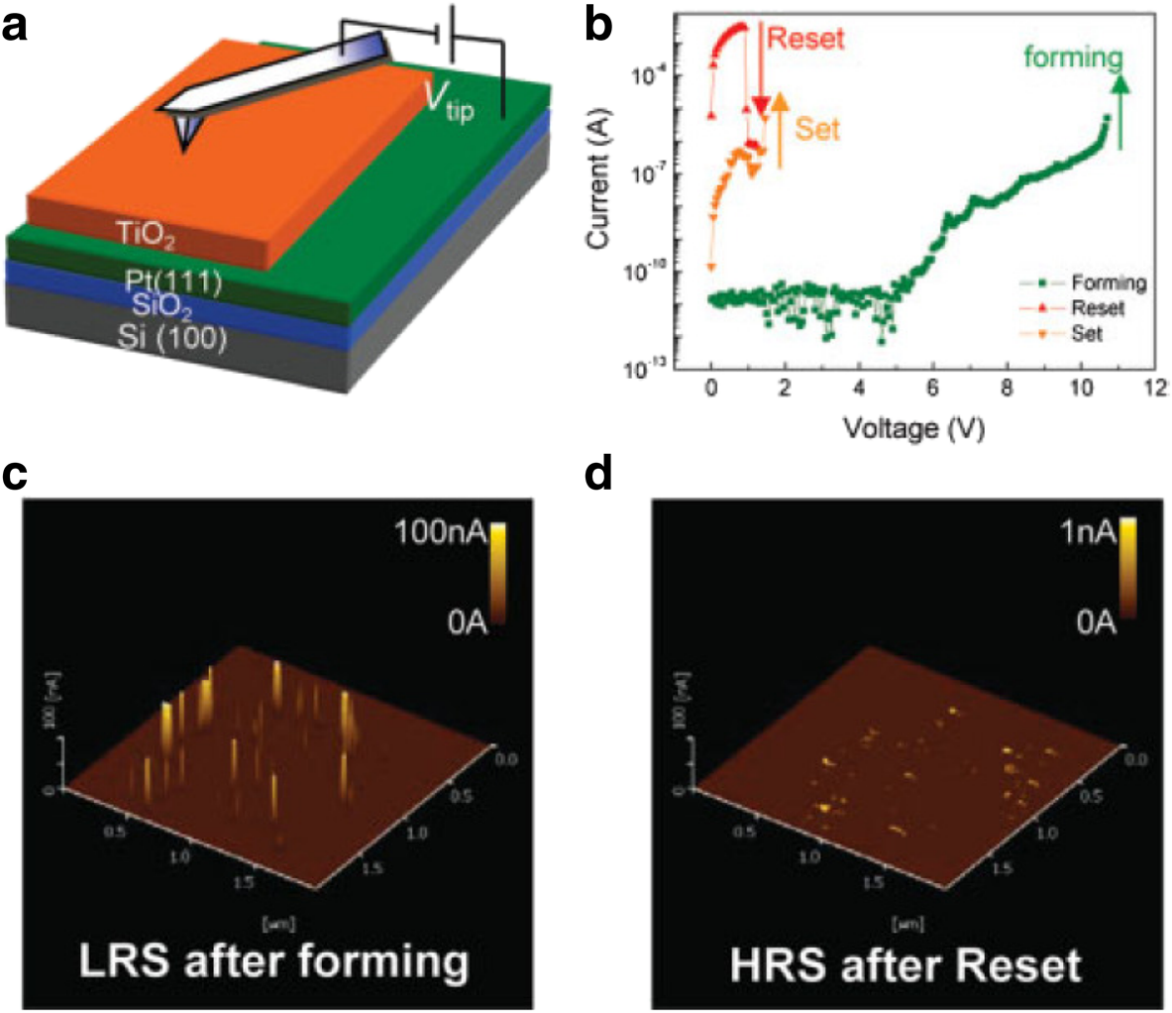
Conducting AFM (C-AFM) topographs of TiO_2_ films. **a** Schematic of C-AFM measurements (**b**) Typical *I*-*V* curves of TiO_2_ films using C-AFM tip as a top electrode. **c** At *V*_tip_ = 8 V, mapping of the current flow through the surface just after the forming operationshows locally distributed conducting regions. **d** TiO_2_ surface in the HRS shows locally distributed conducting regions disappear after reset operation with *V*_tip_ = 1 V [182]

The C-AFM images along with the switching curve are shown in Fig. 17a, b. The current map shows the state of the system in the LRS and HRS. In LRS, many conductive spots can be observed in the current map which can be considered to be conducting filaments. Corresponding to the HRS, the conducting spots are vanishing, which could be translated as the rupture of the filaments. This behavior was qualitatively described by a percolation model comprising of random circuit breakers (RCB), termed as RCB network model, and is shown in Fig. 17c, d. In this model, the RRAM system is considered as a combination of a number of circuit breaker, which can have either two resistance states, high (OFF state) or low (ON state). When the ON state circuit breaker receives the RESET voltage, it switches to the OFF state. Conversely, when the OFF state circuit breaker receives the SET voltage, it switches to the ON state.

This model basically laid the foundation for future percolation models used to describe RRAM switching behavior, since it is dealt with the stochastic reversible dynamic processes. Most percolation models either investigate static cluster topology problems or dynamic percolation problems. A combination of reversible and dynamic processes is quite interesting. This also enabled future model developers to account for stochastic switching in the physical equations describing unipolar RRAM devices.

### Filament Dissolution Model

Filament dissolution model was proposed by Russo et al. [82–84] exclusively for unipolar RRAM devices, although later revisions by the same group [85, 86] made this model suitable for bipolar devices as well. This model is based on the fundamental concept of Joule heating and filament temperature change. The model primarily focuses on the RESET transition of the devices, i.e., the transition from LRS to HRS. This is because of the high resistance associated with the RESET transition and this is where the major physical transformation in the device takes place. The model is based on the concept of conductive filament ruptured or dissolute under the effect of significant temperature change [84]. This temperature change in the filament is caused due to Joule heating. The proposed filament dissolution model has been deemed as self-accelerated due to the process of the rupture of filament accelerates by itself under suitable conditions.

Major advantage of this model is that it makes use of simple well-known coupled partial differential equations which describe the various effects in the device. The model is applied on a NiO based unipolar system [82, 83] where the oxide layer is sandwiched between two Pt electrodes and the filament is considered to grow from one end of the electrode to the other. Temperature profile in the oxide layer across its geometry is considered as parabolic; meaning that the temperature in the filament is minimum at the electrodes and maximum at the middle.

The mechanism behind the filament dissolution can be explained by the basic concept of Joule heating and dissolution which acts as an activator for the CF rupture. With the application of bias across the top electrode of the device, heat is produced in the filament due to the current flowing. The temperature steadily rises with an increase in the bias and when the bias reaches a significant level called reset voltage, the temperature rises above a value called the critical temperature. At this point, the dissolution of the filament is activated and the filament gets ruptured at a very fast rate leading to the device reaching a HRS.

Filament dissolution model uses coupled partial differential equations to describe the current in the device, the temperature changes due to Joule heating and the dissolution velocity. The current conduction in the device is described by the Poisson’s equation [83]:

96 $$ \nabla \times \left(\frac{1}{\rho}\nabla v\right)=0 $$

Here, *ρ* is the resistivity of the oxide and *v* the electric potential developed in the device due to the application of an external bias voltage *v*_term_. The voltage bias is applied at one of the electrodes, while the other electrode is connected to ground which act as boundary conditions for the device. NiO is the switching oxide, and the CF is formed as a sub-layer comprising primarily of metal ions and oxygen vacancies. The CF is considered to have a diameter of *φ*_*d*_.

As a result of the potential across the electrodes, heat is produced in the device due to Joule heating which leads to an increase in the temperature inside the CF. The CF geometry is divided into a number of mesh grids, to compare the ionic motions during filament formation and dissolution. The temperature is calculated at each part of the mesh grid to describe the thermal dissolution or rupture of the CF. This effect is described by the Fourier steady state heat equation given as [83]:

97 $$ -\nabla \times \left(k\nabla T\right)=\rho {J}^2 $$

Where k is the thermal conductivity of the oxide layer, T is the device temperature and J is current density. Thermal conductivity and electrical resistivity values are dependent on the position they are applied in. So, *ρ* = *ρ*_CF_ inside the CF while it is equal to *ρ*_*OX*_ in the oxide layer. The same analogy applies for the values of thermal conductivity as well. The temperature is considered to be equal to room temperature *T*_0_ at the electrodes, i.e., they act as heat sinks.

The device temperature *T* increases up to the point of reset, where it reaches critical temperature, after which the CF dissolution takes place. As a result of the rupture of CF, the current conduction is interrupted which transitions the device into HRS or reset state. This dissolution factor is modeled as [83]:

98 $$ {\nu}_{\mathrm{DIS}}={\nu}_{\mathrm{DIS}-\mathrm{F}}{e}^{-\frac{E_a}{k_BT}} $$

Where, *E*_*a*_ is the activation energy, *k*_*B*_ is the Boltzmann constant, *v*_DIS-F_ is a fitting parameter and *v*_DIS_ is the velocity of the CF boundary toward the symmetry axis.

The temperature dependent resistivity in the CF can be described as [83]:

99 $$ {\rho}_{\mathrm{CF}}(T)={\rho}_{\mathrm{CF}-\mathrm{RT}}\left[1+c\left(T-{T}_0\right)\right] $$

where *c* is the experimentally calculated temperature coefficient of resistivity and *ρ*_CF-RT_ the standard CF resistivity at room temperature.

**Fig. 18:**
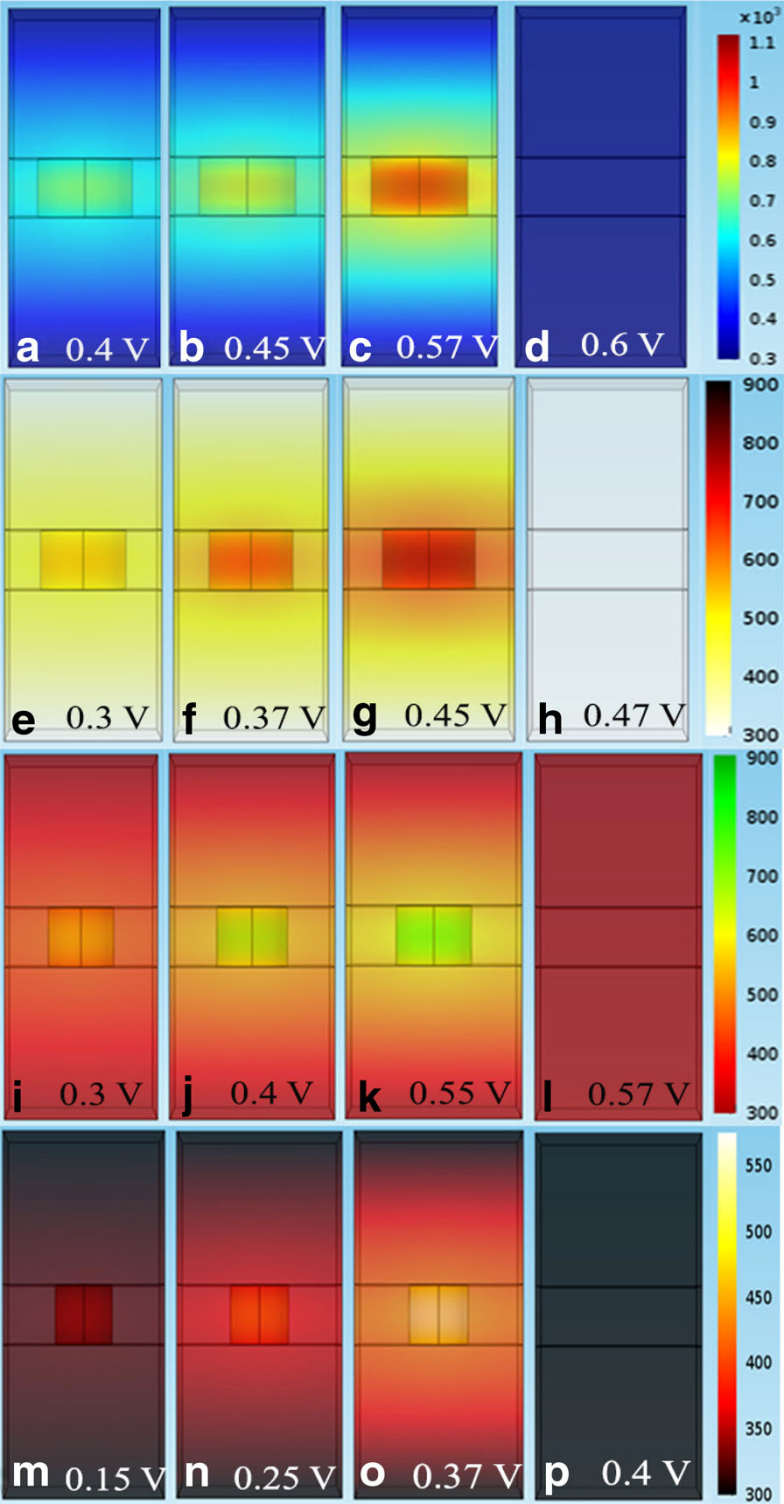
Calculated temperature maps extracted from the electro thermal simulations for applied voltages **a** 0.4 V, **b** 0.45 V, **c** 0.575 V, and **d** 0.6 V for sample P (500 °C annealed); **e** 0.3 V, **f** 0.375 V, **g** 0.45 V, and **h** 0.475 V for sample Q (400 °C annealed); **i** 0.3 V, **j** 0.4 V, **k** 0.55 V, and **l** 0.575 V for sample R (300 °C annealed); **m** 0.15 V, **n** 0.25 V, **o** 0.375 V, and **p** 0.4 V for sample S (200 °C annealed) across the CF geometry of length *t*_ox_ *=* 20 nm. Different bias points are considered for the devices so that the reset transition for each device can be visualized easily [185]

The coupled equations defined in the model are self-continuously solved using the numerical solver COMSOL [183, 184] Multiphysics. This software is well suited to handle these types of simulations owing to its Multiphysics capabilities. The model here uses mechanisms from electrostatics as well as heat transfer, so to simultaneously being able to handle multiple physical phenomenon’s is a big advantage on the part of the software. The obtained simulation results are shown in Fig. 18 [185]. The results show the variation in CF temperature with the change in voltage for three samples [185]. It follows the expected pattern of the model where the CF is dissolved after reaching the *reset* state.

Filament dissolution model discussed here has been modified and presented for bipolar RRAM devices by Larentis et al. [85, 86]. It is based on the same temperature and field accelerated ion migration. The set and reset processes in the device are defined by the mechanisms of drift migration induced by local electric field, ionic/electronic conduction and Joule heating. This is a point of departure from the model for the unipolar devices where the switching mechanisms for the set state were not properly defined and understood.

As a result, an equation explaining the ion migration given in Eq. (48) is introduced in the model. It includes both drift and diffusion components which rely on ion hopping. Rate of drift and diffusion is generally governed by the external applied bias and the amount of barrier lowering caused by it which is critical for the process of ion hopping. Barrier lowering generally takes place in the direction of applied electric field *F*, and the ion hopping depends exponentially on this energy barrier value. This causes significant migration of ions in the direction of the electric field *F*. This is a very simplistic and acceptable physical approach to explain the filament formation due to the charge carriers such as metal ions or oxygen vacancies. This also explains the dependence of the filament formation on the polarity of the external bias in bipolar devices. The ion-migration factor can be defined as [86]:

100 $$ {J}_D={J}_{\mathrm{diff}}+{J}_{\mathrm{drift}}=-{D}_s\nabla {n}_D+\mu E{n}_D $$

Where, *n*_*D*_ is the doping density, *D*_*s*_ is ion diffusivity, E is applied electric field and μ is the ion mobility. The temperature activated ion diffusivity, based on the Arrhenius law and ion mobility [85] is given as [86]:

101 $$ {D}_s={D}_0{e}^{-\frac{E_a}{kT}} $$

102 $$ \mu =\frac{q{D}_s}{kT} $$

Using the above equations, the continuity equation for the drift-diffusion is given as [86]:

103 $$ \frac{\partial {n}_D}{\partial t}=\nabla \times \left({D}_s\nabla {n}_D-\mu E{n}_D\right) $$

Another difference from the model applied for unipolar devices is the use of conductivity values rather than resistivity. Also, resistivity had a linear variance with temperature in the previous model [82, 83] while in this case conductivity is modeled to be exponentially dependent on temperature given as [86]:

104 $$ \sigma ={\sigma}_0{e}^{-\frac{E_{ac}}{k_BT}} $$

The current conduction defined in Eq. (95) and Joule heating described in Eq. (96) are used in the model for the bipolar devices. This suggests an assumption that the temperature profile for both types of devices follows a similar pattern. Along with it, the current conduction mechanism is also assumed to be similar. This in a sense might be an over-simplified assumption because many of the models described for bipolar resistive switching devices have been unable to be used for unipolar RRAM devices [90–92, 186] due to a marked mismatch in the conduction and switching mechanisms.

**Fig. 19:**
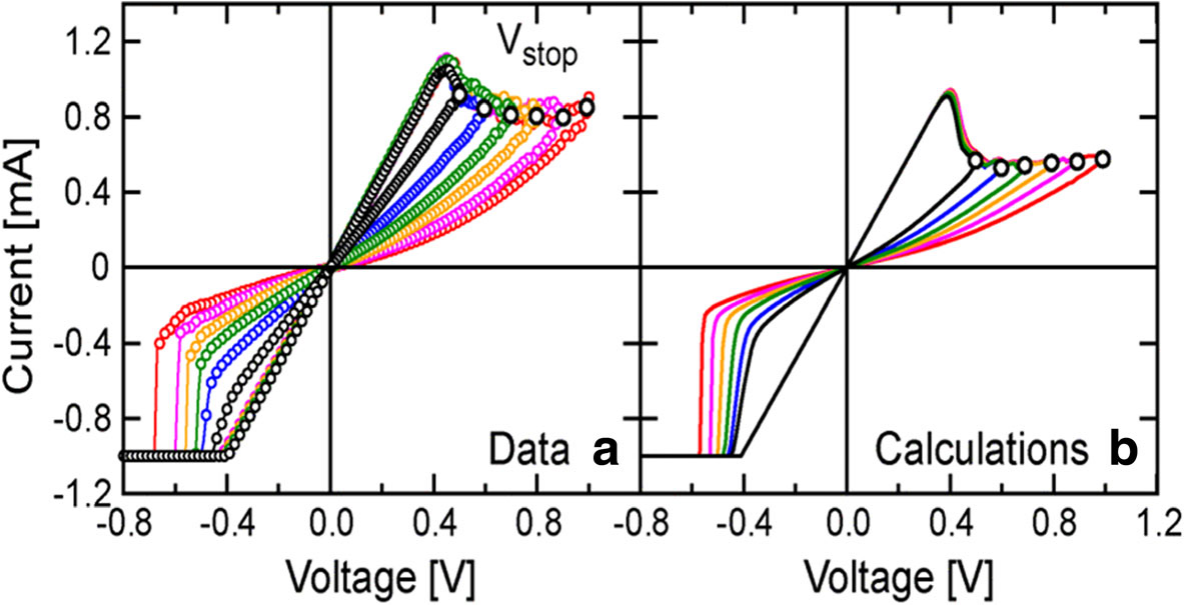
**a** Measured and **b** calculated *I*-*V* curves, for reset (*V >* 0) and set (*V <* 0) transitions, obtained by applying triangular voltage sweeps. For preparing a reset state with variable *R*, the reset sweep applied to an initial set state with *R* = 400 Ω, is interrupted at *V*_stop_. Then, the set sweep is applied, showing that *V*_set_ increases with *V*_stop_, hence with *R* [86]

The differential Eqs. (95)–(98) are simulated in COMSOL Multiphysics considering a 3-D cylindrical symmetrical geometry. The oxide material system considered here for reference is HfO_2_ and the obtained simulation results are verified with the experimental results as shown in Fig. 19 [86]. Device shows bipolar switching characteristics owing to the extra terms added in the model equations. There is somewhat of a good match between the experimental data and the calculated results. This suggests that this model could be potentially used to model bipolar devices. It is quite comprehensive in its definition of device parameters and also agrees with experimental data.

### Bocquet Unipolar Model

This model was developed by Bocquet et al. [90] for describing both the set and reset processes in unipolar RRAM devices. It is basically a modified extension of the model proposed by Russo et al. [82, 83] in the sense that it can model both the transitions of the RRAM device while the former only considers reset transition. For set process, a local electrochemical reduction of the oxide is considered to be responsible for formation of conductive filaments. However, the reset mechanism follows the tried and tested formula for unipolar devices which considers thermally assisted destruction of the formed metallic filaments by Joule heating as the primary mechanism. Also, it has to be mentioned that the model proposes equations which are analytical in nature and can be conveniently solved in an electric circuit solver.

The CF growth and rupture in the device is described by a local redox reaction and a thermally assisted diffusion respectively [82, 83, 105, 161] for a NiO based unipolar system. The reaction velocities for the reduction and oxidation processes during CF growth are based on the Butler-Volmer equation [162]. They are given by [90]:

105 $$ {\nu}_{\mathrm{red}}={k}_0{e}^{-\frac{\Delta  r{G}_0+2\left(1-{\alpha}_s\right)F\left(E-{E}_{eq}\right)}{P\times {T}_{\mathrm{ox}}}}\left(1-{C}_{Ni}\right) $$

106 $$ {\nu}_{\mathrm{ox}}={k}_0{e}^{-\frac{\Delta  r{G}_0-2{\alpha}_sF\left(E-{E}_{\mathrm{eq}}\right)}{P\times {T}_{\mathrm{CF}}(x)}}\times {C}_{Ni} $$

where ΔrG_0_ is the reaction free energy at equilibrium, *E*_eq_ is the equilibrium constant, *α*_s_ is the asymmetry factor, *P* is the ideal gas constant, *k*_0_ kinetics constant of chemical reaction, *C*_Ni_ is the dimensionless concentration of metallic species, and *F* is Faraday constant.

The above redox reaction describing Eqs. (104) and (105) can account for the CF growth, i.e., the SET process. But to explain the CF dissolution or the RESET process, it uses the filament dissolution model by Russo et al. [82, 83] as described further in the “Filament Dissolution Model” section. As discussed, the filament dissolution model uses the Joule heating mechanism to account for the *reset* transition in the device. The local diffusion velocity (ν_diff_) which is the governing equation for the filament dissolution model is given as [90]:

107 $$ {\nu}_{\mathrm{diff}}={k}_{\mathrm{diff}}\times {e}^{-\frac{E_q}{k_b\times {T}_{\mathrm{CF}}(x)}}\times {C}_{Ni} $$

As the dissolution velocity is exponentially dependent on temperature, it gets activated only when there is significant amount of temperature. Temperature value is high only when there is a comparable amount of voltage, i.e., the reset voltage has to cross a critical value during CF dissolution. This acts as means of a self-activation voltage threshold where the voltage controls the CF dissolution.

The set of reduction, oxidation and dissolution equations are coupled together in a master equation which controls the switching rate of the device given as [90]:

108 $$ \frac{d{C}_{Ni}}{dt}={\nu}_{\mathrm{red}}-{\nu}_{\mathrm{ox}}-{\nu}_{\mathrm{diff}} $$

The coupled equations in this model have to be solved simultaneously and continuously due to the fact that the model relies on self-consistent kinetic equations accounting for both CF growth and destruction mechanisms. This is a key feature which has to be implemented when using simulation tools to attain numerical accuracy.

Current conduction and temperature change in the device are described using simple current and heat flow equations. Current in the device on application of a voltage *v*_cell_ across the electrodes is given by [90]:

109 $$ {i}_0=\frac{v_{\mathrm{cell}}}{\int_0^{t_{\mathrm{ox}}}R(x) dx} $$

110 $$ R(x)=\frac{1}{r_{CF}^2(x)\pi \left({\sigma}_{CF}(x)-{\sigma}_{\mathrm{ox}}\right)+{r}_{{\mathrm{CF}}_{\mathrm{max}}}^2\times \pi {\sigma}_{\mathrm{ox}}} $$

Current flowing in the device gives rise to a temperature due to Joule heating, and this effect is modeled as [90]:

111 $$ {\sigma}_{\mathrm{CF}}(x)\times E{(x)}^2=-k\frac{\partial^2{T}_{\mathrm{CF}}(x)}{\partial {x}^2}+{c}_h\frac{T_{\mathrm{CF}}(x)-{T}_{\mathrm{ox}}}{t_{\mathrm{ox}}} $$

Numerical values obtained from simulation in general profoundly interlinked between the set and reset transitions. This is because from a practical stand point the CF profile obtained after the *set* operation is used as the initial state to simulate the subsequent *reset* operation. Also, the *reset* current and LRS resistance depends significantly on the maximum current reached during the previous *set* operation [187–189]. This is basically due to the minimization of the CF radius which subsequently increases the resistance of the device [190].

The Bocquet unipolar model is compared against a NiO system similar to the one used in the previous Filament dissolution model [82, 83]. It is to be noted that the model is applicable for a unipolar device only. But the comparison with the NiO system is limited to a single system using a numerical solver. This is a major shortcoming in this particular model regarding the non-availability of exact experimental characteristics data comparison from other sources to calibrate the model. It means that the fitting parameters have not been tested for a variety of characterization data or other models as well. So, it is difficult to judge the accuracy and viability of the model even though it uses some interesting concepts to explain the switching process in unipolar devices.

## Window Function Models

Window functions are introduced in the “Linear Ion Drift Model” section [3]. These functions are generally required to limit the values that the internal state variable can reach. The dynamics of the state variable governs the switching property of the device. So, the state variable has to be set bounds within which it can grow so that the device always remains in the permissible state and does not go out of bounds. For example, if the growth/rupture of CF is being modeled, the CF physically can only grow from one electrode end to the other. If the model growth crossed that limit, it suggests a mismatch between the physical phenomenon and the model. As a result, certain window functions [94–99] which acts as limiting functions is introduced into the model to set bounds for the device.

This is further required as near to the boundaries non-linear dopant drift effects take charge and heavily suppress the speed of the ions. As a result, it leads to a non-uniform and capricious rate of change of the state variable. To properly account for all the effects, an effective window function should have the following characteristics [98]:

- ➢Consider and take into account the boundary conditions at the top and bottom electrodes of the device.
- ➢Be capable of imposing non-linear drift over the entire active core of the device.
- ➢Provide linkage between the linear and non-linear dopant drift models.
- ➢Be scalable, meaning a range of *fmax*(*x*) can be obtained such that 0 ≤ *fmax*(*x*) ≤ 1.
- ➢Utilize an in-built control parameter for adjusting the model.

Window functions are generally multiplied with the particular *I*-*V* equation or state variable equation and are designed in a way so as to bind the complete equation within a certain boundary. There has been various window function based models proposed. In this section, we will review the most important and popular among them. We have also reported a comparative analysis among all the window functions in Table 2.

**Table 2:** Comparison of the window function implementations

Window Function	Symmetrical	Solves all boundary conditions	Accounts for non-linear effects	Scaling possible	Fits which model	DC response
Linear Ion Drift [3]	Yes	No	No	No	Linear ion drift	No
Joglekar [94]	Yes	No	Partially	No	Linear/non-linear/TEAM	No
Biolek [95]	Yes	Partially	Partially	No	Linear/non-linear/TEAM	No
Benderli-Wey [96]	NA	No	Partially	No	Linear	No
Shin [97]	NA	Almost all	Yes	Yes	Chua/linear drift	No
Prodromakis [98]	No	Almost all	Yes	Yes	Linear/non-linear/TEAM	No
BCM [99]	No	Partially	No	No	Linear drift	No
TEAM/VTEAM [75–77]	No	Partially	Practically yes	No	TEAM/TEAM for Simmons tunneling barrier	No

### Joglekar Window

One of the very first window functions proposed by Yogesh Joglekar and Stephen Wolf [94] is based on the linear ion drift model [3]. It was developed when memristors were still in its early stages of development after the breakthrough by the HP team proposed linear model. Window functions are aim to generalize the behavior of the model around the device boundary.

Linear ion drift model [3] in general accounted for majority of the characteristics of the memristor but where it fell short was at the physical device boundary. Behavior of the devices at their physical boundaries was much more non-linear and this was left unaccounted in linear drift model [3]. Joglekar et al. [94] window function aimed to counter this limitation and generalize the non-linear behavior. The ions mobility is significantly higher in bulk of the memristor, but when it comes closed to boundary, the speed is highly suppressed. Joglekar et al. [94] proposed a modified equation for the linear model including the window function which accounted for this effect, is given as [94]:

112 $$ \frac{dw}{dt}=\eta \frac{\mu_D{R}_{\mathrm{ON}}}{D}i(t)F\left(\frac{w}{D}\right) $$

This modified equation reflects the speed suppression at the edges, i.e., *w~*0 or *w~D*. Here, *F*(*x*) is a window function which satisfies the conditions *F*(0) = *F*(1) = 0 so that there is no drift at the boundaries. The function is also symmetric about x = ½ and increases monotonically over the interval 0 ≤ *x* ≤ 1/2, 0 ≤ *F*(*x*) ≤ 1 = *F*(*x* = 1/2). The window function *F*(*x*) parameterized by a positive integer p, is defined as [94]:

113 $$ F(x)=1-{\left(2x-1\right)}^{2p} $$

**Fig. 20:**
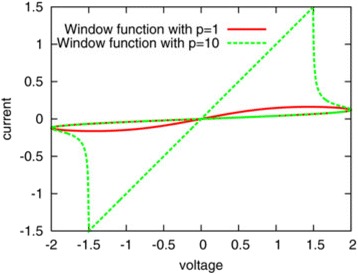
Theoretical *i*-*v* curves for a memristor with (realistic) dopant drift modeled by window functions *F*_*p*_(*x*) = (1 − (2x − 1))^2p^ considering *p* = 1 (red solid) and *p* = 10 (green dashed). An external voltage *v*(*t*) = 2v_0_ sin(*ω*_0_*t*/2) is applied. The memristor parameters are *w*_0_/*D* = 0.5 and *R*_OFF_/*R*_ON_ = 50. The memristive behavior at *p* = 10 has been enhanced. The slope of the *i*-*v* curve at a small time period is the same, *R*_0_^−1^, in both cases whereas the slope on return sweep depends on the window function. For a large *p* value, the return-sweep slope is *R*_ON_^−1^ = 1> > *R*_0_^−1^, corresponding to a fully doped memristor [94]

Simulated results of the window function are shown in Fig. 20 [94]. The results obtained from the window function implementation, it was understood that with a significantly high value of *p*, the function *F*(*x*) provides excellent generalization of the linear drift model^3^ without any of its constraints. So, the window function works best at a high value of p, where it models the linear model accurately and also accounts for the non-linear effects at the boundary. This can be particularly seen in the variation of the function. With an increase in *p*, *F*(*x*) stays constant over an increasing interval around *x* = 1/2 and when *r* → ∞, *F*(*x*) = 1 for all *x* except 0 and 1 which are the boundary conditions. So, Eq. (112) models the general memristive systems perfectly at these particular conditions of *p*.

This characteristic also acts as a significant limitation for the model. On the one hand, at low values of *p*, the window function does not perform as per expectation. But at significantly high values of *p* where the non-linear effects are taken into account, the difference between the linear drift and non-linear drift in the model disappears. This means that there is no proper way to account for both the linear and non-linear drift effects at the same time, while implementing this window function. Also, the mobility at the boundaries was suppressed down to zero, which made the function to be stuck at the 0 value at the terminal states. Shortcomings of this function and the improvements made over it by the Biolek window function [95] are discussed further.

Window function implemented here should be understood as extensions to the physics based models. General limitation with the physics based models is that the models do not account for the effects at the physical boundaries of the device. Memristive devices have been found to behave differently in the bulk and the boundary of the device. So, these window functions can help to overcome these limitations in the device by setting a boundary for the model and properly accounting the boundary effects.

Joglekar et al. [94] also investigated memristive device implementation in standard fundamental circuits along with the other basic elements R, L and C. Combining these four basic elements (memristor, inductor, resistor, and capacitor), the functioning of standard circuits such as MC, MLC, etc. were studied. They came to a similar conclusion as the HP team [3] that the primary property of a memristor is the memory of the charge that has passed through it. The memristor was dimensionally characterized as a magnetic flux *D*^2^/μ_D_ where *D* is the memristor size and μ is the mobility.

### Biolek Window Function

Biolek window function [95] developed by Zdenek and Dalibor Biolek in 2009 was modeled on the proposed memristor equations by Strukov et al. [3] Its primary aim was to provide a marked improvement over the previous Joglekar model [94] and also provided a SPICE implementation of the Linear drift model [3]. They proposed changes to the way the window functions are defined so that a closer approximation between the model and the real circuit element can be achieved. They also reported the SPICE implementation of the linear drift model [3], which opened up the model to a wide range of circuit applications at that time.

Strukov et al. [3] first pin-pointed the pertinent problems in the Joglekar function while implementing in SPICE and then proposed improvements over it. First major problem with Joglekar function is its way of setting up of the terminal state R_ON_ and R_OFF_. State equation and the window function defined, respectively, in Eqs. (113) and (114) bound the value of the variable to 0 at the boundary and it is forced to hold that value. This state cannot even be changed by an external stimulus. This happens due to the HP memristor remembering the *x*-coordinate of the boundary between two layers and not the amount of electric charge passed through it. As a result, when a new set or reset transition is to be started from a terminal value, the device has to start from 0 and not the actual value it had in its previous state.

Second problem of the window function is noticed when the model is implemented as an actual circuit element. The circuit component exactly remembers the entire charge which is passing through it. So, in case of the Joglekar window function, to transpose the memristor from a state *x*_0_ to *x*_1_, a certain amount of charge *q* is required. Now the same amount of charge but in the opposite polarity, i.e., *–q* is required to bring the memristor back from *x*_1_ to *x*_0_. Thus, when a memristor is being driven by a constant current with a time interval, say *t*, the same time *t* is also required for restoring the device back to its original state.

This occurs regardless of the fact that the device could be in its terminal state all the while when the current flows. This leads to significant operating delays as documented by the SPICE simulation presented by Biolek et al. [95]. Also, when the current direction is reversed, the boundaries start to move in an opposite direction regardless of the past state, thus the state is lost along another curve.

Window function being added to a particular model should be able to enhance its accountability for most of the device characteristics and get rid of the arising discrepancies. Window function proposed by Biolek et al. [95] works in this regard, defined by a positive integer *p*, memristor current *i*, and is given as [95]:

114 $$ f(x)=1-{\left(x- stp\left(-i\right)\ \right)}^{2p} $$

115 $$ stp(i)=\left\{\begin{array}{c}1\kern0.5em \mathrm{for}\ i\ge 0\\ {}0\kern0.5em \mathrm{for}\ i<0\end{array}\right. $$

If the function increases the width of the doped layer, or *x* → 1, the current is positive. The function value is 0 at the boundaries. When increasing the value of *p*, the function yields a flat window with steep troughs to zeros at *x* = 0 and *x* = 1.

**Fig. 21:**
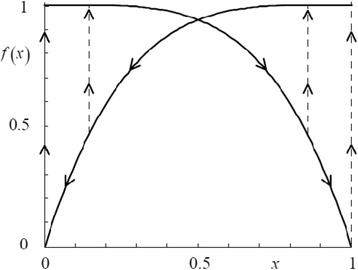
A proposed new window function demonstrated in the model (taking *p* = 2) [95]

Figure 21 [95] shows the simulated results of the window function implementation. Tuning parameter *p* can be used to fine tune the function accordingly to fit to different models. The obtained results satisfy all the conclusions obtained by the linear drift model [3]. The only critical limitation is the inability to account for the hard-switching effects governed by non-linear ionic drift. This means that a symmetrical hysteresis loop obtained in the models cannot be achieved. This is an inherent disadvantage the Biolek [95] window function has in its definition. So, in hindsight none of the functions were truly applicable to the proper extent in the linear drift model [3]. It was a limitation of the time which was further accentuated by the lack of detailed information about the non-linear ionic drift.

### Benderli-Way Window Function

Another window function based on the basic HP model was proposed by Benderli and Wey [96] in 2009. The end result they set out to get was similar to the Biolek function [95]. The developers wanted to develop a SPICE compatible macro model based on the HP memristor which would be suitable for applications in circuit simulations. They proposed a clipping circuit which will bind it within the constraints of the length of the device (*D*).

The proposed clipping circuit was comprised of four comparators whose job was to ensure that the state variable function *w*(*t*) did not go beyond its limits. The comparators clipped *w*(*t*) at its top and bottom boundaries. It basically acts as a switch in which if the comparators detect a certain value in the device they activate a switch and set the device at a particular voltage. So, when *w*(*t*) reached the upper boundary of the device, the device is connected to a voltage source of value *D*, which effectively clips *w*(*t*) at *D*. This operation occurs when the voltage bias is positive.

At negative voltage bias when *w*(*t*) approaches the lower boundary, the circuit is connected to ground, thus clipping *w*(*t*) at 0. This clip is enforced until the voltage polarity is changed suggesting the correct operation is being performed. Non-linear effects at the boundaries are modeled by a proposed window function which takes into account the non-linear dopant drift. The window function is defined as [96]:

116 $$ f(x)=\frac{w(t)\left(D-w(t)\right)}{D^2} $$

Also by increasing the capacitance near the boundaries, the non-linear effects could be accounted for it in the circuit. The major shortcomings of this function are its simplifying approximations and the lack of a clear description of how the linear and non-linear drift can be modeled in the circuit. Although it manages to obtain a hysteresis relation for the device, it suffers from similar limitations as the Joglekar model [94]. Additionally, lack of clear information regarding the non-linear effects was an equal deterrent to the application of the function in circuits.

### Shin Window Function

Shin, Kim, and Kang [97] in 2010 tried to circumvent the issue of window functions by proposing a constitutive relationship derived from the basics of the memristors developed by Chua [1]. This is different from the previously reported window functions in the sense that they tried to model the memristors perfectly by relating charge and flux together. This was the fundamental essence of the Chua model and is a stark contrast to the linear ion drift mechanics proposed by Strukov et al. [3].

Utilizing the relationships between flux (*φ*) and charge (*q*) in a current-controlled and voltage-controlled memristor, the window function developed keeps the model checked within the bounds. Chua model for a current controlled memristor is defined as [97]:

117 $$ \frac{d\varphi}{d t}=\frac{d}{d t}\left[f(q)\right]=\left\{\frac{d}{d q}f(q)\right\}\times \frac{d q}{d t} $$

118 $$ {v}_M=\left\{\frac{d}{dq}f(q)\right\}\times {i}_M\equiv {R}_M(q)\times {i}_M $$

Here *R*_*M*_(*q*) is the memristance defined by a derivative of the charge-flux relationship with respect to the charge. Thus, *R*_*M*_(*q*) = *df* (*q*)/*dq* defines it as a current controlled memristor.

Similar to the above equations, a voltage controlled memristor is defined in terms of the charge-flux relationship *q* = *g* (*φ*) as [97]:

119 $$ \frac{d q}{d t}=\frac{d}{d t}\left[g\left(\varphi \right)\right]=\left\{\frac{d}{d\varphi}g\left(\varphi \right)\right\}\times \frac{d\varphi}{d t} $$

120 $$ {i}_M=\left\{\frac{d}{d\varphi}g\left(\varphi \right)\right\}\times {v}_M\equiv {G}_M\left(\varphi \right)\times {v}_M $$

So, on similar terms, G_M_(*φ*) is a voltage-controlled memductance whose values can be calculated by measuring slopes of charge-flux relationship *g*(*φ*).

The above written Eqs. (118) and (119) can be used in compact models for circuit implementations. But they are inadequate when it is needed to define it within a bounded resistance range. This is where window functions are written to modify the circuit parameters so that the model operates within the resistance range of *R*_MIN_ and *R*_MAX_. Thus, in mathematical terms it means that *R*_M_∈ [*R*_MIN,_*R*_MAX_]. Memristor needs to be confined within the available range of resistance so as to adhere to design requirements. When the device reaches one of its boundary values, it has to stay in that state after any excess charge or flux is applied to the device. This has to be ensured so that the device does not violate its boundary conditions under hard switching conditions.

This condition is obtained in this case by ignoring the excess input current or voltage. So, in the model it is masked by a window function *H* so that the value of q or *φ* always stays within the available range. The masked input current and input voltage in the case of a current controlled and voltage controlled memristor are respectively given by [97]:

121 $$ H\left({i}_M\right)=\left\{\begin{array}{c}{i}_M,\kern5.5em \mathrm{if}\ {R}_M\in \left({R}_{\operatorname{MIN},\kern0.75em }{R}_{\mathrm{MAX}}\right)\\ {}0,\kern0.5em \mathrm{else}\  \mathrm{if}\ {i}_M\ \mathrm{does}\  \mathrm{not}\  \mathrm{pass}\  \mathrm{zero}\end{array}\right. $$

122 $$ H\left({v}_M\right)=\left\{\begin{array}{c}{v}_M,\kern5.5em if\ {R}_M\in \left({R}_{\operatorname{MIN},\kern0.75em }{R}_{\mathrm{MAX}}\right)\\ {}0,\kern0.5em \mathrm{else}\  \mathrm{if}\ {v}_M\ \mathrm{does}\  \mathrm{not}\  \mathrm{pass}\  \mathrm{zero}\end{array}\right. $$

Above equations disregard any excess input current or voltage in the model space. The boundary state of the device is held until the polarity of the input source is reversed. When the polarity is reversed, it indicates the start of a new transition; the function will force the memristor to move back into memristive region. The operation is similar to the clipping circuit proposed in the Benderli-Way function [96] discussed previously, but here, there is no requirement of a complicated comparator circuit. The major purpose of using this approach to model the devices was to remove the usage of a special window function as done previously but still be able to adhere to the boundary conditions implicitly.

But the developers were also wary of the fact that some devices may not be modeled properly by their proposed constitutive relationship, which have a non-constant dynamic state variable. This was made in order to account for the non-linear drift effects discussed and experimentally shown in the HP memristor device [3].Hence, in order to take into account, the non-linear drift effects as well, they proposed a special window function. The window function aimed to overcome the backing problem faced by the Joglekar [94] function discussed previously. It has been described as [97].

123 $$ W(q)=\delta +\left\{1-{\left(2z-1\right)}^{2p}\right\} $$

where δ is a non-zero positive constant (δ < < 1), z is a normalized memristive charge, and *z* defined as *z* = (*q* − *q*_MIN_)/(*q*_MAX_ − *q*_MIN_). The non-zero value of *δ* ensures that the state of the model returns to the normal memristive region when the input polarity is reversed. This removes the backing problem suffered by previous window functions.

### Prodromakis Window Function

Prodromakis window function was proposed in 2011 by Themis Prodromakis et al. [98] of Imperial College, London, which aimed for a simple and efficient function modeled the memristor device characteristics [191] effectively. Some of the limitations and constraints of the previous models were alleviated which made the function easy and accurate to use.

The window function is considered to be parabolic in nature. It also employs a control parameter (*p*) in the exponent which provides the model with the required scalability and flexibility. It also makes the window function *f*(*x*) scale upwards or downwards which helps create a family of distinct curves. The function *f*(*x*) is given in terms of *p* ∈ *R*^+^ as [98]:

124 $$ f(x)=1-{\left[{\left(x-0.5\right)}^2+0.75\right]}^p $$

Control parameter is critical in this function as it helps to remove many of the constraints and limitations of the previous functions. The function can scale upwards due to the control parameter, which suggests that *f*_max_(*x*) can take any value between 0 and 1 inclusive. Also a very large value of *p* provides a linkage with the linear dopant drift effects. A serious limitation with the previous Joglekar [94] and Biolek [95] models was that the control parameter was allowed to take only integer values. But here, *p* could have real values as well which added more flexibility to the model.

Results obtained from the function suggest it returning a zero value at the active bi-layer edges. The drift of the dopants is also suppressed near the metal interfaces. This accounts for the non-linear drift effects at the boundaries. Other major problem of zero value stuck at the terminal state is tackled by implementing a feedback path as suggested in Eq. (123). The function can also be adjusted for any peculiar cases such as when *f*_max_ > 1 with the help of a second control parameter *j* given as [98]:

125 $$ f(x)=j\left(1-{\left[{\left(x-0.5\right)}^2+0.75\right]}^p\right) $$

Hysteresis loop obtained using the window function is asymmetrical which has been explained by Prodromakis et al. [98] as a result of the different switching rates of the ON and OFF rates which is quite reasonable. The hysteresis also suggests there is no terminal state problem as highlighted in the Joglekar function. Width of the doped region does not go higher than *D*, and the memristance is correctly limited. In the case of reverse polarity, it does not get stuck at a zero value and does not take any error states as highlighted by the results.

**Fig. 22:**
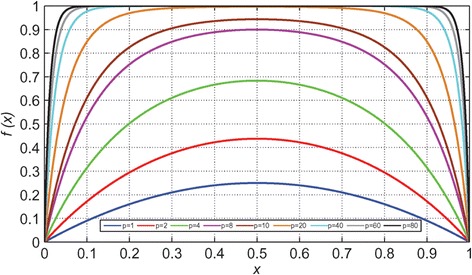
Plot of the proposed window function *f*(*x*) = 1 − [(*x* − 0.5)2 + 0.75]^p^ against the normalized width of the doped region *x* (*p* is considered as a variable) [98]

Results presented by Prodromakis et al., shown in Fig. 22 [98], suggest that the function can be adjusted and scaled effectively using the two control parameters in the function. The *p* parameter supports lateral scaling while the *j* parameter supports vertical scaling. Value of *p* has been increased from *p* = 1 to *p* = 80 and the results are shown in Fig. 22, which showcase the scaling features of the window function. This window function is one of the most efficient and accurate among the others which have been reported so far.

### Boundary Condition-Based Model (BCM) Function

This window function developed in 2012 by Fernando Corinto and Alan Ascoli [99] was aimed at improving the models proposed by Joglekar [94] and Biolek [95]. They identified possible limitations with the previous functions with respect to their exhibition of single-valuedness and multi-valuedness, respectively. Also tuning the range and the boundary conditions were not possible with the Joglekar [94] and Biolek [95] functions. This was handled by the BCM window function by deriving novel methods to propose closed-form solutions for memristor devices. Along with that they also added tuning parameters to increase the flexibility of the boundary conditions used in the models.

Design of the Joglekar function limits it to a single value of memductance-flux characteristics at all input values. Similarly, the input dependent Biolek function limits it to only multi-values of the function under sign varying input. But the BCM function allows for both single-valued and multi-valued memductance-flux characteristics under a single sign varying input. The function assumes a linear dopant drift effect, which simplifies the analytic integration as well as makes it suitable for closed form solutions under any initial condition state. But this invariably neglects the non-linear boundary effects in the device. So, on the one hand, the BCM function proposes a very simplified expression for defining the boundaries of the device but it misses out on accounting for the non-linear effects due to the simplifying assumptions.

BCM model uses tuning parameters in the window function equations in an attempt to account for the non-linear effects [3, 46, 172, 173]. But they are not accurate to the same level as implicit definition of those effects. Another assumption which further simplifies the model to allow for closed form solutions is that the ionic drift rate remains constant. The BCM model is based on a window function having unitary values for all *x*(*t*) ∈ (0,1), and also exhibiting the vertical transitions as [99]:

126 $$ 1\longrightarrow 0\left\{\begin{array}{c}\mathrm{if}\exists t\mid x(t)=1\kern0.5em \mathrm{for}\ \eta v(t)\ge -{v}_{th,1}\\ {}\mathrm{if}\exists t\mid x(t)=0\kern0.5em \mathrm{for}\ \eta v(t)\le {v}_{th,0}\end{array}\right. $$

and

127 $$ 0\longrightarrow 1\left\{\begin{array}{c}\mathrm{if}\exists t\mid x(t)=1\kern0.5em \mathrm{for}\ \eta v(t)<-{v}_{th,1}\\ {}\mathrm{if}\exists t\mid x(t)=0\kern0.5em \mathrm{for}\ \eta v(t)>{v}_{th,0}\end{array}\right. $$

Here, η is a linear control parameter and ∃ is a quantifier denoting “there exists” which signifies that for *x*(*t*) there exists exactly one solution. Values of the non-negative parameters *v*_th,1_ and *v*_th,0_ determine the occurrence of such transitions. The conditions in the first Eq. (125) are established when *x*(*t*) obeys the boundary conditions *x* = 1 and *x* = 0. But the conditions in the second Eq. (126) is established when the function *x*(*t*) no longer obeys these boundaries.

These dynamics are encapsulated in the mathematical description defined by the tunable boundary conditions *C*_*n*_ (*n* = 1, 2, 3) as [99]:

128 $$ {C}_1=\left\{\begin{array}{c}x(t)\in \left(0,1\right)\ \mathrm{or}\ \left(x(t)=0\ \mathrm{and}\ \eta v(t)>{v}_{th,0}\right)\\ {}\mathrm{or}\ \left(x(t)=1\ \mathrm{and}\ \eta v(t)<-{v}_{th,1}\right)\end{array}\right\} $$

129 $$ {C}_2=\left\{x(t)=0\ \mathrm{and}\ \eta v(t)\le {v}_{th,0}\right\} $$

130 $$ {C}_2=\left\{x(t)=1\ \mathrm{and}\ \eta v(t)\ge {v}_{th,1}\right\} $$

So, in case of η = + 1, *v*_th,0_ is the threshold voltage. This is the minimum value of the input needs to cross, after it enters the positive region. Similarly, *v*_th,1_ represents the negative region, for η = − 1. This means that the conditions in Eqs. (127) and (128) have to be met first, before the conditions in Eq. (129) holds. The window function *F* is thus defined as [99]:

131 $$ F\left(x,\eta v\right)=\left\{\begin{array}{c}1;\mathrm{if}\ (127)\ \mathrm{holds}\\ {}0;\mathrm{if}\ (128)\ \mathrm{or}\ (129)\ \mathrm{holds}\end{array}\right. $$

The window function qualitatively works similar to other functions. At the boundary conditions, the vertical transition from 0 to 1 or vice versa occurs depending on the polarity of the input stimulus. Thus, the input used here is sign varying in nature.

## Model Verification

Several ways are there to verify the working of the presented models, in this work. Some of the implementations and verification have been included with the description of the model. Models which have been described quantitatively using mathematical equations can be verified by solving the equations in a simulator. Generally, *I*-*V* characteristics of the simulated model are compared with the corresponding experimental data from the device. This gives a fair idea on the reliability of the model. Physical models described by mathematical equations can be solved by a multitude of solvers such as MATLAB, Mathematica, COMSOL, etc. Compact models which have been translated to work in the circuit level are generally simulated using the SPICE framework. There are a variety of SPICE based simulators in the market such as HSPICE, Ngspice, etc. which could be utilized. Corresponding output characteristics can be matched with the experimental results.

Physically verifying the switching mechanism in a model is trickier. It generally involves in-situ observation of the switching process [192] which requires a lot of precision and high-end equipment. However, it is very solid evidence regarding the viability of the switching mechanism presented in the model. There have been some novel methods reported to observe the growth of the conductive filaments during the switching process. Conductive atomic force microscopy (C-AFM) [182] has been used to visualize the formation and rupture of conducting filaments. *I*-*V* switching curve is shown in Fig. 17, which is clearly shows HRS/LRS states and the corresponding state of the filaments. Electrostatic force microscopy (EFM) [193] can also be used to visualize the migration and accumulation of oxygen ions by calculating the electrostatic force between the probe and the sample. It is one kind of in-situ TEM, where the focus is primarily on the charge of the carrier. Formation of conductive channels can be observed by high resolution energy-dispersive X-ray spectroscopy (EDS) which can provide accurate detailing of the composition in the filaments. These two methods have been proven to be quite effective in verifying the physical switching mechanism and the visualization of conductive filaments in RRAM devices.

## Well-Posed Memristive System Definitions

An excellent work published in 2015 by Wang and Chowdhury [100] of UC Berkeley set a new paradigm for memristor and RRAM modeling. It was a push in the right direction for the whole memristor modeling community. The major features of the work were the significant improvements made on the pre-existing models. Some tweaks were proposed in the fundamental understanding of memristor models which contribute to eradicating some of the long-standing issues which has plagued the memristor models. Also, they demonstrated implementation of the models in SPICE, Verilog-A as well as their own prototyping platform based on MATLAB called as MAPP [191].

The root cause of many of the issues affecting memristor models were improper mathematical implementation. As a result, it limited their application in a variety of simulation and design scenarios. Simulation of the models is a very critical factor in determining the applicability of the model; however, the existing models were unable to be applied in a variety of simulation studies such as DC, transient etc. This work aimed at modifying the models into a form where simulating them would be a simple task.

The common ill-posed or erroneous definitions that many of the previous models suffer are not being properly defined at all biases, outputs not being unique or continuity problems. These basic problems are to be avoided in the models for wide application. All the various problems that the authors have encountered and the improvements they have presented are discussed.

A very valid point highlighted by them is that a well-written model for a particular circuit should be able to replicate its characteristics or be valid beyond its actual boundaries as well. Even if getting outputs beyond the applicable range might not be physically possible, but in simulation environments like SPICE it is imperative that the circuits work at all level of biases. This will lead to efficient simulation and produce smooth varying outputs at all biases. Many of the models we have discussed previously have sought to ignore the operation of the model beyond the available range, leading to their incompatibility for use in circuit simulators. This requirement can also be understood by the underlying algorithms of circuit simulators such as the Newton-Ralphson (NR) algorithm [194] which is commonly used for solving non-linear equations.

The NR algorithm [194] works on the principle of applying a sequence of biases to devices so that convergence can be reached for a valid solution of the circuit. So even if the bias is physically possible or not, for the NR algorithm to find a solution the model must evaluate at all bias. This invariably means that if the NR finds a solution to a well-designed model, the input bias will be physically reasonable. In the cases where the converged solution is physically not possible, it provides insights into the problematic areas of the models and is critical to troubleshooting. Therefore, in order for the NR algorithm to work correctly and find a proper solution, models should be designed to be evaluated at all biases.

Another fundamental problem is the divide-by-zero error. Many of the models have terms such as 1/(*x* − *a*) which cause these errors. It leads to the solutions getting unbounded and causing discrepancies. Along with that, some expressions use square root (√) with negative arguments which give rise to complex arguments. Non-real numbers are not valid arguments for models and can cause non-convergence to a valid solution in simulators.

Almost all of the models we have discussed earlier do not account for a very important aspect of device model simulations. Any mathematically viable input must produce a mathematically viable output, and the most basic among this is the DC analysis. It is commonly the crux and starting point of any analysis and a proper model should produce an accurate DC solution. A proper well-designed model should work consistently with all kind of analysis. But almost all of the models suffer from significant DC response problems. So, this is another area that needs to be improved. Wang et al. [100] also addresses the problems of the models generally faced when being defined in circuit level languages such as Verilog-A.

The crux of the improvement to the models Wang et al. [100] have proposed revolve around the correct way of modeling hysteresis itself, i.e., using internal unknowns and implicit equations. This is because the dynamics of the filament in a RRAM closely follows a hysteresis characteristic. Also, the improvements make the models simulation ready with all the major analyses like DC, AC, and transient providing acceptable results. Various techniques are also proposed to aid convergence in electric simulators including a proposed new limiting function which replaces the functionality of window functions and overcomes all their limitations.

### Accurate Description of Hysteresis

Memristive systems like RRAM devices have been proven to follow the features of hysteresis [1, 2] very closely. So, tweaking the models should start from the very basic. Modeling and defining the hysteresis characteristics accurately are critical to the proper functioning of RRAM models. Devices with hysteresis do not have a simple algebraic mapping between the voltage *v*(*t*) and current *i*(*t*). A state variable *s*(*t*) which defines the state of the device is required for the mapping given as [100]:

132 $$ i(t)={f}_1\left(v(t),s(t)\right) $$

A differential equation is used to govern the dynamics of the state variable, i.e., the rate of change of the state described as [100]:

133 $$ \frac{d}{dt}s(t)={f}_2\left(v(t),s(t)\right) $$

The above equations serve as the model template for modeling hysteresis. Value of the *s*(*t*) at a particular time instant *t* is governed by the history or the state of *v*(*t*). Thus, the device is considered to be having an internal memory of the input voltage. Functions f_1_ and f_2_ are chosen accordingly to define the characteristics of the device. Choice of these functions could be termed as critical in defining the dynamic of the device.

There has been a very common thought that hysteresis shows up in transient analysis only [1–3]. But Wang et al. [100] have demonstrated hysteresis based on the DC solutions of the models. This has been achieved by the proper selection of the governing functions *f*_1_ and *f*_2_. As discussed earlier, obtaining a DC response is imperative for the proper analysis and simulation of the devices. So, obtaining a hysteresis in a DC solution makes this concept highly noteworthy. The governing functions are defined as [100]:

134 $$ {f}_1\left(v(t),s(t)\right)=\frac{v(t)}{R}.\left(\tanh \left(s(t)\right)+1\right) $$

135 $$ {f}_2\left(v(t),s(t)\right)=\frac{1}{\tau }.\left(v(t)-{s}^3(t)+s(t)\right) $$

**Fig. 23:**
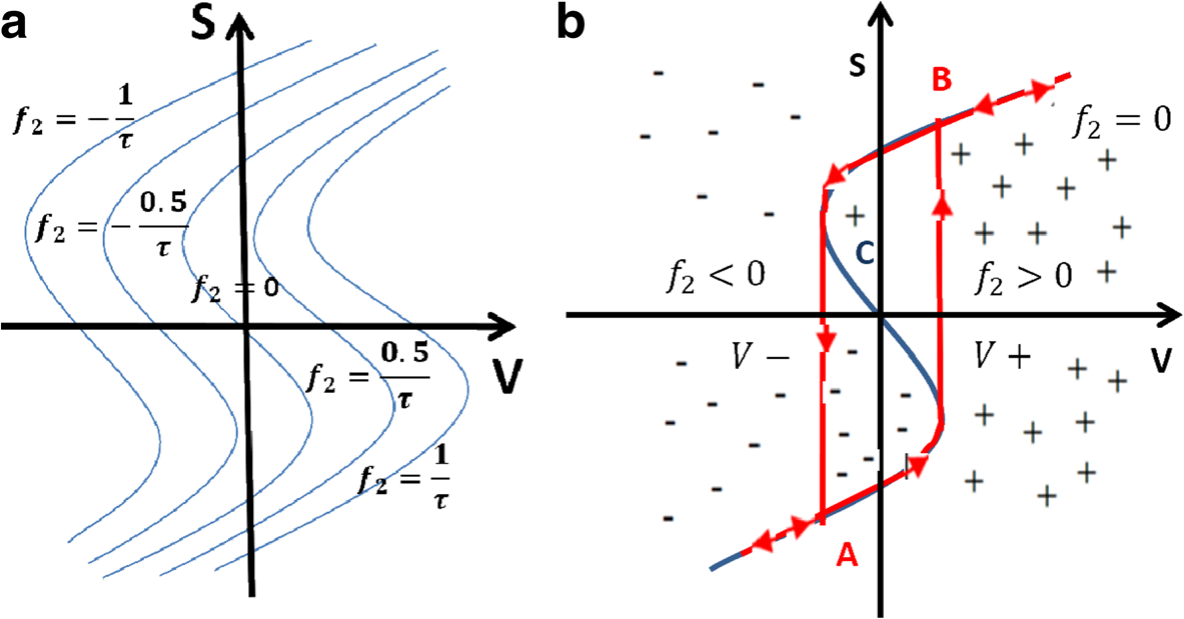
*f*_2_ function in (6) plotted in contour form and predicted *s*-*v* hysteresis curve depending on the sign of *f*_2_ [100]

In the function f_1_, hyperbolic tangent function tanh is chosen because of its monotonically increasing properties with a range of (− 1,1). The dynamics of *s*(*t*) is governed by the choice of *f*_2_. When the value of *f*_2_ is 0, the corresponding (*v*, *s*) values are the DC solution of the circuit. As shown in the contour plot in the Fig. 23a, [100] the curve *f*_2_ = 0 folds back in the middle and crosses the *v* = 0 axis three times. Thus, it has three stable states or three possible values of *s* in the DC solution which forms the foundation of the DC analysis of hysteresis. The operation of the variable s as a hysteresis curve is shown in Fig. 23b [100]. As *s* modulates the current, the *I*-*V* relationship will result in a hysteresis as well. Multiple stability of the state variable and abrupt changes in the DC solutions leads to the formation of a hysteresis in DC analysis. This sets a very strong foundation for accurate and efficient modeling of RRAM devices.

### Proper Definition of Internal Unknown Variables in Verilog-A

It has been discussed several times during review of the various models, implementation of the models accurately in SPICE [116, 117] and Verilog-A [142] is critical for their acceptability. This is because SPICE is the most commonly used circuit simulation platform, and Verilog-A is the most widely used hardware description language. So, simulating in these platforms is as close as it gets to the real physical devices. A major shortcoming of the previous models is the way that internal unknown variables were handled in Verilog-A.

In a memristor model, the state variable is an internal unknown because its value gets changed with different states. Verilog-A does not have a straightforward way of handling and defining these unknowns. As a result, it can get very confusing while dealing with a constantly changing value. Wang et al. [100] proposed to declare the state variable *s* as a voltage or potential rather than any “real value.” Some very critical points are mentioned below while handling internal unknowns in Verilog-A.

- Different Verilog compilers handle variables declared using “real” differently. Then, this will lead to very inconsistent results.
- Differential equations should not be defined by using the in-built idt() function. Because this function has very inconsistent support in the compilers and causes many limitations [140, 142].
- Time integration to obtain analytical solutions should not be coded inside the model. The process is pretty simple but has serious pitfalls as given below.

- ➢This method makes use of “abstime” function. To define the starting point of the integration it also uses “initial_step.” These have been termed as bad modeling practices in analog simulation [140, 143].
- ➢The internal unknowns are defined as a memory state in this method, which can create problems for periodic steady state (PSS) analysis.
- ➢This method bypasses many of the simulators built in facilities such as the convergence aids, time step control etc.
- ➢It can cause serious convergence issues for stiff systems due to its dependence on the Forward Euler (FE) algorithm [195].

These problems are generally a combination of bad modeling practices and the incapability of Verilog-A to handle internal unknowns efficiently. As a result, declaring *s* as a voltage has been demonstrated as an effective way of getting around the problem.

### Developing Generic RRAM Models

Taking the previously discussed hysteresis equations as a template, Wang et al. [100] presented a generic way of developing compact models for RRAM devices. To demonstrate the RRAM model, ASU/Stanford model [78, 80] is considered.

The filament gap is used as state variable. Current across the device is considered as *itb*, the voltage as *vtb* and the unknown state variable as *gap*. The equations are defined based on the previously stated hysteresis template given as [100]:

136 $$ itb(t)={f}_1\left( vtb(t), gap(t)\right) $$

137 $$ \frac{d}{dt} gap(t)={f}_2\left( vtb(t), gap(t)\right) $$

The above equations define the physical contexts of the RRAM device. Now, choosing the proper functions for *f*_1_ and *f*_2_ is critical to capture the physical properties. In most of the models, we have encountered till now the application of consistent equations for *f*_*1*_ with some changes in the internal unknowns. This fact is corroborated by Wang and Roychowdhury’s work [100]. They have considered the *f*_*1*_ function as [100]:

138 $$ {f}_1\left( vtb, gap\right)={i}_0\times \exp \left(-\frac{gap}{g_0}\right)\times \sin h\left(\frac{vtb}{V_0}\right) $$

The *f*_2_ function is considered according to the ASU/Stanford model [78]:

139 $$ {f}_2\left( vtb, gap\right)={v}_0\times \exp \left(-\frac{E_a}{V_T}\right)\times \sinh \left(\frac{vtb\times \gamma \times {a}_0}{t_{ox}\times {V}_T}\right) $$

140 $$ \gamma ={\gamma}_0-\beta \times {gap}^3 $$

The γ here is the local field enhancement factor [196] which contributes to the abrupt SET (filament growth) and gradual RESET (filament dissolution). A common property among most of the RRAM models is the fact that the sign of *f*_2_ is same as that of –sinh (*vtb*). This means in terms of the gap that it starts to decrease whenever *vtb* is positive and vice versa. But this growth or dissolution cannot be indefinite for numerical simulation to work. For the simulations to work in reality, they have to be bounded which has been discussed in depth in the next section.

Various methods have been proposed to account the boundary effects of the devices. It will come up short with the methods having some serious limitations. Some of the models have implemented direct “if-then-else” statements in the Verilog code [80, 81]. But the problem is that the use of “if-else” statements removes the model from the differential equations framework which is not acceptable. It also introduces hard discontinuities in the model whereas we need smooth continuous curves.

**Fig. 24:**
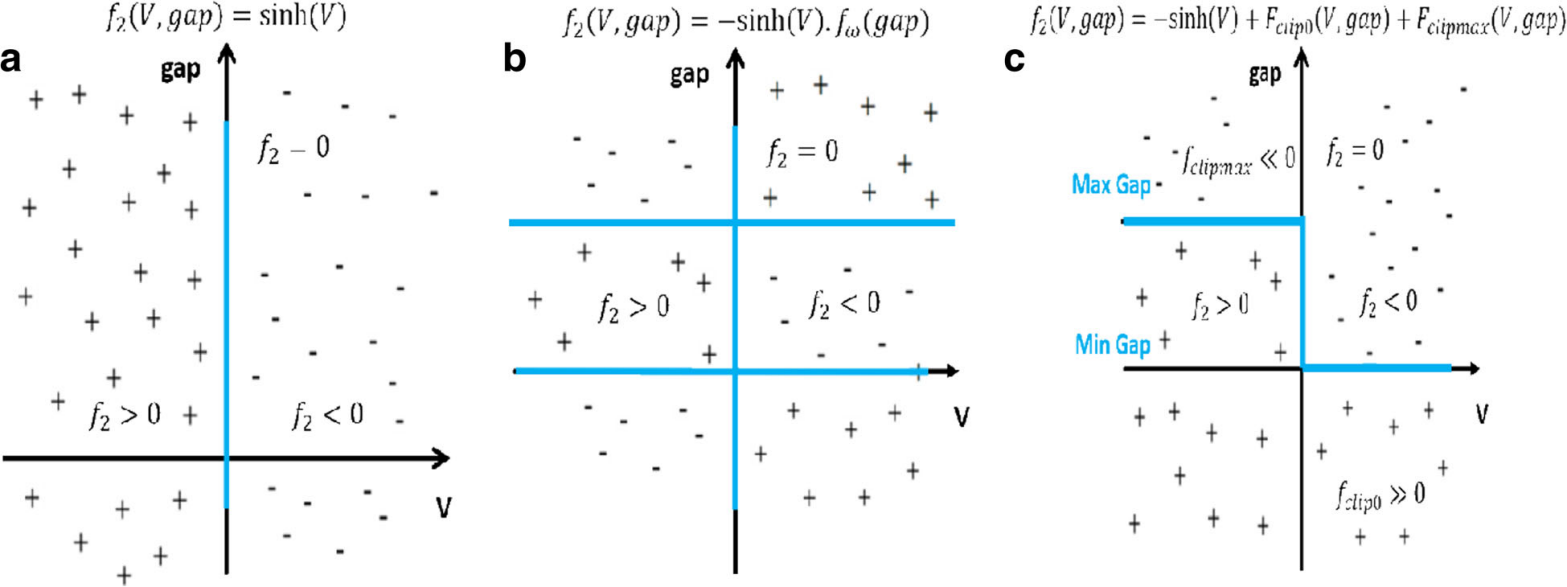
Different choices of *f*_2_ considered in the RRAM model [100]

A very popular way of modeling boundary conditions is the use of window functions which we have discussed in the “Window Function Models” section. The window functions as discussed work on the principle of setting *f*_2_ = 0 when gap = *maxGap* and *minGap*. We have seen that improvements made with the window functions make them suitable for transient simulations and they produce smooth and continuous results. But the real problem with these functions is actually deep-rooted. The problem can be understood by analyzing the sign and zero-crossings of the *f*_2_ curve as shown in Fig. 24 reported by Wang and Chowdhury [100].

As is seen in Fig. 24b, the *f*_2_ = 0 curve has three lines, *V* = 0, *maxGap*, and *minGap*. Beyond the values of *maxGap* and *minGap*, there will not be any stable DC solutions so they will not show up in transient analysis. Therefore, when sweeping between those values, the transient solution will work fine with the window function multiplied to *f*_2_. But with the other analyses there are several problems. In DC operating point analysis, unphysical solutions can show up owing to the fact that all the lines consisting of the *f*_2_ = 0 curves are valid. So, when the voltage is zero, any arbitrary value can be the value of *gap* and it no longer follows the boundaries. Hence, DC analysis is a major limitation for the window functions.

This has been tackled very efficiently by Wang et al. [100] by keeping the DC solutions in a single continuous curve and then trying to bind the value of *gap*. As illustrated in Fig. 24c, curves A and C contain the stable solutions and B has the unstable solutions. So, when the sweeping starts after zero, the value of *gap* will switch between *maxGap* and *minGap*. It is mathematically represented by introducing a couple of clipping functions *F*_clipmin_ and *F*_clipmax_ represented as [100]:

141 $$ {f}_2^{\ast}\left(\mathrm{vtb},\mathrm{gap}\right)={f}_2\left(\mathrm{vtb},\mathrm{gap}\right)+{F}_{\mathrm{clipmin}}\left(\mathrm{vtb},\mathrm{gap}\right)+{F}_{\mathrm{clipmax}}\left(\mathrm{vtb},\mathrm{gap}\right) $$

142 $$ {F}_{\mathrm{clip}\mathrm{min}}\left(\mathrm{vtb},\mathrm{gap}\right)=\left(\mathrm{safeexp}\left({K}_{\mathrm{clip}}\times \left(\mathrm{minGap}-\mathrm{gap}\right),\mathrm{maxslope}\right)-{f}_2\left(\mathrm{vtb},\mathrm{gap}\right)\right)\times {F}_{w1}\left(\mathrm{gap}\right) $$

143 $$ {F}_{\mathrm{clip}\mathrm{max}}\left(\mathrm{vtb},\mathrm{gap}\right)=\left(-\mathrm{safeexp}\left({K}_{\mathrm{clip}}\times \left(\mathrm{gap}-\mathrm{maxGap}\right),\mathrm{maxslope}\right)-{f}_2\left(\mathrm{vtb},\mathrm{gap}\right)\right)\times {F}_{w2}\left(\mathrm{gap}\right) $$

The functions *F*_w1_ and *F*_w2_ are smoother versions of the step functions. This serves the purpose of adhering to the property required for a clipping function to work properly while maintaining a smooth continuous curve. These are described as:

144 $$ {F}_{w1}\left(\mathrm{gap}\right)=\mathrm{smoothstep}\left(\mathrm{minGap}-\mathrm{gap},\mathrm{smoothing}\right) $$

145 $$ {F}_{w2}\left(\mathrm{gap}\right)=\mathrm{smoothstep}\left(\mathrm{gap}-\mathrm{maxGap},\mathrm{smoothing}\right) $$

The functions *safeexp*() and *smoothstep*() are smoother versions of the normal variants of the *exp*() and *step*() function. They have been developed by Wang et al. [100] in their MAPP [191] platform and is available to use within the platform. The clipping functions here closely mimic the actual physical effects occurring in the device. It can be termed as a huge force which keeps the state variable *gap* within its acceptable physical limits.

The templates provided by Wang and Chowdhury [100] for RRAM modeling is capable of widespread applicability and can be used as an ideal platform to develop other models. It consists of accurate modeling of hysteresis, includes proper handling of internal unknowns in Verilog-A and does not need to use incompatible functions like “idt()” and “initial_step” in the differential equation framework. They also circumvent the various limitations of the window functions by the use of mathematically accurate clipping functions. The model templates support a variety of analyses such as DC, AC, transient, PSS, and homotopy [197] in Verilog-A, SPICE, and MAPP. This is a very exhaustive list of advantages which should be used for development of future models by the RRAM community.

### Improving Solution Convergence

Obtaining solution convergence is one of the most important features, every RRAM model ought to possess. The convergence of the solution points to the fact that it is valid and acceptable. This has been a problem for many of the compact models describing RRAM devices. Several techniques have been proposed [100] to aid convergence of solutions in these models. The use of limiting functions compatible with SPICE is very important so that it limits the solutions whenever they cross the acceptable range. A very simple way to make sure the solutions converge is to properly scale the unknowns and variables. Proper scaling makes sure that any results obtained are defined relatively accurate to the inputs.

But the major feature that aids convergence is the use of proper limiting functions in SPICE. Generally during DC operating point analysis, the Newton Raphson (NR) [194] algorithm iterations take very large values while guessing the DC solutions. This is because of the fast-growing *sinh* functions used in the models. This in turn leads to large values of *f*_1_ and *f*_2_. Limiting functions are the best technique to circumvent this and prevent the NR iterations from taking large input values but keeping the *sinh* functions intact. Presently, SPICE includes *pnjlim*, *fetlim*, and *limvds* as limiting functions. But these are not enough to obtain numerical accuracy for RRAM models. A new limiting function dubbed as *sinhlim* has been proposed for this purpose. The function is based on the original *pnjlim* and is given as [100]:

146 $$ {x}_{\mathrm{lim}}=\mathrm{sinhlim}\left({x}_{\mathrm{new}},{x}_{\mathrm{old}},k\right)=\frac{1}{k}\times \ln \left({y}_{\mathrm{lim}}+\sqrt{1+{y}_{\mathrm{lim}}^2}\right) $$

The major feature here is that the limiting function does not use very large values of *x*_*new*_, instead it increases the value in iterations. This function is fully compatible with SPICE and can be implemented in any SPICE compatible circuit simulator. This marks a new addition to the number of limiting functions available for use in circuit simulators apart from the ones developed decades ago.

### Improving Existing Models

On the basis of the accurate generic model templates discussed in the previous section, Wang and Chowdhury [100] have proposed some improvements in some of the existing popular models. We discuss them here and present it in a concise form so that it becomes easy to understand the changes and implement it forward in future models.

The improvements have been proposed on the linear ion drift [3], non-linear ion drift [46], Yakopcic [73, 74], TEAM/VTEAM [75–77], and ASU [78] models. Many of the models have *I-V* relationships in common with other models. Therefore, the most common and important among those are considered and termed as *f*_1_ functions as discussed earlier. The state variable equations of those models are termed as *f*_2_ functions are corresponding improvements are proposed.

Both the *f*_1_ and *f*_2_ functions are generally non-linear and asymmetric. And the reported models use very discontinuous and fast-growing terms like exponential, *sinh*, and *power* (*pow*) functions which results in difficulties during the convergence of the solutions. So, these can be overcome by using “smooth” and “safe” functions as proposed by Wang et al. [100]. The smooth functions are used in place of discontinuous functions. Major design criteria in the smooth functions used is a common smoothing factor which combines common elementary functions to approximate the original non-smooth functions. Smoothing factor controls the trade-off between better approximation and more smoothness. The safe functions are versions of the fast-growing functions which limit the maximum slope the functions can attain, and then linearize it to keep the slopes constant beyond it. For some functions like *sqrt*, *log*, etc., the “safe” versions clip the inputs using *smoothclip* so that non-valid outputs can be avoided.

In the particular f_1_ and f_2_ functions used in the models, the *if-else* statements are replaced by *smoothswitch* which removes the discontinuity of the former. The *exp* and *sinh* functions are correspondingly replaced by *safeexp* and *safesinh*. The authors have demonstrated the definition of the functions in MAPP and Verilog-A which makes it easy for future model developers to integrate it into their system. A very common problem with the *f*_2_ functions, i.e., the state variable dynamics is the uncertainty over the range of the internal unknown. The previous models counter this by either introducing window functions that bound it within a range or do not account for the effects at all. This has been very efficiently handled by introducing self-modeled clipping functions which define the acceptable range of the internal unknown.

Another major problem which is countered is the poor DC hysteresis response of the models. There have been some attempts in the Yakopcic and TEAM/VTEAM models to model this effect by setting the value of *f*_2_ = 0 within a certain voltage range. It has been discussed that DC hysteresis occurs due to the DC solution curve folding backwards. With the approach used in the other two models, when *f*_2_ = 0 with the voltage close to 0, there are infinite number of solutions for the state variables within that voltage range. This is a problematic approach in those unstable DC solutions are also included here which makes the equation system ill-conditioned. This will also cause problems during DC operating point analysis and homotopy analysis. The DC solutions of the models will vary from simulator to simulator because of the manner the equations are designed. This has been very efficiently handled and circumvented by Wang and Chowdhury [100] as summarized in Tables 3 and 4.

**Table 3:** Improved *I*-*V* relationships of the various models

Model	Original *I*-*V* relationship (*f*_1_)	Improved *I*-*V* using concepts from Wang and Roychowdhury^100^
Linear ion drift [3]	*f*_1_ = (*R*_ON_ × *s* + *R*_off_ × (1 − *s*))^−1^ × *vpn*	Can have division by zero error when *s = R*_off_*/*(*R*_on_*/R*_off_). Modified equation: *y* = smoothclip(*s* − *R*_off_/(*R*_on_ − *R*_off_), smoothing) + *R*_off_/(*R*_on_ − *R*_off_) Then, *f*_1_ = (*R*_on_ × *y* + *R*_off_ × (1 − *y*))^−1^ × *vpn*
Non-linear ion drift [46, 68]	*I* = *s*^*n*^*β* sinh(*α* × *vpn*) + *χ*(exp(*γ* × *vpn*) − 1)	sinh can be changed to *safesinh()*, exponential function to *safeexp()*
Yakopcic [73, 74]	$$ I(t)=\left\{\begin{array}{c}{A}_1\times s\times \sinh (Bvpn),\kern0.5em vpn\ge 0\\ {}{A}_2\times s\times \sinh (Bvpn),\kern2.25em vpn<0\end{array}\right. $$	sinh is changed to *safesinh()*. The function is then smoothed. *f*_1*p*_ = *A*_1_ × *s* × *safesinh*(*B* × *vpn*, *maxslope*) *f*_1*n*_ = *A*_2_ × *s* × *safesinh*(*B* × *vpn*, *maxslope*) *f*_1_ = *smoothswitch*(*f*_1*n*_, *f*_1*p*_, *vpn*, *smoothing*)
TEAM/VTEAM [75–77]	$v(t)={R}_{\mathrm{ON}}{e}^{\left(\lambda /{x}_{\mathrm{off}}-{x}_{\mathrm{on}}\right)\left(x-{x}_{\mathrm{on}}\right)}\times i(t)$	The exponential function is changed to *safeexp()*
ASU/Stanford [78–81]	$I\left(g,V\right)={I}_0\exp \left(\frac{-g}{g_0}\right)\sinh \left(\frac{V}{V_0}\right)$	The gap is expressed using s: *gap* = *s* × *min gap* + (1 − *s*) × *maxgap* Then sinh is changed to *safesinh()*, exponential function to *safeexp()*

**Table 4:** The state variable equations presented in an improved form

Model	Original state variable dynamics (f_2_)	Modified using concepts from Wang and Roychowdhury^100^
Linear ion drift [3]	*f*_2_ = *μ*_*v*_ × *R*_*on*_ × *f*_1_(*vpn*, *s*)	DC hysteresis not present. Clipping technique is used to set bounds for s, so that 0 ≤ s ≤ 1
Non-linear ion drift [46, 68]	*f*_2_ = *a* × *vpn*^*m*^	DC hysteresis not present. Clipping technique is used to set bounds for *s*, so that 0 ≤ *s* ≤ 1
Simmons tunneling barrier [70–72]	$$ {f}_2=\left\{\begin{array}{c}{c}_{\mathrm{off}}\times \sinh \left(\frac{i}{i_{\mathrm{off}}}\right)\times \exp \left(-\exp \left(\frac{s-{a}_{\mathrm{off}}}{w_c}-\frac{i}{b}\right)-\frac{s}{w_c}\right),\mathrm{if}\ i\ge 0\\ {}{c}_{\mathrm{on}}\times \sinh \left(\frac{i}{i_{\mathrm{on}}}\right)\times \exp \left(-\exp \left(\frac{a_{\mathrm{on}}-s}{w_c}+\frac{i}{b}\right)-\frac{s}{w_c}\right),\mathrm{otherwise}\end{array}\right. $$ where *i* = *f*_*1*_ (*vpn, s*)	No DC hysteresis present. Consists of fast growing functions. sinh is changed to *safesinh*(), exp to *safeexp*(). Smoothing is performed and bounds for s, so that 0 ≤ *s* ≤ 1
TEAM/VTEAM [75–77]	$$ {f}_2=\left\{\begin{array}{c}{k}_{\mathrm{off}}\times {\left(\frac{vpn}{v_{\mathrm{off}}}-1\right)}^{a_{\mathrm{off}}},\mathrm{if}\ vpn>{v}_{\mathrm{off}}\\ {}{k}_{\mathrm{on}}\times {\left(\frac{vpn}{v_{on}}-1\right)}^{a_{\mathrm{on}}},\mathrm{if}\ vpn<{v}_{\mathrm{on}}\\ {}0,\kern10.25em \mathrm{otherwise}\end{array}\right. $$	The equation is redesigned as: $$ {f}_2=\left\{\begin{array}{c}{k}_{\mathrm{off}}\times {\left(\frac{vpn-{v}^{\ast }}{v_{\mathrm{off}}}\right)}^{a_{\mathrm{off}}}, if\ vpn>{v}_{\mathrm{off}}\\ {}{k}_{\mathrm{on}}\times {\left(\frac{vpn-{v}^{\ast }}{v_{\mathrm{on}}}\right)}^{a_{\mathrm{on}}},\kern2.75em \mathrm{otherwise}\end{array}\right. $$ where *v*^∗^ = (1 − *s*) × *v*_off_ + *s* × *v*_on_, Such that when *s* = 1 and *s* = 0, it is equivalent to the VTEAM equation in the *vpn* > *v*_off_ and vpn < v_on_ regions, respectively. The functions are also smoothened by: ${f}_{2p}={k}_{\mathrm{off}}.{\left( vpn-{v}^{\ast }/{v}_{\mathrm{off}}\right)}^{\alpha_{\mathrm{off}}}$, ${f}_{2n}={k}_{\mathrm{on}}.{\left( vpn-{v}^{\ast }/{v}_{\mathrm{on}}\right)}^{\alpha_{\mathrm{on}}}$, *f*_2_ = smoothswitch(*f*_2*n*_, *f*_2*p*_, *vpn* − *v*^∗^, smoothing) The bounds for s are set using clipping techniques.
Yakpocic [73, 74]	*f*_2_ = *g*(*vpn*) × *f*(*s*), where $$ g(vpn)=\left\{\begin{array}{c}{A}_p\times \left(\exp (vpn)-\exp \left({V}_p\right)\right),\kern0.75em if\ vpn>{V}_p\\ {}-{A}_n\times \left(\exp \left(- vpn\right)-\exp \left({V}_n\right)\right),\kern0.75em if\ vpn<-{V}_n\\ {}0,\kern13.00em \mathrm{otherwise},\end{array}\right. $$ and $$ f(s)=\left\{\begin{array}{c}\exp \left(-{\alpha}_p\times \left(s-{x}_p\right)\right),\kern0.75em if\ s\ge {x}_p\\ {}\exp \left({\alpha}_n\times \left(s-1+{x}_n\right)\right),\kern0.75em if\ s\le 1-{x}_n\\ {}1,\kern2.5em \mathrm{otherwise}\ \end{array}\right. $$	The equations are designed to get proper DC hysteresis: $$ g(vpn)=\left\{\begin{array}{c}{A}_p\times \left(\exp (vpn)-\exp \left({v}^{\ast}\right)\right),\kern0.75em if\ vpn>{v}^{\ast}\\ {}-{A}_n\times \left(\exp \left(- vpn\right)-\exp \left({V}_n\right)\right),\kern0.75em otherwise\end{array}\right. $$ where *v*^∗^ = − *V*_*n*_ × *s* + *V*_*p*_ × (1 − *s*) Also exponential function is changed to safeexp(). The whole function is made smooth. Clipping is used to set bounds for *s*.
ASU/Stanford [78–81]	${f}_2=-{v}_0\times \exp \left(-\frac{q\times {E}_a}{k\times T}\right)\times \sinh \left(\frac{vpn\times \gamma \times {a}_0\times q}{k\times T\times {t}_{ox}}\right)$ where *γ* = *γ*_0_ − *β*_0_ × *Gap*^3^	The *d/dt Gap* is converted to *d/dt s*: ${f}_2=\left( maxGap- minGap\right)\times {v}_0\times \exp \left(-\frac{q\times {E}_a}{k\times T}\right)\times \sinh \left(\frac{vpn\times \gamma \times {a}_0\times q}{k\times T\times {t}_{ox}}\right)$ Also exp is changed to safeexp() and sinh tosafesinh (). Clipping is used to set bounds for *s*.

## Novel RRAM Applications

There have been several new breakthroughs with RRAM architectures and applications. Among them noteworthy in case of architectures is the use of materials such as graphene, amorphous carbon films, transition metal dichalcogenides (TMDs), black phosphorous in a RRAM device. Neuromorphic computing is a novel application scheme for RRAM devices which utilize the memory retention property to use them as synapse devices.

RRAM devices based on graphene and related materials [198] have showcased performance similar to conventional metal oxide devices. These devices are different due to their unique lattice structure and belong to a completely different family of materials. So, investigation on modeling of such devices is highly necessary. Whether conventional modeling techniques such as the ones presented in this work can be used for these devices depends on the physical phenomena governing them and the corresponding *I*-*V* charcateristics. The hypotheses presented to explain the switching in graphene oxide (GO) based devices [198] are consistent with standard bipolar RRAM switching mechanism. The absorption and creation of the conductive filaments are thought to be a result of diffusion of metallic ions from the electrode to the switching layer or transport of oxygen related carriers in the switching media. RRAM devices based on amorphous carbon [198] as the switching media are thought to operate under a similar mechanism. Owing to the similarity in the nature of the physical transport mechanisms, existing physical models can be used to explain the novel GO and amorphous carbon based devices.

Neuromorphic computing [199] is a novel architecture scheme which employs RRAM as synapse devices as its fundamental building block. It is believed that these RRAM based neuromorphic systems can replicate how our brain functions, harnessing the ability of memristors to remember their state. This enables the system to be trained for specific applications just like the human brain. With RRAM forming the crux of these systems, it is critical that device characteristics for the RRAM devices are well modeled. But modeling of RRAM based synapse devices is challenging owing to the fact that the RRAM devices used need well-defined analog behavior, which is the precondition for brain like functions. Device performance under AC stress and cycle to cycle variability are factors which affect the potentiation and depression of the synapse device. Standard models reported in this work can predict the digital behavior of the RRAM, but one may implement them for the analog behavior. Though a few models are reported [200, 201] to describe the switching mechanism in analog RRAM, but it has been difficult to mathematically define them and translate it into a compact form. However, significant research is ongoing to quantitatively describe these characteristics and translate it into a compact form to be used on the circuit level.

## Conclusions

In summary, the important features of all widely accepted RRAM models have been discussed. This work fulfills the requirement of the modeling community for a unified discussion on the various RRAM models. Many of the recent models, such as Stanford/ ASU model, Gonzelez-Cordero et al. model, Prodromakis model, have provided apt explanations for RRAM processes based on the early models. Implementations of different window functions like Joglekar, Biolek, and Prodromakis have been presented and compared. Various unexplained phenomena occurring in the devices are numerically validated in the models. No one model can be deemed as the perfect one, owing to the variety of materials, fabrication processes and device operations exist in the RRAM devices. Each model has been tuned accordingly to fit the device used. Researchers are still some time away from developing a generic RRAM model owing to these factors and also due to the deficiencies in the modeling techniques. Accurate and well-defined modeling techniques have been discussed in the “Well-Posed Memristive System Definitions” section, which should act as a competent template for future model development. Combined with the detailed analysis provided for past RRAM models, this review work can potentially act as a focal point for RRAM model developers.
